# Not only climate: The importance of biotic interactions in shaping species distributions at macro scales

**DOI:** 10.1002/ece3.9855

**Published:** 2023-03-20

**Authors:** Francesca Cosentino, Ernest Charles James Seamark, Victor Van Cakenberghe, Luigi Maiorano

**Affiliations:** ^1^ Department of Biology and Biotechnologies “Charles Darwin” Sapienza University of Rome Italy; ^2^ AfricanBats NPC Centurion Republic of South Africa; ^3^ FunMorph Lab, Department of Biology University of Antwerp Antwerp Belgium

**Keywords:** Africa, bats, biotic interactions, climate, species distribution modeling, trophic guild

## Abstract

Abiotic factors are usually considered key drivers of species distribution at macro scales, while biotic interactions are mostly used at local scales. A few studies have explored the role of biotic interactions at macro scales, but all considered a limited number of species and obligate interactions. We examine the role of biotic interactions in large‐scale SDMs by testing two main hypotheses: (1) biotic factors in SDMs can have an important role at continental scale; (2) the inclusion of biotic factors in large‐scale SDMs is important also for generalist species. We used a maximum entropy algorithm to model the distribution of 177 bat species in Africa calibrating two SDMs for each species: one considering only abiotic variables (noBIO‐SDMs) and the other (BIO‐SDMs) including also biotic variables (trophic resource richness). We focused the interpretation of our results on variable importance and response curves. For each species, we also compared the potential distribution measuring the percentage of change between the two models in each pixel of the study area. All models gave AUC >0.7, with values on average higher in BIO‐SDMs compared to noBIO‐SDMs. Trophic resources showed an importance overall higher level than all abiotic predictors in most of the species (~68%), including generalist species. Response curves were highly interpretable in all models, confirming the ecological reliability of our models. Model comparison between the two models showed a change in potential distribution for more than 80% of the species, particularly in tropical forests and shrublands. Our results highlight the importance of considering biotic interactions in SDMs at macro scales. We demonstrated that a generic biotic proxy can be important for modeling species distribution when species‐specific data are not available, but we envision that a multi‐scale analysis combined with a better knowledge of the species might provide a better understanding of the role of biotic interactions.

## Graphical abstract

Traditionally abiotic factors are considered the main drivers of biodiversity at macro‐scales, whereas biotic factors are considered important almost only at local scales. Modeling the distribution of 177 African bat species, we demonstrated the importance of considering biotic interactions in SDMs at macro‐scales regardless of the species’ ecology.

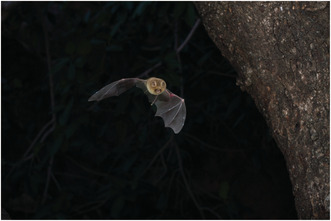

## 1 INTRODUCTION

The distribution of animals and plants is linked to many different components, going from evolutionary history to abiotic (e.g., climate, land cover, topography) and biotic (e.g., parasitism, competition, predation) factors. Traditionally, abiotic factors like climate are considered the main drivers of biodiversity at macro scales (from regional to continental), whereas biotic factors are considered important almost only at local scales (e.g., from landscape to individual home‐range) (Pearson & Dawson,  2003; Soberón, 2007). In particular, the productivity hypothesis postulates that energy and water availability are the main factors that explain the spatial distribution of biodiversity richness across broader scale (Hawkins et al., 2003; Whittaker, 1975). Often, this is translated into calibrating models with variables such as mean temperature and precipitation (Thuiller et al., 2004) without considering any type of biotic factors.

However, biotic interactions clearly have a direct influence on species' spatial patterns with many different mechanisms, going from predation to competition, from resource–consumer interactions to host–parasite interactions (e.g., Bascompte, 2009). Gilman et al. (2010) suggested that species interactions can strongly influence how climate change affects species distribution at every scale and failing to incorporate these interactions in species distribution models certainly limits our ability to predict species responses to climate change. This is true particularly for positive interactions (e.g., facilitation) which may be detectable at large scales, while negative interactions (e.g., competition), being more scale‐dependent, are often assumed to be important only at local scales (Araújo & Rozenfeld, 2014; Belmaker et al., 2015).

Wisz et al. (2013) reviewed the literature on interspecific interactions searching for evidence of their importance in shaping large‐scale species distributions. They found few empirical studies (e.g., Araújo & Luoto, 2007; Heikkinen et al., 2007; Koenig & Haydock, 1999) mostly focused on a limited set of species with “obligate” interactions (e.g., butterfly‐plants). For example, Araújo and Luoto (2007) modeled the distribution of the clouded Apollo butterfly (*Parnassius mnemosyne*) by using climate variables only, climate variables plus the occurrence of four larval host plants as biotic variable, and biotic variables only. According to their findings, the inclusion of a biotic interaction can significantly alter species' distribution at macro scales for both the current time and under future climate change scenarios. These results were not unexpected, since the study is focused on one butterfly species highly dependent on the three host plant species during its larval stage.

In the last few years, only a handful of studies have been added, focusing on trophic (Arumoogum et al., 2019), competitive (Labadessa & Ancillotto, 2022; Stephenson et al., 2022), and animal–host interactions (González‐Salazar et al., 2013). Alaniz et al. (2020) provided an interesting example considering the Magellanic woodpecker (*Campephilus magellanicus*) and its preys in South America. They demonstrated that the inclusion of biotic interactions (specifically the distribution of prey species) helped in defining the niche and distribution of a specialist predator at the continental scale. All these analyses have been performed considering relatively simple systems, with a good level of knowledge, and focusing on specialist species, leaving an open discussion on the generalizability of their results.

Braga et al. (2019) extended these results considering a food‐web database to model the distribution of terrestrial vertebrates in Europe. However, these analyses rely on food‐web data or more generically on biotic interactions data, which are often not available in many regions of the world (the so‐called Eltonian shortfall; Hortal et al., 2015), especially in areas with a high level of biodiversity and limited knowledge of complex trophic interaction webs, such as the African continent.

Several studies overcame the paucity of data using proxies for biotic interactions like, for example, richness of prey species (Aragón & Sánchez‐Fernández, 2013; de Araújo et al., 2014; Gherghel et al., 2018). All these studies found that proxies for biotic interactions can be important in modeling species distributions at large scales, but all of them focused on a single predator and its preys (e.g., Aragón & Sánchez‐Fernández, 2013) or on a limited set of species within peculiar ecological systems (e.g., 5 species of sea kraits in South‐East Asia; Gherghel et al., 2018).

Here, we investigate the importance of including biotic variables in large‐scale species distribution models while considering many species with very different trophic ecology. We modeled the distribution of all bat species occurring in Africa calibrating two SDMs for each species: one “traditional” SDM calibrated with abiotic variables only (hereafter noBIO‐SDM) and one SDM calibrated with both biotic and abiotic variables (hereafter BIO‐SDM). We compared variable importance in the two sets of SDMs to test two main hypotheses: (1) biotic factors in SDMs can have an important role at the continental scale and (2) the inclusion of biotic factors in large‐scale SDMs is important also for generalist species.

Bats represent one of the most successful radiations among mammals, with more than 1400 species, a global distribution (except for the polar regions), and a huge variety of ecological niches (Simmons, 2005). Their trophic ecology covers a huge diversity in both the food items consumed (e.g., plants, arthropods, or vertebrates) and the degree of dietary specialization. In addition, they are often sensitive to climatic and environmental variation (Cooper‐Bohannon et al., 2016; Schoeman et al., 2013), making them an interesting case study for testing our hypotheses.

## 2 METHODS

### 2.1 Species distribution data

We considered all 314 species of bats present in Africa (Wilson & Mittermeier, 2019; Figure S1.1 in Appendix S1 in Supporting Information) and we obtained 117,928 occurrences from the African Chiroptera Report database (Van Cakenberghe & Seamark, 2020). Two co‐authors (V.V.C. and E.S.) provided additional unpublished locations; the final occurrences database included 159,380 locations for 310 species. For 137 species with few (<20) or no occurrences, we looked for additional data from online databases (e.g., GBIF and iNaturalist), and peer‐reviewed literature (the complete list of data sources and references is given in Appendix S1 Table S1.2 and Table S1.3) and we obtained 3232 additional locations for 84 species. The full database included 162,612 locations for 310 species.

We removed all locations without coordinates, all duplicated records, and all records with dubious taxonomy. We excluded from further analyses 4 species for which no presence data were available as well as 133 species with less than 20 occurrences which would result in unstable and/or unrealistic models (van Proosdij et al., 2016; Wisz et al., 2008).

To limit autocorrelation in the data, for the remaining 177 species, we kept only one location per 1 km^2^ obtaining a total of 22,730 locations, with an average of 128 locations per species (range: 20–944).

For the entire set of 314 species, we also collected data on trophic guild (frugivores vs insectivores) and diet specialization (number of food items registered in the diet) from the Bat Eco‐Interactions database (Geiselman & Younger, 2020; http://batbase.org/) and Wilson and Mittermeier (2019). Food items were obtained at the family level for plants, and at the order level for arthropods. All species feeding on plant resources (fruits, nectar, other plant parts) were classified as frugivores while those feeding mainly on arthropods were classified as insectivores (Appendix S1 Table S1.4). Focusing on visitation, consumption, and predation of food resources, we defined as generalists all bat species feeding on more than one food item.

### 2.2 Environmental and biotic data

We included in our analyses as many variables as possible among those potentially important in shaping species distribution in bats (Cooper‐Bohannon et al., 2016; Herkt et al., 2016). We included abiotic variables (climate, terrain ruggedness, distance to waters), biotic variables (richness of trophic resources), and anthropogenic factors (human population density). All layers considered were resampled at 1 km^2^ resolution (Appendix S1 Table S1.5). All data management was performed in R 4.1.2 (packages *‘usdm’*, *‘fossil’*, ‘*randomForest*’) and ArcGis Pro 2.8.3 (ESRI ©).

All abiotic variables we included are linked directly or indirectly to the habitat used by bats. We included a Terrain Ruggedness Index (TRI) as a proxy for roost availability, assuming that more complex topographies are associated with greater availability of rock crevices (including cave‐like roosts; Kunz, 1982). We calculated the TRI following Nielsen et al. (2004) using a 90 m resolution digital elevation model (SRTM v4.1; Jarvis et al., 2008).

Inland water availability is a critically important factor for many bat species, especially in hot and dry areas such as those present in the African continent (Korine et al., 2016). Water springs, streams, rivers, ponds, and lakes are crucial not only for drinking but also for plant and invertebrate abundance (McCain, 2007; Monadjem et al., 2018). We included water availability in our analyses using two layers of distance to water: permanent water bodies and temporary water bodies. To define permanent inland waters, we used permanent lakes, ponds, rivers, and streams obtained combining the World Waterbodies database (ESRI ©), the World Waterlines database (ESRI ©) and the Global Surface Water database (GSW; Pekel et al., 2016). The two ESRI databases include vector layers while the GSW is a raster database with 30 m resolution. From the GSW, we considered the water transition layer with 10 water classes representing changes in water presence between any two consecutive years (from 1984 to 2015). We focused on permanent water (classes 1, 2, and 7). To define temporary inland waters, we considered the other 7 classes in the same GSW layer and temporary waterbodies, and ponds from the two ESRI databases. Furthermore, we included temporary waterbodies (3rd, 4th, and 5th Strahler order) from the AQUAMAPS Rivers of Africa database (FAO, 2014; https://data.apps.fao.org/aquamaps).

Climate variables (e.g., temperature and precipitation) are important predictors of habitat suitability for many bat species both directly given their physiological constraints (Jones et al., 2009; Ortega‐García et al., 2017) and indirectly considering the seasonal changes in the availability of their trophic resources (Cumming & Bernard, 1997). Therefore, we considered an initial set of 19 bioclimatic variables at 30 arc‐seconds resolution (roughly 1 km^2^ at the equator) obtained from Chelsa V2.1 (Karger et al., 2017).

We obtained human population density from the SEDAC database (Gao, 2020; https://sedac.ciesin.columbia.edu) which gives the number of people living in each 30 arc‐seconds resolution pixel. We considered this variable as a proxy for human disturbance represented by threats like habitat loss and roosts disturbance (e.g., for mining, cave tourism) as well as persecution and harvesting pressure for human–wildlife conflicts (e.g., orchards farmers) (Aziz et al., 2016; Mildenstein et al., 2016).

No information is available on trophic resource availability for bats at the scale of the entire African continent. Therefore, we included in the analyses a model of trophic resource richness as a proxy for biotic factors. From Cosentino and Maiorano (2021), we selected occurrences for plants (at the genus level) and arthropods (at the family level) consumed by African bats (Geiselman & Younger, 2020; Wilson & Mittermeier, 2019). We used a bias corrected version of these data to calibrate a Random Forest model (1000 trees; Breiman, 2001) for plants and for arthropods, using richness of taxa (Gotelli & Colwell, 2011) as response variables and climate (annual mean temperature, precipitation seasonality, temperature annual range, precipitation of warmest quarter, precipitation of coldest quarter), TRI, and water availability (km^2^ of water/ km^2^ pixel) as explanatory variables.

To exclude collinearity between predictors, we performed a variance inflation factor (VIF) analysis with all predictors, and we retained only variables with a VIF < 3 (Zuur et al., 2010; Appendix S2 Table S2.6). The final set of variables included climate (mean temperature of wettest quarter, mean temperature of driest quarter, precipitation of driest month, precipitation seasonality, precipitation of coldest quarter), distance to permanent and temporary waters, human population density, TRI, and trophic resources.

### 2.3 Species distribution models and statistical analysis

For each species, we calibrated two species distribution models, one (noBIO‐SDM) considering only abiotic variables (climate, TRI, distance to permanent and temporary waters, and human population density) and the other (BIO‐SDM) including all abiotic variables plus trophic resource availability. To calibrate the two SDMs with the same number of explanatory variables, we added to the noBIO‐SDM a dummy variable set to zero over entire Africa, therefore ensuring a full comparability between the two models (Zhang et al., 2022). We calibrated all models using a maximum entropy algorithm (Maxent v.3.4.1; Merow et al., 2013) with default parameters which produce reliable and accurate species distribution models (Valavi et al., 2022).

Although a target‐group approach is recommended to reduce the sampling bias when citizen science data are used (Ranc et al., 2017), our database on bat occurrences does not present any particular environmental bias (Appendix S2 Figure S2.7). Therefore, we considered for all species the same set of 10,000 random background points covering the entire African continent (Barbet‐Massin et al., 2012). We calibrated all models using a random 80% sample of the species occurrences and we used the remaining 20% for model evaluation and we repeated the same procedure for 10 replicates. For each replicate, we evaluated the predictive capacity of each model calculating the area under the curve (AUC) of the receiver operating characteristic (ROC; Swets, 1988), the true skill statistics (TSS; Allouche et al., 2006), and the Boyce index (Boyce et al., 2002). We evaluated the differences in predictive capacity between the BIO‐SDMs and noBIO‐SDMs performing a Wilcoxon test on the evaluation statistics (Wilcoxon, 1945). The final model was obtained by averaging all replicates with AUC > 0.7. For each replicate, we also measured variable importance using a jackknife approach which removes one variable at time and records the change in the AUC: the higher the change, the more important the variable (Peterson et al., 2011; Shcheglovitova & Anderson, 2013). To compare the variable importance between the two models, for each predictor variable, we calculated the average and standard deviation importance among all 177 bat species for BIO‐SDMs and noBIO‐SDMs. Using the same strategy, we also compared variable importance between the two models considering only generalist species.

For each species and for both the BIO‐SDM and the noBIO‐SDM, we mapped the potential species distribution in Africa. All final models were binarized using a species‐specific threshold maximizing the true skill statistics (TSS; Liu et al., 2016), a threshold that maximizes the ability of the model to discriminate presences from background points. We projected the binary models over all ecological zones (as defined by FAO, 2012; Appendix S2 Figure S2.8) by where the species occurs (Marsh et al., 2022; Monadjem et al., 2020; Wilson & Mittermeier, 2019), accounting therefore for historical biogeographical factors that were not possible to include in the modeling (e.g., dispersal limitations).

For each predictor variable, we also calculated the average response curves of frugivore and insectivore bats to evaluate the ecological reliability of our results compared with the available literature on African bats (Monadjem et al., 2020; Wilson & Mittermeier, 2019).

We mapped the spatial discrepancy among BIO‐SDMs and noBIO‐SDMs by measuring the percentage of species showing a difference between the two models in each pixel of the study area. We explored the influence of different traits on this discrepancy using a linear regression with a phylogenetic correction (Brownian model; Ho & Ané, 2014). We focused our analysis on four traits (number of occurrences, mean body mass, mean colony size, and the number of diet items) representing the detectability and trophic ecology of the species. Body mass and colony size were obtained from Wilson and Mittermeier (2019) and Monadjem et al. (2020), while phylogenetic data were downloaded from VertLife database (Upham et al., 2019). Since phylogenetic data were not available for all bats, we performed only this analysis on 162 species (out of 177).

## 3 RESULTS

All evaluation metrics gave comparable results with a slight yet significant (*p* < .0001) improvement of the predictive power for BIO‐SDMs compared to noBIO‐SDMs (Appendix S3 Table S3.9, Figure S3.10). Focusing on the AUC, BIO‐SDMs gave on average higher values (average = 0.94; st. dev. = 0.04; range: 0.77–0.99) compared to those of the noBIO‐SDMs (average AUC = 0.93; st.dev. = 0.04; range: 0.82–0.99).

Trophic resource availability was the most important variable in 62 species out of 177 with an average variable importance of almost 48% (Table 1; Appendix S3 Table S3.11). For 34 species, it was the second most important variable, while for 24 species, it was the third (Table 1; Appendix S3 Table S3.12, S3.13).

**TABLE 1:** Percentages of species for which each variable is ranked as the first, second, or third most important variable when trophic resource availability is included or excluded in SDM calibration.

Variable name	% species					
	Permutation importance rank with trophic resources			Permutation importance rank without trophic resources		
	**1st**	**2nd**	**3rd**	**1st**	**2nd**	**3rd**
Trophic resource availability	35.0%	19.2%	13.6%	/	/	/
Distance to Permanent Water	19.8%	20.9%	13.0%	39.6%	21.5%	10.2%
Distance to Temporary Water	0.6%	0%	0.6%	1.7%	2.8%	5.1%
Mean Temperature of Wettest Quarter (bio8)	2.8%	7.9%	4.0%	2.3%	7.3%	14.1%
Mean Temperature of Driest Quarter (bio9)	10.2%	9.6%	17.0%	8.5%	21.0%	16.4%
Precipitation of Driest Month (bio14)	18.6%	18.6%	11.9%	34.0%	16.4%	12.4%
Precipitation Seasonality (bio15)	5.7%	9.6%	11.3%	6.8%	11.9%	13.6%
Precipitation of Coldest Quarter (bio19)	2.8%	6.2%	12.4%	4.0%	11.9%	14.7%
Human Population Density	4.0%	7.9%	13.6%	2.8%	6.2%	11.3%
Terrain Ruggedness Index (TRI)	0.6%	0%	2.8%	0.6%	1.1%	2.3%

Climatic variables were important in shaping species distribution even when biotic variables are included, with “precipitation of the driest month” and “mean temperature of the driest quarter” being the first variable for 33 and 18 species, respectively (average importance of roughly 40% for both; Appendix S3 Table S3.14). For noBIO‐SDMs, the precipitation of the driest month was the most important variable in 60 species out of 177, with an average importance value of almost 50% (Appendix S3 Table S3.15).

Distance to inland permanent water was always a strong predictor (irrespective of the presence of the trophic resource availability), being the most important variable for 35 species in BIO‐SDMs (average importance = 36%; Table S3.14) and for 70 species in noBIO‐SDMs (average importance = 40%; Table S3.15). Distance to inland temporary waters, TRI, the other climatic variables, and human population density were often marginal in shaping species distribution for African bats.

**FIGURE 1:**
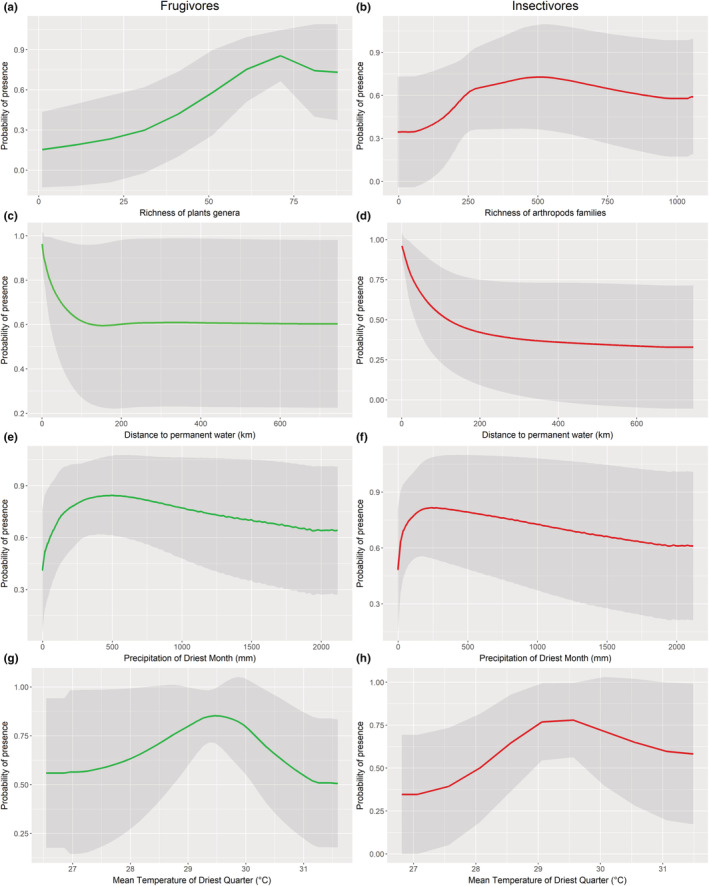
Response curves (average over all species/replicates) for models calibrated including the biotic variable. Shaded areas represent 1 standard deviation.

For both frugivore and insectivore bats, the response curves for the biotic variables showed an increasing probability of presence with increasing availability of trophic resources (Figure 1). Frugivore bats showed a constantly increasing probability of presence for higher plant richness, while insectivorous bats showed an increasing probability of presence up to medium arthropod richness, getting up to a plateau after which the probability of presence remained constant (Figure 1a, b).

The response curves for all other variables remained substantially unchanged for models with and without the trophic resources. All species showed a strong relationship with distance to inland permanent waters, decreasing their probability of presence for increasing distances to permanent waters (Figure 1c, d). The response curves of the climatic variables showed for all species an increasing probability of presence for increasing levels of precipitation, quickly reaching a peak in probability of presence at roughly 200–500 mm of precipitation in the driest month that gradually declines with increasing values (Figure 1e, f). The response curve for temperature was similar, reaching a peak at intermediate temperatures, and then decreasing (Figure 1g, h; see Appendix S3 Figure S3.16, S3.17 for response curves of other variables).

Among the species modeled, 70 were trophic generalists (Table S1.4); for 43% of these species, trophic resource availability was by far the most important variable (average importance = 47%; Appendix S3 Table S3.18, S3.19).

On average, BIO‐SDMs and noBIO‐SDMs differed for 7.8% of their spatial predictions (st.dev. = 5.1%; range: 0.3%–32.7%). For almost 22% of the species, the changes in potential distribution covered more than 10% of the study area (Appendix S3 Table S3.20). Species with a higher percentage of change showed a lower number of occurrences (*p* < .001), a high number of items in the diet (*p* < .001), and a small colony size (*p* < .05), while the body mass gave no significant result (Appendix S3 Table S3.21).

**FIGURE 2:**
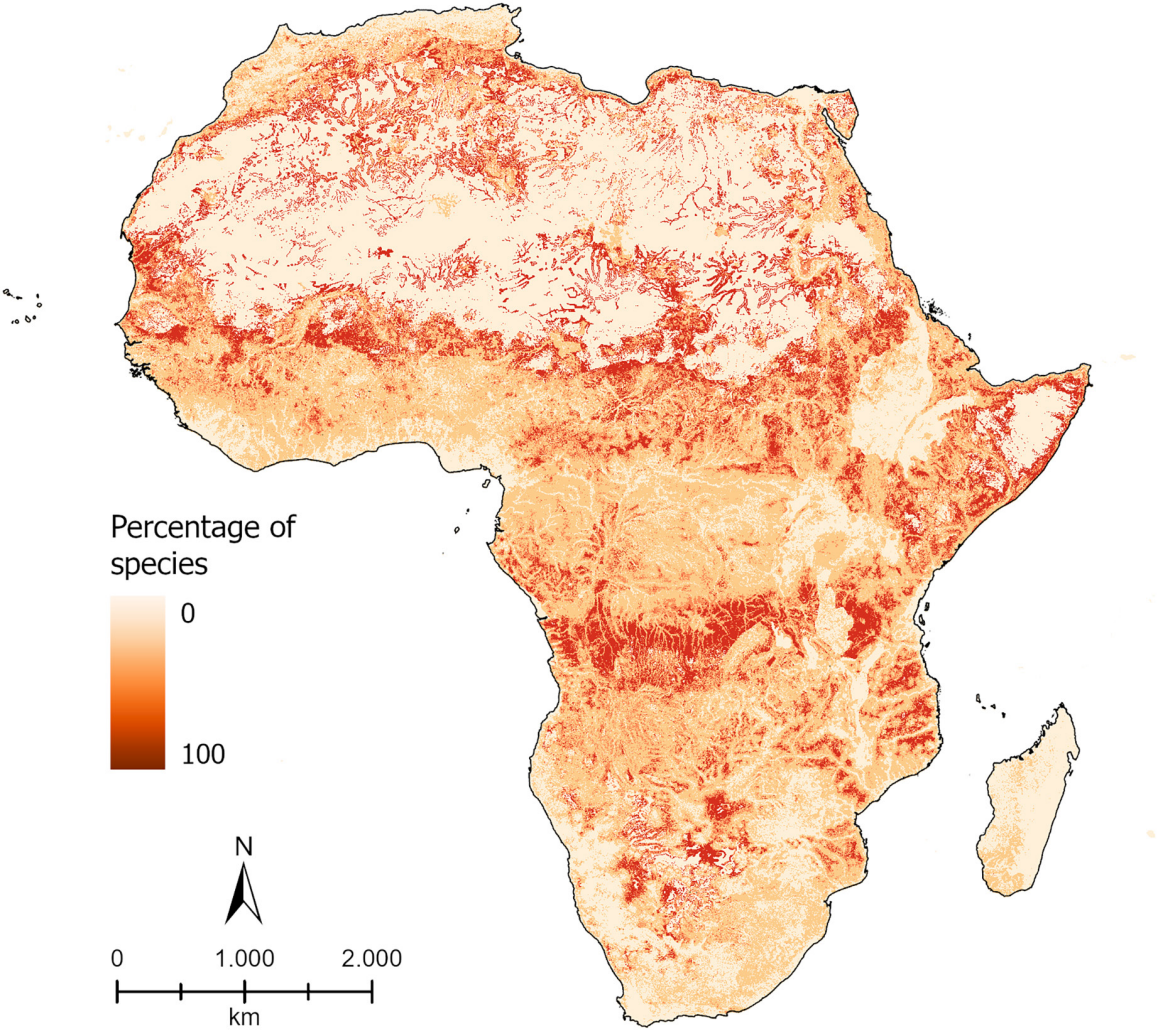
Percentage of species that showed a change in potential distribution when comparing models calibrated with and without trophic resources.

Tropical forests and shrublands host the areas with the highest percentage of species showing a change in their potential distribution between BIO‐SDMs and noBIO‐SDMs (Figure 2). More than 50% of the species showed a change in their potential distribution between the two models in the tropical moist forests surrounding the Congo basin, with peaks greater than 80% of the species along the Sahel belt (from Senegal up to Eritrea), and in Botswana. Also, the tropical rainforests in the south of the Congo basin showed a particularly high percentage of species with changes between the two models (>80%). Subtropical dry forests (Mediterranean coasts; South Africa), the Horn of Africa, the Namib desert, and most of Madagascar showed changes for <50% of the species in most of their area. In the Sahara desert, the BIO‐SDMs and noBIO‐SDMs were extremely similar, except in proximity of wadi (dry creeks and riverbeds) which showed changes in potential distribution for >80% of the species.

## 4 DISCUSSION

Traditionally, biogeographical analyses and species distribution models consider biotic factors at local scales only, while climatic variables dominate at regional to continental scales (Guisan & Thuiller, 2005; Pearson & Dawson, 2003; Wisz et al., 2013). However, the specific role of the different types of variables is still poorly covered in the existing literature, especially considering species with large niche breadth.

Previous studies considered only a limited number of species characterized by well‐known and direct biotic interactions, often focusing on the trophic niche, and on species with a high degree of diet specialization. The most common examples are related to host–plant or predator–prey interactions (Alaniz et al., 2020; Aragón & Sánchez‐Fernández, 2013; Araújo & Luoto, 2007). Few studies focused on trophic interactions considered prey richness to model the distribution of predators with a wider niche breadth, but still with well‐known trophic interactions (e.g., arctic fox‐Norway lemming, Hof et al., 2012; Gherghel et al., 2018). Here, modeling multiple species at the scale of the entire African continent, and using a very general proxy for biotic interactions in model calibration, we found that biotic factors do play an important role when modeling species distribution at macro scales irrespective of the ecology of the species considered. In fact, trophic resources were in the top three ranks of importance for most species (roughly 68%), including generalist species with a wider niche breadth, for which the availability of trophic resources should not be a limiting factor. Although the importance of considering other factors besides climate in SDMs is now confirmed by several studies, this idea has been tested so far at macro scales only focusing on species highly dependent on the biotic factor considered. Our findings highlight the importance of considering species interactions in SDMs at macro scales regardless of the species dependence for the biotic factor considered.

Moreover, the inclusion of biotic interactions in predicting species distribution affected the species' spatial predictions. In fact, the areas with the highest richness of bats (Herkt et al., 2016) showed a change in potential distribution between BIO‐SDMs and noBIO‐SDMs for more than 80% of the species. In particular, the highest percentages are in areas highly variable both from an environmental and climatic point of view (e.g., Sahel belt, wadi in the Sahara desert). These results highlight the importance of including biotic interactions in modeling species distributions in specific areas and hotspots of biodiversity with potential conservation and management implications. Along the same line, we found the highest percentages of change in rare species, with limited colony size, and a high number of food items in the diet, highlighting the significant role of biotic factors in the explicative part of the model, especially for species hard to detect for which the climatic niche may be undersampled, and more mechanistic factors are needed.

The interpretation of our results should clearly consider the limitations and assumptions of our analyses. First of all, we considered only one type of biotic interaction among all those possible (e.g., competition, mutualism, parasitism; Morales‐Castilla et al., 2015). Although trophic interaction represents a fundamental component of the realized niche of a species (Hutchinson, 1957), competitive interactions, parasitism and diseases, facilitations and others could be important as well (Araújo & Guisan, 2006; Mpakairi et al., 2017).

Second, we used a generic proxy of the trophic interaction (trophic resource richness) based on high taxonomic level of food resources which is not necessarily representative of the trophic niche of a species. Valuable alternatives are represented by abundance or biomass of prey species, and species‐specific trophic links or food‐web data with approaches such as joint species distribution models (Pollock et al., 2014) and food‐web analysis (e.g., Braga et al., 2019; Gaüzère et al., 2022). However, these approaches are almost impossible at the continental scale and for organisms for which species‐specific data on food preferences are often not available, especially in remote areas such as the African continent. On the other hand, given the paucity of data on species‐specific trophic interactions, relying on high taxonomic levels (e.g., arthropods order) is a common approach at macro scales (e.g., insect orders/families in Boyles & Storm, 2007, Dodd et al., 2012; plants genera in Sánchez & Giannini, 2018), as well as using proxies for biotic interactions (Alaniz et al., 2020; Aragón & Sánchez‐Fernández, 2013; de Araújo et al., 2014; Gherghel et al., 2018). Moreover, experimental evidence showed that the relationship between species richness and productivity/biomass is almost always positive or hump‐shaped in both plants and animals at several geographic scales (Liang et al., 2016; Mittelbach et al., 2001; Ouyang et al., 2019). Therefore, we expect that areas supporting a high diversity of species are likely also to harbor a high density of individuals, providing a good representation of trophic resource availability.

Finally, even if a few variables were in common between the trophic resource model and the bat SDMs (precipitation seasonality, precipitation of coldest quarter, TRI index), we found no sign of collinearity among our predictors. We performed a classical VIF analysis which, however, is able to detect only linear relationships (Table S2.6). We also checked the stability in variable importance and in the shape of their response curves. Both would be influenced by collinearity, but they remained unchanged when the biotic variable is excluded from the model (Table S3.14, S3.15, Figure S3.16, S3.17), clearly indicating the absence of problems.

Despite the potential uncertainties of our study, we provide evidence of the importance of including biotic interactions in SDMs at macro scales. Trophic resource richness was particularly important for generalist species confirming also our second hypothesis. In this framework, proxies for trophic interactions like species richness have been proven to be useful in SDMs when species‐specific data are not available. Nevertheless, despite the improvements that have been made in the modeling frameworks to investigate this question, bridging the state of knowledge on species interactions remains a fundamental and urgent challenge, particularly in the regions with the highest level of biodiversity (Hortal et al., 2015).

Future directions should explore multi‐scale analysis considering that both abiotic and biotic factors change over time and space. Studies that investigate biotic interactions through time and space combined with a deeper knowledge of the species are crucial, especially in a global change context.

## ACKNOWLEDGEMENTS

We thank Luke Sutton and two anonymous referees for providing insightful and detailed comments which improved an earlier version of the manuscript. The publication fees were kindly provided by the Ph.D. in Environmental and Evolutionary Biology (Sapienza University of Rome).

## AUTHOR CONTRIBUTIONS

**Francesca Cosentino:** Conceptualization (lead); data curation (lead); formal analysis (lead); methodology (lead); writing – original draft (lead); writing – review and editing (equal). **Ernest Charles James Seamark:** Resources (supporting); supervision (supporting); writing – review and editing (equal). **Victor Van Cakenberghe:** Resources (supporting); supervision (supporting); writing – review and editing (equal). **Luigi Maiorano:** Conceptualization (lead); formal analysis (supporting); methodology (lead); supervision (lead); writing – review and editing (equal).

## Supporting information


**Data S1:** Supporting Information

## Data Availability

The data used in the analysis are all publicly available and their references and sources are given in the manuscript and in Appendix S1 in Supporting Information.
