# Supplementary material for: Not only climate: The importance of biotic interactions in shaping species distributions at macro scales
Source: Ecol Evol. 2023 Mar 20;13(3):e9855. doi: 10.1002/ece3.9855 (PMC10027549; doi:10.1002/ece3.9855)
Supplement: Supplementary file 1 — Data S1: Supporting Information [file ECE3-13-e9855-s001.docx]

**SUPPORTING INFORMATION**

Not only climate: the importance of biotic interactions in shaping species distributions at macro-scales

Cosentino F.^1^*, Seamark E. C. J.^2^, Van Cakenberghe V.^2,3^, Maiorano L. ^1^

^1.^ Department of Biology and Biotechnologies “Charles Darwin”, Sapienza University of Rome

^2.^ AfricanBats NPC, 357 Botha Ave, Kloofsig, 0157, Republic of South Africa

^3.^ FunMorph Lab, Department of Biology, University of Antwerp, Belgium

*Corresponding author: [francesca.cosentino@uniroma1.it](mailto:francesca.cosentino@uniroma1.it)

**SUPPORTING INFORMATION CAPTIONS**

APPENDIX S1

- Figure S1.1. Map of Africa with national boundaries.
- Table S1.2. Complete list of sources used in bats occurrence data collection.
- Table S1.3. List of citations of GBIF downloaded data.
- Table S1.4. List of African bats considered with their trophic guild, number of items in the diet, and number of occurrences in Africa. In ‘Trophic guild’: F= frugivores, I= insectivores; in ‘N items in diet’: number of plant families or orders of arthropods consumed (0= no information on the number of items consumed).
- Table S1.5. Set of variables considered for the analysis.

APPENDIX S2

- Table S2.6. Results of Variance Inflation Factor (VIF) for frugivore (A) and insectivore (B) bats.
- Figure S2.7. Boxplots of explanatory variables in random versus target-group selected background. a) Plants richness; b) Arthropods richness; c) Mean Temperature of Wettest Quarter; d) Mean Temperature of Driest Quarter; e) Precipitation of Driest Month; f) Precipitation Seasonality; g) Precipitation of Coldest Quarter; h) Terrain Ruggedness Index; i) Distance to Permanent Water; j) Distance to Temporary Water; k) Human Population Density.
- Figure S2.8. FAO Global Ecological Zones (FAO, 2012) classified in 15 biogeographically homogeneous areas for the present study.

APPENDIX S3

- Table S3.9. Species-specific average and standard deviation AUC, TSS, and Boyce index calculated over 10 replicates for models calibrated with (“_BIO”) and without the trophic resource variable (“_noBIO”).
- Figure S3.10. AUC, TSS, and Boyce indicators for models calibrated with (“BIO-SDMs”, red) and without the trophic resource variable (“noBIO-SDMs”, blue) for frugivore (left) and insectivore (right) bats.
- Table S3.11. Average and standard deviation permutation importance calculated over 62 species out of 177 for which the trophic resource variable is at the first rank of importance.
- Table S3.12. Average and standard deviation permutation importance calculated over 34 species out of 177 for which the trophic resource variable is at the second rank of importance.
- Table S3.13. Average and standard deviation permutation importance calculated over 24 species out of 177 for which the trophic resource variable is at the third rank of importance.
- Table S3.14. Percentage of species, average and standard deviation permutation importance when variables are at the first rank of importance over all 177 species in BIO-SDMs.
- Table S3.15. Percentage of species, average and standard deviation permutation importance when variables are at the first rank of importance over all 177 species in noBIO-SDMs.
- Figure S3.16. Response curves (average over all species/replicates) for models calibrated including the biotic variable (BIO-SDMs) for frugivore (left) and insectivore (right) bats. Shaded areas represent 1 standard deviation. a) and b) Mean Temperature of Wettest Quarter; c) and d) Precipitation Seasonality; e) and f) Precipitation of Coldest Quarter; g) and h) Distance to temporary water; i) and j) Terrain Ruggedness Index; k) and l) Human Population Density.
- Figure S3.17. Response curves (average over all species/replicates) for models calibrated including only abiotic variables (noBIO-SDMs) for frugivores (left) and insectivores (right) bats. Shaded areas represent 1 standard deviation. a) and b) Mean Temperature of Wettest Quarter; c) and d) Mean Temperature of Driest Quarter; e) and f) Precipitation of Driest Month; g) and h) Precipitation Seasonality; Distance to temporary water; i) and j) Precipitation of Coldest Quarter; k) and l) Distance to permanent water; m) and n) Distance to temporary water; o) and p) Terrain Ruggedness Index; q) and r) Human Population Density.
- Table S3.18. Average and standard deviation permutation importance of generalist species when the trophic resource is ranked as the first (43% of species) most important variable.
- Table S3.19. Average and standard deviation permutation importance calculated over 70 generalist species.
- Table S3.20. Species-specific percentage of change in potential distribution between BIO-SDMs and noBIO-SDMs.
- Table S3.21. Results of linear regression with a phylogenetic correction. Signif. codes: 0.0001 ‘***’ 0.001 ‘**’ 0.01 ‘*’ 0.05 ‘.’ 0.1 ‘n.s.’ 1.

**APPENDIX S1**

Figure S1.1. Map of Africa with national boundaries.


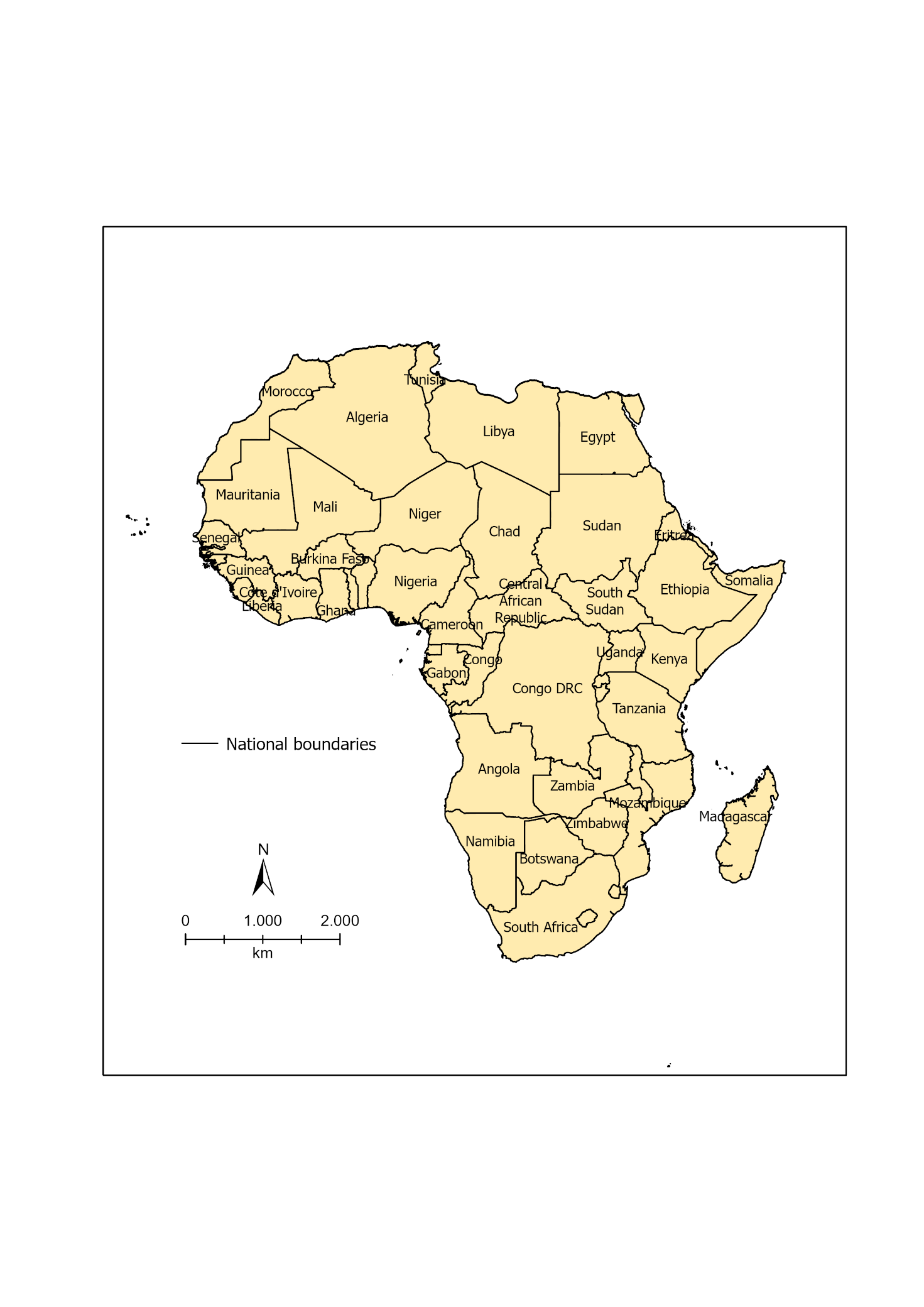


Table S1.2. Complete list of sources used in bats occurrence data collection.

| Source | Type |
| --- | --- |
| Van Cakenberghe, V., & Seamark, E.C.J. (Eds.), 2020. ACR 2020. African Chiroptera Report 2020. AfricanBats NPC, Pretoria. i-xviii + 8542 pp. | Literature |
| Monadjem, A., Taylor, P. J., & Schoeman, M. C., 2020. Bats of southern and central Africa: a biogeographic and taxonomic synthesis. Wits University Press. | Literature |
| Wilson, D.E. & Mittermeier, R.A. (Eds.), 2019. Handbook of the Mammals of the World. Vol. 9. Bats. Lynx Edicions, Barcelona. | Literature |
| Happold, M. & Happold, D. C. D. (Eds.), 2013. Mammals of Africa. Volume IV: Hedgehogs, Shrews and Bats. Bloomsbury Publishing, London. | Literature |
| IUCN 2021. The IUCN Red List of Threatened Species. Version 2021-3. <https://www.iucnredlist.org>. Accessed on October 2021 | Literature |
| Global Biodiversity Information Facility; GBIF; <http://www.gbif.org/>. Accessed on October 2021 | Citizen science/Museum records |
| iNaturalist, available from <https://www.inaturalist.org>. Accessed on October 2021 | Citizen science |
| Arctos Database, available from <http://arctos.database.museum/>. Accessed on October 2021 | Museum records |

Table S1.3. List of citations of GBIF downloaded data.

| Species | Citation |
| --- | --- |
| *Casinycteris campomaanensis* | GBIF.org (01 October 2021) GBIF Occurrence Download  <https://doi.org/10.15468/dl.gm2rvk> |
| *Chaerephon aloysiisabaudiae* | GBIF.org (21 October 2021) GBIF Occurrence Download <https://doi.org/10.15468/dl.hpqwbg> |
| *Chaerephon bemmeleni* | GBIF.org (25 October 2021) GBIF Occurrence Download <https://doi.org/10.15468/dl.5bnm8u> |
| *Chaerephon jobimena* | GBIF.org (01 October 2021) GBIF Occurrence Download <https://doi.org/10.15468/dl.yypma4> |
| *Chaerephon russatus* | GBIF.org (21 October 2021) GBIF Occurrence Download <https://doi.org/10.15468/dl.k32k2n> |
| *Coleura kibomalandy* | GBIF.org (20 October 2021) GBIF Occurrence Download <https://doi.org/10.15468/dl.dyq8b6> |
| *Doryrhina camerunensis* | GBIF.org (21 October 2021) GBIF Occurrence Download <https://doi.org/10.15468/dl.yn7v2a> |
| *Epomophorus anselli* | GBIF.org (20 October 2021) GBIF Occurrence Download <https://doi.org/10.15468/dl.mk46w9> |
| *Glauconycteris alboguttata* | GBIF.org (21 October 2021) GBIF Occurrence Download <https://doi.org/10.15468/dl.93q3tt> |
| *Glauconycteris curryae* | GBIF.org (21 October 2021) GBIF Occurrence Download <https://doi.org/10.15468/dl.z9cftw> |
| *Glauconycteris egeria* | GBIF.org (21 October 2021) GBIF Occurrence Download <https://doi.org/10.15468/dl.wayeyv> |
| *Glauconycteris gleni* | GBIF.org (09 October 2021) GBIF Occurrence Download <https://doi.org/10.15468/dl.n6dg8h> |
| *Glauconycteris kenyacola* | GBIF.org (01 October 2021) GBIF Occurrence Download <https://doi.org/10.15468/dl.ewwddn> |
| *Glauconycteris machadoi* | GBIF.org (01 October 2021) GBIF Occurrence Download <https://doi.org/10.15468/dl.dh9a73> |
| *Glauconycteris superba* | GBIF.org (21 October 2021) GBIF Occurrence Download <https://doi.org/10.15468/dl.2ht9gc> |
| *Hipposideros curtus* | GBIF.org (21 October 2021) GBIF Occurrence Download <https://doi.org/10.15468/dl.8nhup4> |
| *Hipposideros lamottei* | GBIF.org (21 October 2021) GBIF Occurrence Download <https://doi.org/10.15468/dl.3vhwy8> |
| *Hipposideros marisae* | GBIF.org (20 October 2021) GBIF Occurrence Download <https://doi.org/10.15468/dl.rwmmws> |
| *Hipposideros megalotis* | GBIF.org (29 November 2021) GBIF Occurrence Download <https://doi.org/10.15468/dl.7yeqxb> |
| *Hypsugo ariel* | GBIF.org (20 October 2021) GBIF Occurrence Download <https://doi.org/10.15468/dl.ab55nc> |
| *Hypsugo bemainty* | GBIF.org (20 October 2021) GBIF Occurrence Download <https://doi.org/10.15468/dl.zgdxq5> |
| *Hypsugo eisentrauti* | GBIF.org (25 October 2021) GBIF Occurrence Download <https://doi.org/10.15468/dl.h5q2gc> |
| *Hypsugo musciculus* | GBIF.org (21 October 2021) GBIF Occurrence Download <https://doi.org/10.15468/dl.pdvzhb> |
| *Hypsugo savii* | GBIF.org (25 October 2021) GBIF Occurrence Download <https://doi.org/10.15468/dl.wbseau> |
| *Kerivoula cuprosa* | GBIF.org (20 October 2021) GBIF Occurrence Download <https://doi.org/10.15468/dl.qmua6v> |
| *Kerivoula phalaena* | GBIF.org (21 October 2021) GBIF Occurrence Download <https://doi.org/10.15468/dl.eedkbb> |
| *Laephotis angolensis* | GBIF.org (21 October 2021) GBIF Occurrence Download <https://doi.org/10.15468/dl.czhmm5> |
| *Laephotis namibensis* | GBIF.org (21 October 2021) GBIF Occurrence Download <https://doi.org/10.15468/dl.mpywrh> |
| *Laephotis wintoni* | GBIF.org (21 October 2021) GBIF Occurrence Download <https://doi.org/10.15468/dl.2br2ep> |
| *Miniopterus ambohitrensis* | GBIF.org (21 October 2021) GBIF Occurrence Download <https://doi.org/10.15468/dl.y2kt6c> |
| *Miniopterus brachytragos* | GBIF.org (21 October 2021) GBIF Occurrence Download <https://doi.org/10.15468/dl.gdmqpn> |
| *Miniopterus egeri* | GBIF.org (21 October 2021) GBIF Occurrence Download <https://doi.org/10.15468/dl.3n4kua> |
| *Miniopterus gleni* | GBIF.org (25 October 2021) GBIF Occurrence Download <https://doi.org/10.15468/dl.k4a6ab> |
| *Miniopterus griffithsi* | GBIF.org (20 October 2021) GBIF Occurrence Download <https://doi.org/10.15468/dl.rskg4k> |
| *Miniopterus sororculus* | GBIF.org (25 October 2021) GBIF Occurrence Download <https://doi.org/10.15468/dl.nh54j2> |
| *Mops bakarii* | GBIF.org (01 October 2021) GBIF Occurrence Download <https://doi.org/10.15468/dl.g4r9fc> |
| *Mops congicus* | GBIF.org (25 October 2021) GBIF Occurrence Download <https://doi.org/10.15468/dl.qvjqdm> |
| *Mops petersoni* | GBIF.org (21 October 2021) GBIF Occurrence Download <https://doi.org/10.15468/dl.7j6m4x> |
| *Mops trevori* | GBIF.org (21 October 2021) GBIF Occurrence Download <https://doi.org/10.15468/dl.urfwkm> |
| *Mormopterus acetabulosus* | GBIF.org (25 October 2021) GBIF Occurrence Download <https://doi.org/10.15468/dl.y79e5u> |
| *Mormopterus francoismoutoui* | GBIF.org (25 October 2021) GBIF Occurrence Download <https://doi.org/10.15468/dl.vwjxs9> |
| *Myonycteris relicta* | GBIF.org (25 October 2021) GBIF Occurrence Download <https://doi.org/10.15468/dl.69wm3h> |
| *Myopterus daubentonii* | GBIF.org (21 October 2021) GBIF Occurrence Download <https://doi.org/10.15468/dl.c8cvpe> |
| *Myotis anjouanensis* | GBIF.org (20 October 2021) GBIF Occurrence Download <https://doi.org/10.15468/dl.3dwfaq> |
| *Myotis emarginatus* | GBIF.org (21 October 2021) GBIF Occurrence Download <https://doi.org/10.15468/dl.h32cc3> |
| *Myzopoda aurita* | GBIF.org (25 October 2021) GBIF Occurrence Download <https://doi.org/10.15468/dl.25n98k> |
| *Myzopoda schliemanni* | GBIF.org (20 October 2021) GBIF Occurrence Download <https://doi.org/10.15468/dl.mwqhhs> |
| *Neoromicia malagasyensis* | GBIF.org (20 October 2021) GBIF Occurrence Download <https://doi.org/10.15468/dl.gaxc4d> |
| *Nycteris madagascariensis* | GBIF.org (20 October 2021) GBIF Occurrence Download <https://doi.org/10.15468/dl.5kg5k4> |
| *Nycteris major* | GBIF.org (21 October 2021) GBIF Occurrence Download <https://doi.org/10.15468/dl.vrrg57> |
| *Nycteris parisii* | GBIF.org (21 October 2021) GBIF Occurrence Download <https://doi.org/10.15468/dl.w93g9x> |
| *Otomops madagascariensis* | GBIF.org (25 October 2021) GBIF Occurrence Download <https://doi.org/10.15468/dl.fkxgdx> |
| *Paratriaenops auritus* | GBIF.org (01 October 2021) GBIF Occurrence Download <https://doi.org/10.15468/dl.nm3tyu> |
| *Paratriaenops furcula* | GBIF.org (21 October 2021) GBIF Occurrence Download <https://doi.org/10.15468/dl.48ghmt> |
| *Paremballonura tiavato* | GBIF.org (25 October 2021) GBIF Occurrence Download <https://doi.org/10.15468/dl.6uwfee> |
| *Pipistrellus aero* | GBIF.org (20 October 2021) GBIF Occurrence Download <https://doi.org/10.15468/dl.9ume73> |
| *Pipistrellus inexspectatus* | GBIF.org (21 October 2021) GBIF Occurrence Download <https://doi.org/10.15468/dl.ugqdrg> |
| *Pipistrellus raceyi* | GBIF.org (20 October 2021) GBIF Occurrence Download <https://doi.org/10.15468/dl.evqe4w> |
| *Platymops setiger* | GBIF.org (21 October 2021) GBIF Occurrence Download <https://doi.org/10.15468/dl.mk2rxu> |
| *Plerotes anchietae* | GBIF.org (21 October 2021) GBIF Occurrence Download <https://doi.org/10.15468/dl.w667zs> |
| *Pteropus livingstonii* | GBIF.org (20 October 2021) GBIF Occurrence Download <https://doi.org/10.15468/dl.xbkfqb> |
| *Pteropus rodricensis* | GBIF.org (21 October 2021) GBIF Occurrence Download <https://doi.org/10.15468/dl.3s2uk6> |
| *Pteropus voeltzkowi* | GBIF.org (25 October 2021) GBIF Occurrence Download <https://doi.org/10.15468/dl.xsnk8p> |
| *Rhinolophus alticolus* | GBIF.org (27 October 2021) GBIF Occurrence Download <https://doi.org/10.15468/dl.9969ke> |
| *Rhinolophus guineensis* | GBIF.org (25 October 2021) GBIF Occurrence Download <https://doi.org/10.15468/dl.mbe3ce> |
| *Rhinolophus hillorum* | GBIF.org (21 October 2021) GBIF Occurrence Download <https://doi.org/10.15468/dl.srkrek> |
| *Rhinolophus maclaudi* | GBIF.org (29 November 2021) GBIF Occurrence Download <https://doi.org/10.15468/dl.tyjvub> |
| *Rhinolophus maendeleo* | GBIF.org (21 October 2021) GBIF Occurrence Download <https://doi.org/10.15468/dl.dcjvxv> |
| *Rhinolophus mossambicus* | GBIF.org (21 October 2021) GBIF Occurrence Download <https://doi.org/10.15468/dl.9gpedf> |
| *Rhinolophus ruwenzorii* | GBIF.org (25 October 2021) GBIF Occurrence Download <https://doi.org/10.15468/dl.b8w69s> |
| *Rhinolophus sakejiensis* | GBIF.org (01 October 2021) GBIF Occurrence Download <https://doi.org/10.15468/dl.pn7bg4> |
| *Rhinolophus willardi* | GBIF.org (20 October 2021) GBIF Occurrence Download <https://doi.org/10.15468/dl.gzjkwz> |
| *Scotophilus altilis* | GBIF.org (21 October 2021) GBIF Occurrence Download <https://doi.org/10.15468/dl.ee6y3a> |
| *Scotophilus andrewreborii* | GBIF.org (21 October 2021) GBIF Occurrence Download <https://doi.org/10.15468/dl.bet44f> |
| *Scotophilus borbonicus* | GBIF.org (01 October 2021) GBIF Occurrence Download <https://doi.org/10.15468/dl.jftps8> |
| *Scotophilus colias* | GBIF.org (27 October 2021) GBIF Occurrence Download <https://doi.org/10.15468/dl.rbqn9d> |
| *Scotophilus livingstonii* | GBIF.org (20 October 2021) GBIF Occurrence Download <https://doi.org/10.15468/dl.ux62ny> |
| *Scotophilus marovaza* | GBIF.org (20 October 2021) GBIF Occurrence Download <https://doi.org/10.15468/dl.envaae> |
| *Scotophilus nucella* | GBIF.org (21 October 2021) GBIF Occurrence Download <https://doi.org/10.15468/dl.gspcu7> |
| *Scotophilus robustus* | GBIF.org (20 October 2021) GBIF Occurrence Download <https://doi.org/10.15468/dl.x7anzd> |
| *Scotophilus trujilloi* | GBIF.org (20 October 2021) GBIF Occurrence Download <https://doi.org/10.15468/dl.g9jnmw> |
| *Tadarida lobata* | GBIF.org (25 October 2021) GBIF Occurrence Download <https://doi.org/10.15468/dl.c97gta> |
| *Taphozous hamiltoni* | GBIF.org (21 October 2021) GBIF Occurrence Download <https://doi.org/10.15468/dl.fumqaw> |
| *Triaenops menamena* | GBIF.org (25 October 2021) GBIF Occurrence Download <https://doi.org/10.15468/dl.e7n3hh> |

Table S1.4. List of African bats considered with their trophic guild, number of items in the diet, and number of occurrences in Africa. In ‘Trophic guild’: F= frugivore, I= insectivore; in ‘N items in diet’: number of plant families or orders of arthropods consumed (0= no information on the number of families/orders consumed).

| Family | Species | Trophic guild | N items in diet | N occurrences in Africa |
| --- | --- | --- | --- | --- |
| Hipposideridae | *Asellia italosomalica* | I | 0 | 0 |
| Hipposideridae | *Asellia patrizii* | I | 0 | 2 |
| Hipposideridae | *Asellia tridens* | I | 4 | 74 |
| Vespertilionidae | *Barbastella barbastellus* | I | 1 | 3 |
| Vespertilionidae | *Barbastella leucomelas* | I | 2 | 3 |
| Megadermatidae | *Cardioderma cor* | I | 4 | 84 |
| Pteropodidae | *Casinycteris argynnis* | F | 1 | 31 |
| Pteropodidae | *Casinycteris campomaanensis* | F | 0 | 2 |
| Pteropodidae | *Casinycteris ophiodon* | F | 1 | 11 |
| Molossidae | *Chaerephon aloysiisabaudiae* | I | 0 | 20 |
| Molossidae | *Chaerephon ansorgei* | I | 3 | 72 |
| Molossidae | *Chaerephon atsinanana* | I | 2 | 25 |
| Molossidae | *Chaerephon bemmeleni* | I | 0 | 18 |
| Molossidae | *Chaerephon bivittatus* | I | 0 | 54 |
| Molossidae | *Chaerephon chapini* | I | 1 | 25 |
| Molossidae | *Chaerephon gallagheri* | I | 0 | 0 |
| Molossidae | *Chaerephon jobimena* | I | 0 | 7 |
| Molossidae | *Chaerephon leucogaster* | I | 0 | 65 |
| Molossidae | *Chaerephon major* | I | 0 | 46 |
| Molossidae | *Chaerephon nigeriae* | I | 1 | 91 |
| Molossidae | *Chaerephon pumilus* | I | 5 | 825 |
| Molossidae | *Chaerephon pusillus* | I | 0 | 33 |
| Molossidae | *Chaerephon russatus* | I | 0 | 18 |
| Molossidae | *Chaerephon tomensis* | I | 0 | 2 |
| Cistugidae | *Cistugo lesueuri* | I | 2 | 24 |
| Cistugidae | *Cistugo seabrae* | I | 4 | 17 |
| Rhinonycteridae | *Cloeotis percivali* | I | 1 | 47 |
| Emballonuridae | *Coleura afra* | I | 2 | 95 |
| Emballonuridae | *Coleura kibomalandy* | I | 0 | 5 |
| Emballonuridae | *Coleura seychellensis* | I | 2 | 7 |
| Hipposideridae | *Doryrhina camerunensis* | I | 0 | 17 |
| Hipposideridae | *Doryrhina cyclops* | I | 1 | 161 |
| Pteropodidae | *Eidolon dupreanum* | F | 10 | 25 |
| Pteropodidae | *Eidolon helvum* | F | 21 | 402 |
| Pteropodidae | *Epomophorus angolensis* | F | 0 | 31 |
| Pteropodidae | *Epomophorus anselli* | F | 0 | 5 |
| Pteropodidae | *Epomophorus crypturus* | F | 10 | 243 |
| Pteropodidae | *Epomophorus dobsonii* | F | 0 | 49 |
| Pteropodidae | *Epomophorus gambianus* | F | 4 | 289 |
| Pteropodidae | *Epomophorus grandis* | F | 0 | 2 |
| Pteropodidae | *Epomophorus intermedius* | F | 0 | 4 |
| Pteropodidae | *Epomophorus labiatus* | F | 5 | 397 |
| Pteropodidae | *Epomophorus minor* | F | 0 | 121 |
| Pteropodidae | *Epomophorus pusillus* | F | 7 | 391 |
| Pteropodidae | *Epomophorus wahlbergi* | F | 1 | 545 |
| Pteropodidae | *Epomops buettikoferi* | F | 5 | 72 |
| Pteropodidae | *Epomops franqueti* | F | 7 | 432 |
| Vespertilionidae | *Eptesicus bottae* | I | 4 | 2 |
| Vespertilionidae | *Eptesicus floweri* | I | 0 | 7 |
| Vespertilionidae | *Eptesicus hottentotus* | I | 1 | 81 |
| Vespertilionidae | *Eptesicus isabellinus* | I | 4 | 26 |
| Vespertilionidae | *Eptesicus platyops* | I | 0 | 1 |
| Vespertilionidae | *Glauconycteris alboguttata* | I | 0 | 14 |
| Vespertilionidae | *Glauconycteris argentata* | I | 0 | 78 |
| Vespertilionidae | *Glauconycteris atra* | I | 0 | 3 |
| Vespertilionidae | *Glauconycteris beatrix* | I | 0 | 27 |
| Vespertilionidae | *Glauconycteris curryae* | I | 0 | 17 |
| Vespertilionidae | *Glauconycteris egeria* | I | 0 | 13 |
| Vespertilionidae | *Glauconycteris gleni* | I | 0 | 5 |
| Vespertilionidae | *Glauconycteris humeralis* | I | 0 | 15 |
| Vespertilionidae | *Glauconycteris kenyacola* | I | 0 | 3 |
| Vespertilionidae | *Glauconycteris machadoi* | I | 0 | 2 |
| Vespertilionidae | *Glauconycteris poensis* | I | 0 | 44 |
| Vespertilionidae | *Glauconycteris superba* | I | 0 | 9 |
| Vespertilionidae | *Glauconycteris variegata* | I | 1 | 100 |
| Hipposideridae | *Hipposideros abae* | I | 0 | 38 |
| Hipposideridae | *Hipposideros beatus* | I | 0 | 86 |
| Hipposideridae | *Hipposideros caffer* | I | 1 | 643 |
| Hipposideridae | *Hipposideros curtus* | I | 0 | 14 |
| Hipposideridae | *Hipposideros fuliginosus* | I | 0 | 39 |
| Hipposideridae | *Hipposideros jonesi* | I | 0 | 24 |
| Hipposideridae | *Hipposideros lamottei* | I | 0 | 10 |
| Hipposideridae | *Hipposideros marisae* | I | 0 | 4 |
| Hipposideridae | *Hipposideros megalotis* | I | 0 | 11 |
| Hipposideridae | *Hipposideros ruber* | I | 1 | 494 |
| Hipposideridae | *Hipposideros tephrus* | I | 0 | 63 |
| Pteropodidae | *Hypsignathus monstrosus* | F | 10 | 200 |
| Vespertilionidae | *Hypsugo anchieta* | I | 0 | 62 |
| Vespertilionidae | *Hypsugo ariel* | I | 4 | 6 |
| Vespertilionidae | *Hypsugo bemainty* | I | 1 | 7 |
| Vespertilionidae | *Hypsugo crassulus* | I | 0 | 28 |
| Vespertilionidae | *Hypsugo eisentrauti* | I | 0 | 16 |
| Vespertilionidae | *Hypsugo musciculus* | I | 0 | 11 |
| Vespertilionidae | *Hypsugo savii* | I | 3 | 16 |
| Vespertilionidae | *Kerivoula africana* | I | 0 | 4 |
| Vespertilionidae | *Kerivoula argentata* | I | 0 | 51 |
| Vespertilionidae | *Kerivoula cuprosa* | I | 0 | 8 |
| Vespertilionidae | *Kerivoula eriophora* | I | 0 | 0 |
| Vespertilionidae | *Kerivoula lanosa* | I | 0 | 53 |
| Vespertilionidae | *Kerivoula phalaena* | I | 0 | 18 |
| Vespertilionidae | *Kerivoula smithii* | I | 0 | 17 |
| Vespertilionidae | *Laephotis angolensis* | I | 0 | 8 |
| Vespertilionidae | *Laephotis botswanae* | I | 3 | 37 |
| Vespertilionidae | *Laephotis namibensis* | I | 2 | 7 |
| Vespertilionidae | *Laephotis wintoni* | I | 2 | 21 |
| Megadermatidae | *Lavia frons* | I | 4 | 284 |
| Pteropodidae | *Lissonycteris angolensis* | F | 7 | 297 |
| Hipposideridae | *Macronycteris commersonii* | I | 1 | 75 |
| Hipposideridae | *Macronycteris cryptovalorona* | I | 0 | 2 |
| Hipposideridae | *Macronycteris gigas* | I | 2 | 63 |
| Hipposideridae | *Macronycteris thomensis* | I | 0 | 1 |
| Hipposideridae | *Macronycteris vittatus* | I | 1 | 234 |
| Pteropodidae | *Megaloglossus azagnyi* | F | 5 | 60 |
| Pteropodidae | *Megaloglossus woermanni* | F | 2 | 156 |
| Vespertilionidae | *Mimetillus moloneyi* | I | 1 | 92 |
| Miniopteridae | *Miniopterus aelleni* | I | 0 | 29 |
| Miniopteridae | *Miniopterus africanus* | I | 0 | 29 |
| Miniopteridae | *Miniopterus ambohitrensis* | I | 0 | 11 |
| Miniopteridae | *Miniopterus arenarius* | I | 0 | 86 |
| Miniopteridae | *Miniopterus brachytragos* | I | 0 | 10 |
| Miniopteridae | *Miniopterus egeri* | I | 0 | 10 |
| Miniopteridae | *Miniopterus fraterculus* | I | 3 | 97 |
| Miniopteridae | *Miniopterus gleni* | I | 0 | 51 |
| Miniopteridae | *Miniopterus griffithsi* | I | 0 | 4 |
| Miniopteridae | *Miniopterus griveaudi* | I | 0 | 49 |
| Miniopteridae | *Miniopterus inflatus* | I | 0 | 123 |
| Miniopteridae | *Miniopterus maghrebensis* | I | 0 | 3 |
| Miniopteridae | *Miniopterus mahafaliensis* | I | 0 | 23 |
| Miniopteridae | *Miniopterus majori* | I | 2 | 27 |
| Miniopteridae | *Miniopterus manavi* | I | 4 | 25 |
| Miniopteridae | *Miniopterus minor* | I | 2 | 57 |
| Miniopteridae | *Miniopterus mossambicus* | I | 0 | 28 |
| Miniopteridae | *Miniopterus natalensis* | I | 3 | 422 |
| Miniopteridae | *Miniopterus newtoni* | I | 0 | 2 |
| Miniopteridae | *Miniopterus petersoni* | I | 0 | 19 |
| Miniopteridae | *Miniopterus schreibersii* | I | 1 | 31 |
| Miniopteridae | *Miniopterus sororculus* | I | 0 | 16 |
| Miniopteridae | *Miniopterus villiersi* | I | 0 | 13 |
| Molossidae | *Mops bakarii* | I | 0 | 1 |
| Molossidae | *Mops brachypterus* | I | 1 | 51 |
| Molossidae | *Mops condylurus* | I | 4 | 406 |
| Molossidae | *Mops congicus* | I | 0 | 19 |
| Molossidae | *Mops demonstrator* | I | 0 | 25 |
| Molossidae | *Mops leucostigma* | I | 4 | 22 |
| Molossidae | *Mops midas* | I | 1 | 82 |
| Molossidae | *Mops nanulus* | I | 0 | 59 |
| Molossidae | *Mops niangarae* | I | 0 | 1 |
| Molossidae | *Mops niveiventer* | I | 0 | 42 |
| Molossidae | *Mops petersoni* | I | 0 | 13 |
| Molossidae | *Mops spurrelli* | I | 0 | 46 |
| Molossidae | *Mops thersites* | I | 0 | 80 |
| Molossidae | *Mops trevori* | I | 0 | 16 |
| Molossidae | *Mormopterus acetabulosus* | I | 0 | 3 |
| Molossidae | *Mormopterus francoismoutoui* | I | 0 | 14 |
| Molossidae | *Mormopterus jugularis* | I | 1 | 36 |
| Pteropodidae | *Myonycteris brachycephala* | F | 4 | 5 |
| Pteropodidae | *Myonycteris leptodon* | F | 3 | 55 |
| Pteropodidae | *Myonycteris relicta* | F | 0 | 17 |
| Pteropodidae | *Myonycteris torquata* | F | 0 | 130 |
| Molossidae | *Myopterus daubentonii* | I | 1 | 8 |
| Molossidae | *Myopterus whitleyi* | I | 0 | 18 |
| Vespertilionidae | *Myotis anjouanensis* | I | 0 | 3 |
| Vespertilionidae | *Myotis bocagii* | I | 5 | 222 |
| Vespertilionidae | *Myotis capaccinii* | I | 4 | 4 |
| Vespertilionidae | *Myotis dieteri* | I | 0 | 1 |
| Vespertilionidae | *Myotis emarginatus* | I | 2 | 6 |
| Vespertilionidae | *Myotis goudotii* | I | 3 | 95 |
| Vespertilionidae | *Myotis morrisi* | I | 0 | 2 |
| Vespertilionidae | *Myotis mystacinus* | I | 3 | 3 |
| Vespertilionidae | *Myotis punicus* | I | 4 | 74 |
| Vespertilionidae | *Myotis scotti* | I | 0 | 3 |
| Vespertilionidae | *Myotis tricolor* | I | 5 | 133 |
| Vespertilionidae | *Myotis welwitschii* | I | 2 | 69 |
| Vespertilionidae | *Myotis zenatius* | I | 0 | 4 |
| Myzopodidae | *Myzopoda aurita* | I | 1 | 27 |
| Myzopodidae | *Myzopoda schliemanni* | I | 1 | 6 |
| Pteropodidae | *Nanonycteris veldkampii* | F | 0 | 100 |
| Vespertilionidae | *Neoromicia brunnea* | I | 0 | 31 |
| Vespertilionidae | *Neoromicia capensis* | I | 1 | 838 |
| Vespertilionidae | *Neoromicia grandidieri* | I | 0 | 29 |
| Vespertilionidae | *Neoromicia guineensis* | I | 0 | 49 |
| Vespertilionidae | *Neoromicia helios* | I | 0 | 37 |
| Vespertilionidae | *Neoromicia isabella* | I | 0 | 3 |
| Vespertilionidae | *Neoromicia malagasyensis* | I | 0 | 8 |
| Vespertilionidae | *Neoromicia matroka* | I | 6 | 20 |
| Vespertilionidae | *Neoromicia nanus* | I | 3 | 944 |
| Vespertilionidae | *Neoromicia rendalli* | I | 0 | 102 |
| Vespertilionidae | *Neoromicia robertsi* | I | 5 | 3 |
| Vespertilionidae | *Neoromicia roseveari* | I | 0 | 4 |
| Vespertilionidae | *Neoromicia somalica* | I | 0 | 138 |
| Vespertilionidae | *Neoromicia stanleyi* | I | 1 | 30 |
| Vespertilionidae | *Neoromicia tenuipinnis* | I | 0 | 167 |
| Vespertilionidae | *Neoromicia zuluensis* | I | 2 | 160 |
| Vespertilionidae | *Nyctalus lasiopterus* | I | 0 | 1 |
| Vespertilionidae | *Nyctalus leisleri* | I | 2 | 6 |
| Nycteridae | *Nycteris arge* | I | 0 | 180 |
| Nycteridae | *Nycteris aurita* | I | 0 | 36 |
| Nycteridae | *Nycteris gambiensis* | I | 0 | 66 |
| Nycteridae | *Nycteris grandis* | I | 0 | 148 |
| Nycteridae | *Nycteris hispida* | I | 2 | 495 |
| Nycteridae | *Nycteris intermedia* | I | 0 | 22 |
| Nycteridae | *Nycteris macrotis* | I | 4 | 343 |
| Nycteridae | *Nycteris madagascariensis* | I | 0 | 2 |
| Nycteridae | *Nycteris major* | I | 0 | 12 |
| Nycteridae | *Nycteris nana* | I | 0 | 62 |
| Nycteridae | *Nycteris parisii* | I | 0 | 9 |
| Nycteridae | *Nycteris thebaica* | I | 2 | 910 |
| Nycteridae | *Nycteris woodi* | I | 3 | 34 |
| Vespertilionidae | *Nycticeinops schlieffenii* | I | 6 | 289 |
| Molossidae | *Otomops harrisoni* | I | 1 | 16 |
| Molossidae | *Otomops madagascariensis* | I | 2 | 25 |
| Molossidae | *Otomops martiensseni* | I | 1 | 51 |
| Vespertilionidae | *Otonycteris hemprichii* | I | 6 | 26 |
| Rhinonycteridae | *Paratriaenops auritus* | I | 1 | 17 |
| Rhinonycteridae | *Paratriaenops furcula* | I | 1 | 14 |
| Rhinonycteridae | *Paratriaenops pauliani* | I | 0 | 3 |
| Emballonuridae | *Paremballonura atrata* | I | 0 | 20 |
| Emballonuridae | *Paremballonura tiavato* | I | 1 | 39 |
| Vespertilionidae | *Pipistrellus aero* | I | 0 | 5 |
| Vespertilionidae | *Pipistrellus hanaki* | I | 4 | 4 |
| Vespertilionidae | *Pipistrellus hesperidus* | I | 8 | 244 |
| Vespertilionidae | *Pipistrellus inexspectatus* | I | 0 | 19 |
| Vespertilionidae | *Pipistrellus kuhlii* | I | 7 | 120 |
| Vespertilionidae | *Pipistrellus nanulus* | I | 0 | 79 |
| Vespertilionidae | *Pipistrellus permixtus* | I | 0 | 1 |
| Vespertilionidae | *Pipistrellus pipistrellus* | I | 1 | 18 |
| Vespertilionidae | *Pipistrellus raceyi* | I | 4 | 16 |
| Vespertilionidae | *Pipistrellus rusticus* | I | 3 | 134 |
| Molossidae | *Platymops setiger* | I | 1 | 46 |
| Vespertilionidae | *Plecotus balensis* | I | 0 | 4 |
| Vespertilionidae | *Plecotus christii* | I | 1 | 26 |
| Vespertilionidae | *Plecotus gaisleri* | I | 1 | 4 |
| Vespertilionidae | *Plecotus teneriffae* | I | 1 | 29 |
| Pteropodidae | *Plerotes anchietae* | F | 0 | 13 |
| Pteropodidae | *Pteropus aldabrensis* | F | 7 | 9 |
| Pteropodidae | *Pteropus livingstonii* | F | 1 | 6 |
| Pteropodidae | *Pteropus niger* | F | 0 | 10 |
| Pteropodidae | *Pteropus rodricensis* | F | 8 | 10 |
| Pteropodidae | *Pteropus rufus* | F | 0 | 48 |
| Pteropodidae | *Pteropus seychellensis* | F | 9 | 22 |
| Pteropodidae | *Pteropus voeltzkowi* | F | 1 | 13 |
| Rhinolophidae | *Rhinolophus adami* | I | 0 | 3 |
| Rhinolophidae | *Rhinolophus alcyone* | I | 0 | 72 |
| Rhinolophidae | *Rhinolophus alticolus* | I | 0 | 1 |
| Rhinolophidae | *Rhinolophus blasii* | I | 1 | 94 |
| Rhinolophidae | *Rhinolophus capensis* | I | 2 | 40 |
| Rhinolophidae | *Rhinolophus clivosus* | I | 2 | 472 |
| Rhinolophidae | *Rhinolophus cohenae* | I | 2 | 5 |
| Rhinolophidae | *Rhinolophus damarensis* | I | 2 | 54 |
| Rhinolophidae | *Rhinolophus darlingi* | I | 2 | 164 |
| Rhinolophidae | *Rhinolophus deckenii* | I | 0 | 43 |
| Rhinolophidae | *Rhinolophus denti* | I | 1 | 35 |
| Rhinolophidae | *Rhinolophus eloquens* | I | 0 | 86 |
| Rhinolophidae | *Rhinolophus euryale* | I | 1 | 18 |
| Rhinolophidae | *Rhinolophus ferrumequinum* | I | 4 | 32 |
| Rhinolophidae | *Rhinolophus fumigatus* | I | 2 | 246 |
| Rhinolophidae | *Rhinolophus gorongosae* | I | 0 | 5 |
| Rhinolophidae | *Rhinolophus guineensis* | I | 0 | 14 |
| Rhinolophidae | *Rhinolophus hildebrandtii* | I | 2 | 97 |
| Rhinolophidae | *Rhinolophus hilli* | I | 0 | 2 |
| Rhinolophidae | *Rhinolophus hillorum* | I | 0 | 7 |
| Rhinolophidae | *Rhinolophus hipposideros* | I | 1 | 28 |
| Rhinolophidae | *Rhinolophus horaceki* | I | 1 | 0 |
| Rhinolophidae | *Rhinolophus kahuzi* | I | 0 | 1 |
| Rhinolophidae | *Rhinolophus landeri* | I | 2 | 116 |
| Rhinolophidae | *Rhinolophus lobatus* | I | 1 | 142 |
| Rhinolophidae | *Rhinolophus mabuensis* | I | 0 | 2 |
| Rhinolophidae | *Rhinolophus maclaudi* | I | 0 | 2 |
| Rhinolophidae | *Rhinolophus maendeleo* | I | 0 | 9 |
| Rhinolophidae | *Rhinolophus mehelyi* | I | 1 | 32 |
| Rhinolophidae | *Rhinolophus mossambicus* | I | 2 | 10 |
| Rhinolophidae | *Rhinolophus rhodesiae* | I | 1 | 63 |
| Rhinolophidae | *Rhinolophus ruwenzorii* | I | 1 | 17 |
| Rhinolophidae | *Rhinolophus sakejiensis* | I | 0 | 2 |
| Rhinolophidae | *Rhinolophus silvestris* | I | 0 | 3 |
| Rhinolophidae | *Rhinolophus simulator* | I | 1 | 203 |
| Rhinolophidae | *Rhinolophus smithersi* | I | 2 | 182 |
| Rhinolophidae | *Rhinolophus swinnyi* | I | 1 | 21 |
| Rhinolophidae | *Rhinolophus willardi* | I | 0 | 4 |
| Rhinolophidae | *Rhinolophus ziama* | I | 0 | 2 |
| Rhinopomatidae | *Rhinopoma cystops* | I | 0 | 77 |
| Rhinopomatidae | *Rhinopoma macinnesi* | I | 0 | 15 |
| Rhinopomatidae | *Rhinopoma microphyllum* | I | 1 | 33 |
| Pteropodidae | *Rousettus aegyptiacus* | F | 3 | 466 |
| Pteropodidae | *Rousettus madagascariensis* | F | 3 | 37 |
| Pteropodidae | *Rousettus obliviosus* | F | 6 | 25 |
| Emballonuridae | *Saccolaimus peli* | I | 2 | 52 |
| Molossidae | *Sauromys petrophilus* | I | 4 | 96 |
| Vespertilionidae | *Scotoecus albofuscus* | I | 1 | 24 |
| Vespertilionidae | *Scotoecus hirundo* | I | 0 | 145 |
| Pteropodidae | *Scotonycteris bergmansi* | F | 1 | 31 |
| Pteropodidae | *Scotonycteris occidentalis* | F | 1 | 33 |
| Pteropodidae | *Scotonycteris zenkeri* | F | 0 | 62 |
| Vespertilionidae | *Scotophilus altilis* | I | 0 | 11 |
| Vespertilionidae | *Scotophilus andrewreborii* | I | 0 | 13 |
| Vespertilionidae | *Scotophilus borbonicus* | I | 0 | 1 |
| Vespertilionidae | *Scotophilus colias* | I | 0 | 10 |
| Vespertilionidae | *Scotophilus dinganii* | I | 4 | 660 |
| Vespertilionidae | *Scotophilus ejetai* | I | 0 | 2 |
| Vespertilionidae | *Scotophilus leucogaster* | I | 3 | 190 |
| Vespertilionidae | *Scotophilus livingstonii* | I | 0 | 5 |
| Vespertilionidae | *Scotophilus marovaza* | I | 0 | 6 |
| Vespertilionidae | *Scotophilus nigrita* | I | 0 | 48 |
| Vespertilionidae | *Scotophilus nigritellus* | I | 0 | 44 |
| Vespertilionidae | *Scotophilus nucella* | I | 0 | 12 |
| Vespertilionidae | *Scotophilus nux* | I | 0 | 35 |
| Vespertilionidae | *Scotophilus robustus* | I | 1 | 21 |
| Vespertilionidae | *Scotophilus tandrefana* | I | 0 | 1 |
| Vespertilionidae | *Scotophilus trujilloi* | I | 0 | 7 |
| Vespertilionidae | *Scotophilus viridis* | I | 1 | 123 |
| Pteropodidae | *Stenonycteris lanosus* | F | 0 | 62 |
| Molossidae | *Tadarida aegyptiaca* | I | 7 | 336 |
| Molossidae | *Tadarida fulminans* | I | 0 | 35 |
| Molossidae | *Tadarida lobata* | I | 0 | 20 |
| Molossidae | *Tadarida teniotis* | I | 1 | 16 |
| Molossidae | *Tadarida ventralis* | I | 0 | 27 |
| Emballonuridae | *Taphozous hamiltoni* | I | 0 | 12 |
| Emballonuridae | *Taphozous hildegardeae* | I | 2 | 14 |
| Emballonuridae | *Taphozous mauritianus* | I | 1 | 318 |
| Emballonuridae | *Taphozous nudiventris* | I | 4 | 50 |
| Emballonuridae | *Taphozous perforatus* | I | 1 | 96 |
| Rhinonycteridae | *Triaenops afer* | I | 1 | 76 |
| Rhinonycteridae | *Triaenops menamena* | I | 1 | 58 |
| Vespertilionidae | *Vansonia rueppellii* | I | 5 | 133 |

Table S1.5. Set of variables considered for the analysis.

| Variable | Source | Original resolution | Processing |
| --- | --- | --- | --- |
| 19 Bioclimatic | Chelsa V2.1 | 30 arc-seconds | Same as original resolution. |
| TRI index | SRTM v4.1 | 90 m | -Upscaling TRI index at 30 arc-seconds for bats SDMs. |
| Distance to permanent and temporary water | -Vectors: World Waterbodies, and World Waterlines databases (ESRI ©); AQUAMAPS Rivers of Africa database  -Raster: Global Surface Water database | 30 m | -Merge all dataset;  -Upscaling at 30 arc-seconds for bats SDMs;  -Calculating planar distance from water layers for bats SDMs. |
| Human population density | SEDAC database | 30 arc-seconds | Same as original resolution. |
| Richness of trophic resource | This study | 0.5 decimal degree | -Models projected at 30 arc-seconds for bats SDMs. |

**APPENDIX S2**

Table S2.6. Results of Variance Inflation Factor (VIF) for frugivore (A) and insectivore (B) bats.

1. Frugivores

| Variable | VIF |
| --- | --- |
| Mean Temperature of Wettest Quarter (bio8) | 1.70 |
| Mean Temperature of Driest Quarter (bio9) | 1.20 |
| Precipitation of Driest Month (bio14) | 1.56 |
| Precipitation Seasonality (bio15) | 1.81 |
| Precipitation of Coldest Quarter (bio19) | 1.62 |
| Richness of Plants | 2.12 |
| Distance to Permanent Water (Dperm) | 2.14 |
| Distance to Temporary Water (Dtemp) | 1.18 |
| Terrain Ruggedness Index (TRI) | 1.20 |
| Human Population Density (HumPop) | 1.02 |

1. Insectivores

| Variable | VIF |
| --- | --- |
| Mean Temperature of Wettest Quarter (bio8) | 1.55 |
| Mean Temperature of Driest Quarter (bio9) | 1.26 |
| Precipitation of Driest Month (bio14) | 1.61 |
| Precipitation Seasonality (bio15) | 1.85 |
| Precipitation of Coldest Quarter (bio19) | 1.65 |
| Richness of Arthropods | 1.86 |
| Distance to Permanent Water (Dperm) | 1.90 |
| Distance to Temporary Water (Dtemp) | 1.20 |
| Terrain Ruggedness Index (TRI) | 1.22 |
| Human Population Density (HumPop) | 1.02 |

Figure S2.7. Boxplots of explanatory variables in random versus target-group selected background. a) Plants richness; b) Arthropods richness; c) Mean Temperature of Wettest Quarter; d) Mean Temperature of Driest Quarter; e) Precipitation of Driest Month; f) Precipitation Seasonality; g) Precipitation of Coldest Quarter; h) Terrain Ruggedness Index; i) Distance to Permanent Water; j) Distance to Temporary Water; k) Human Population Density.


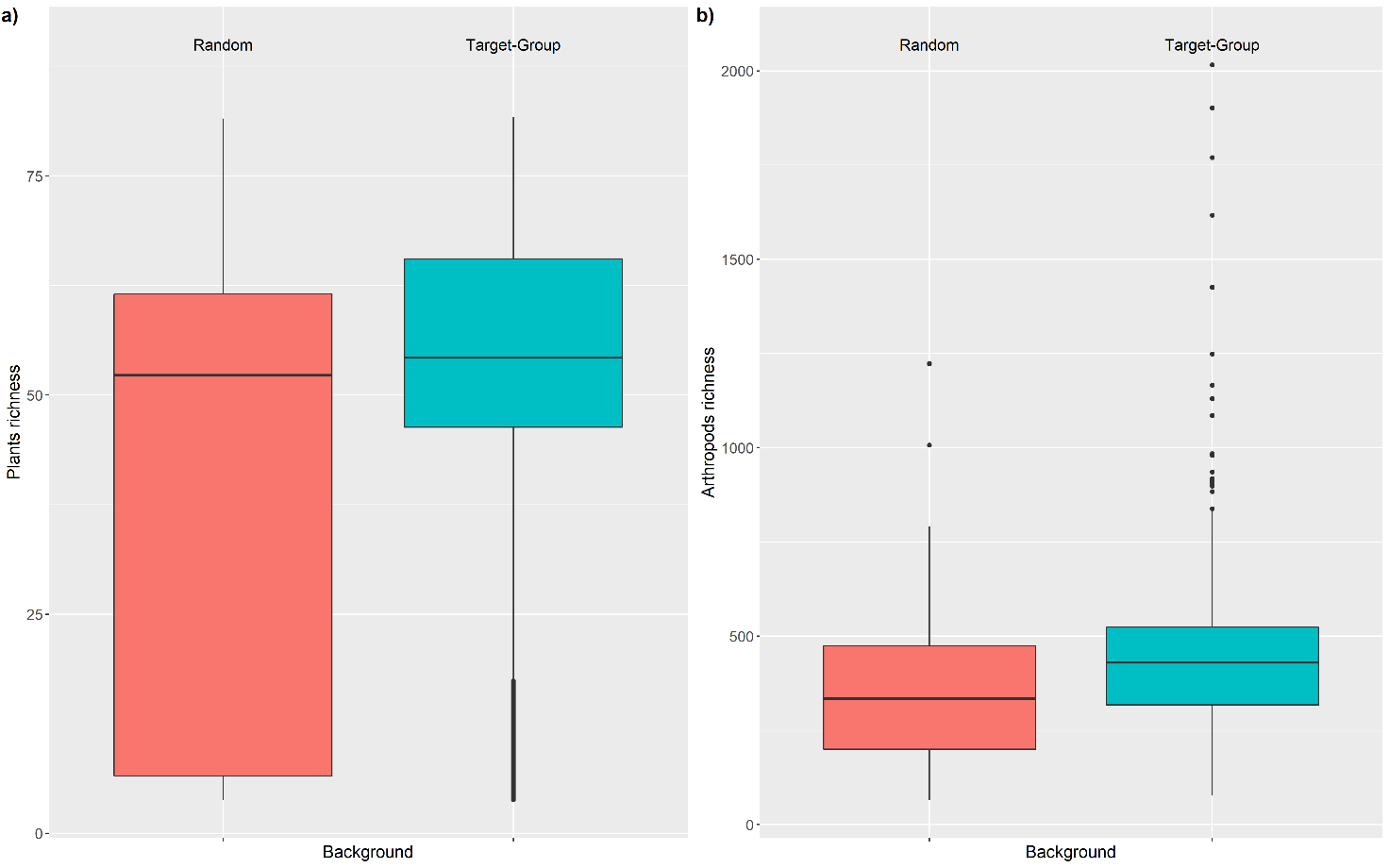


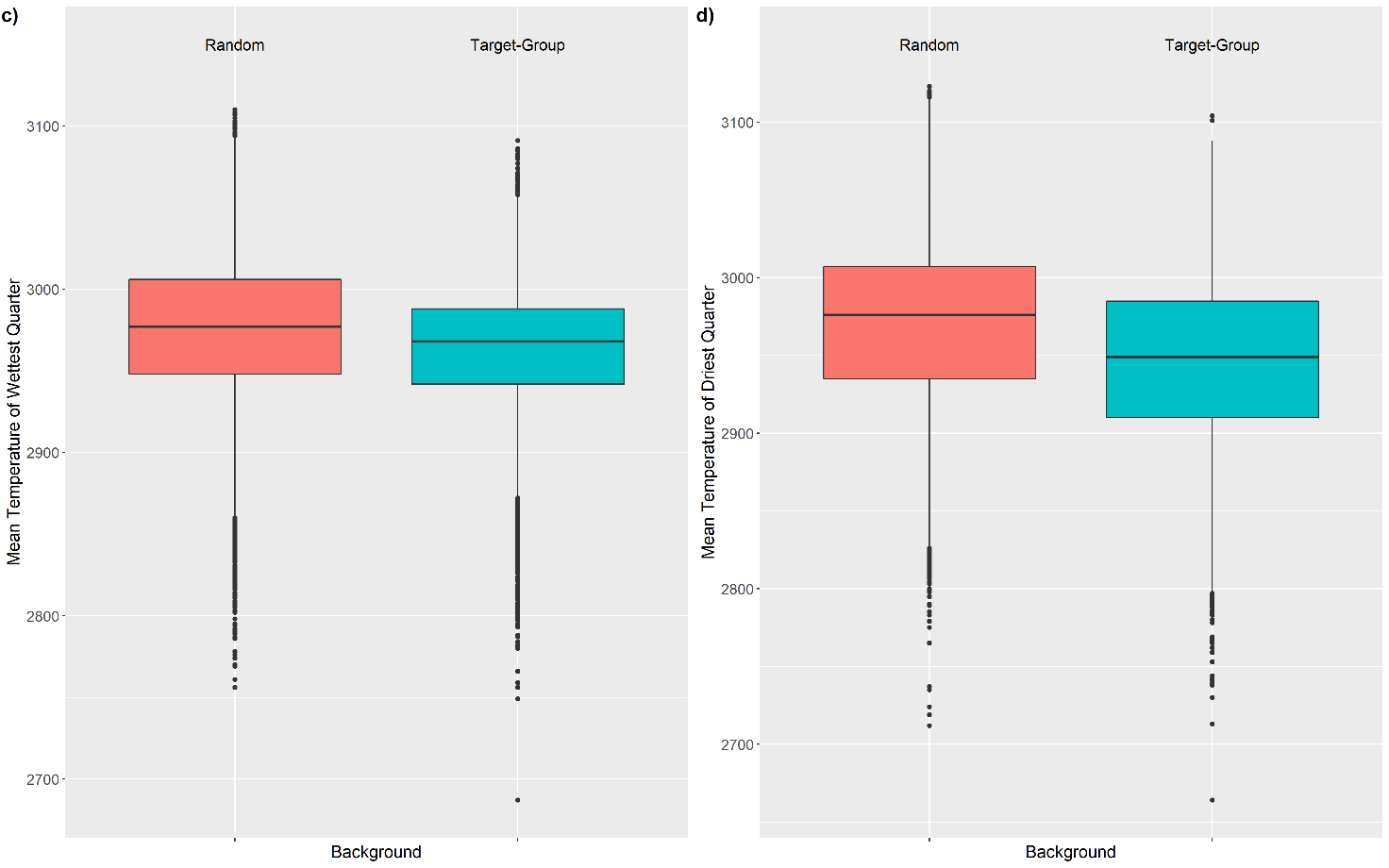


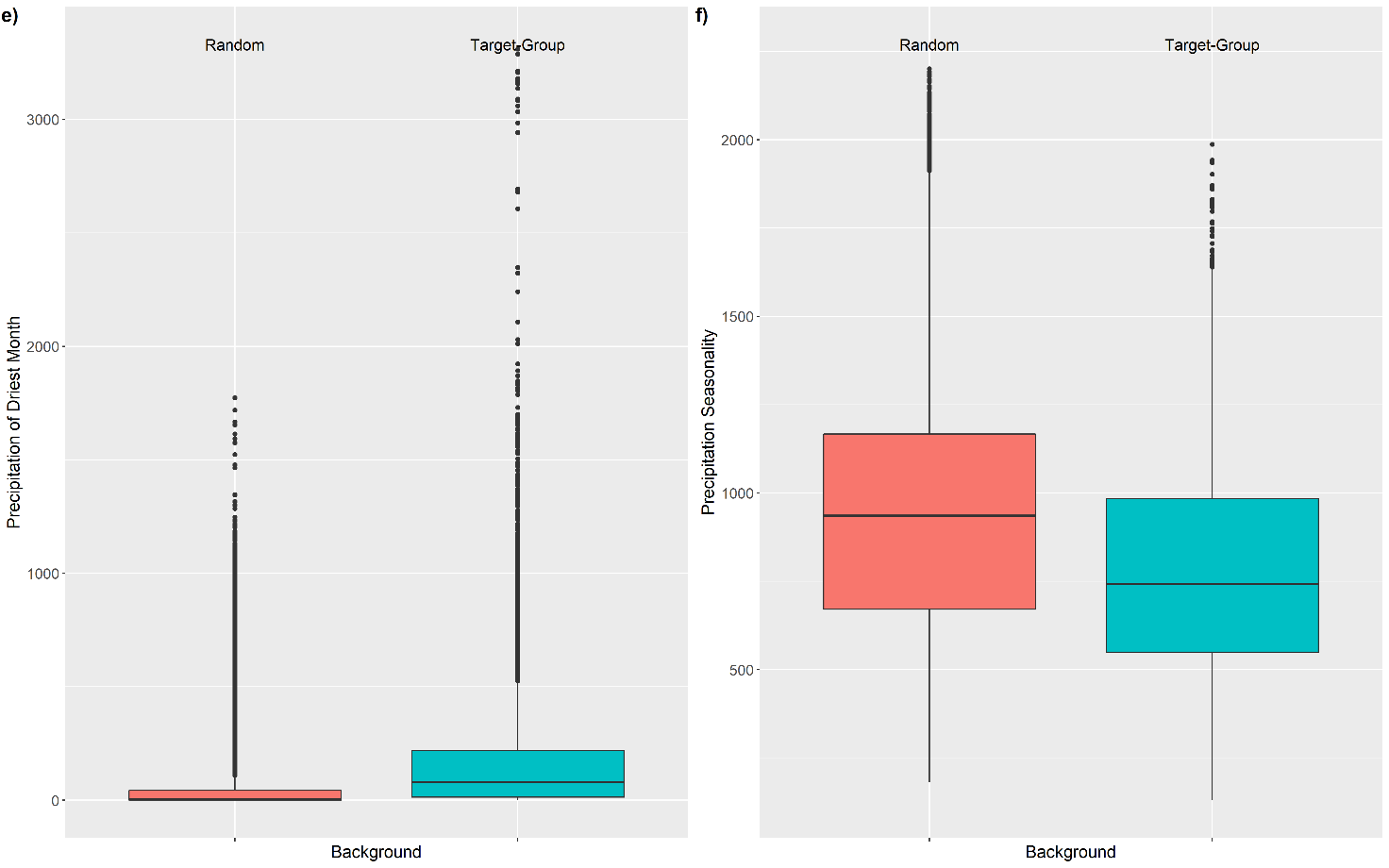


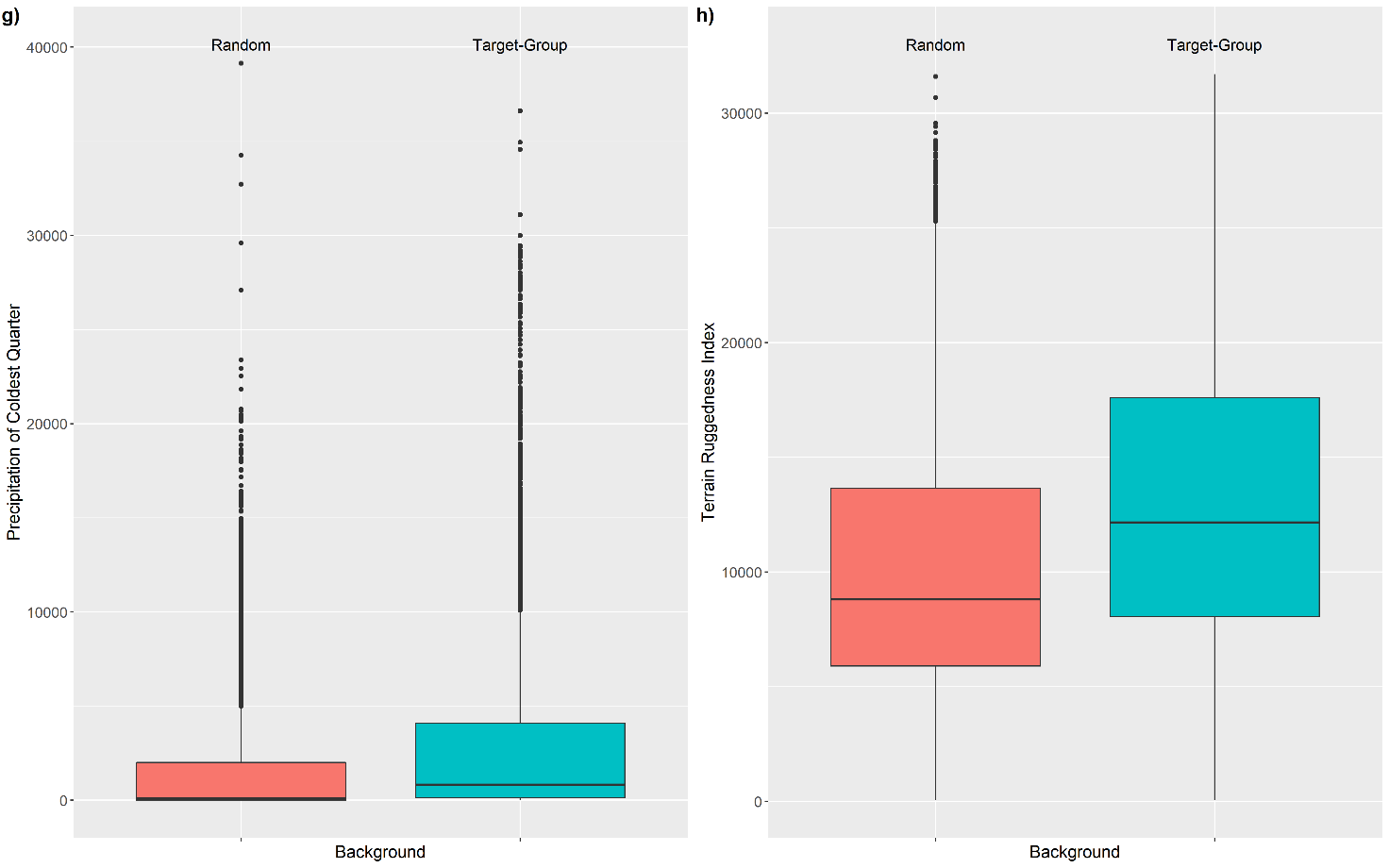


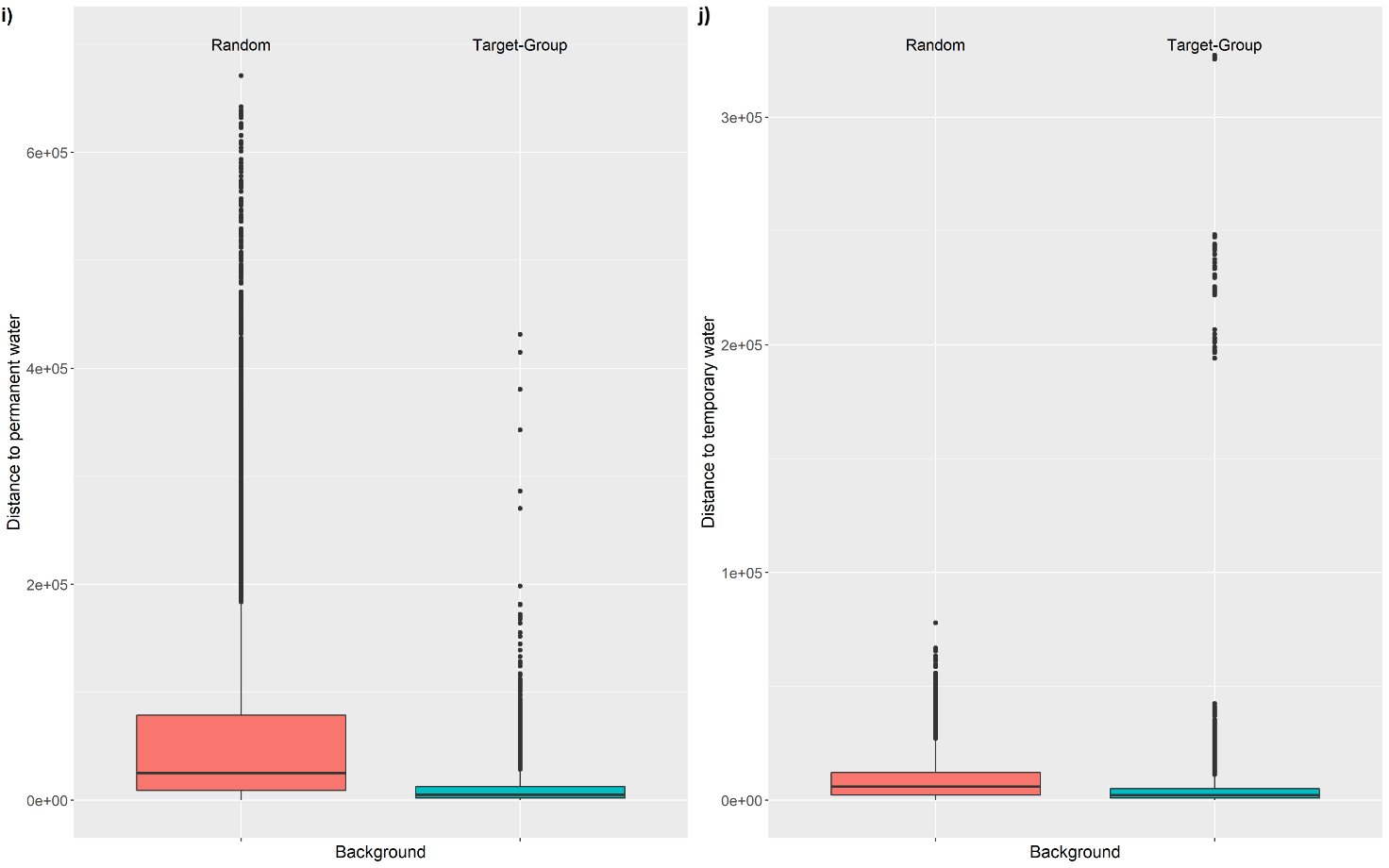


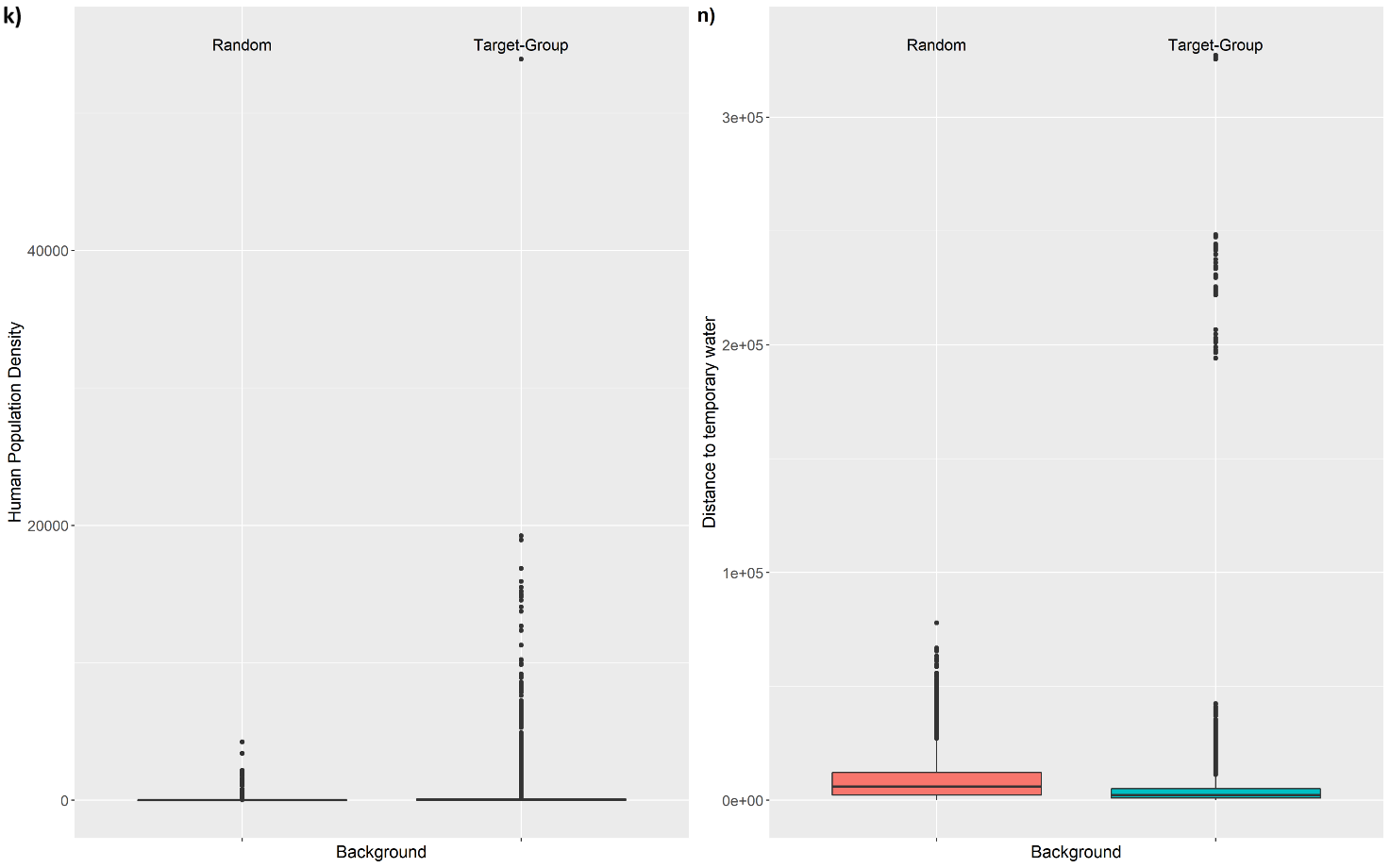


Figure S2.8. FAO Global Ecological Zones (FAO, 2012) classified in 15 biogeographically homogeneous areas for the present study.


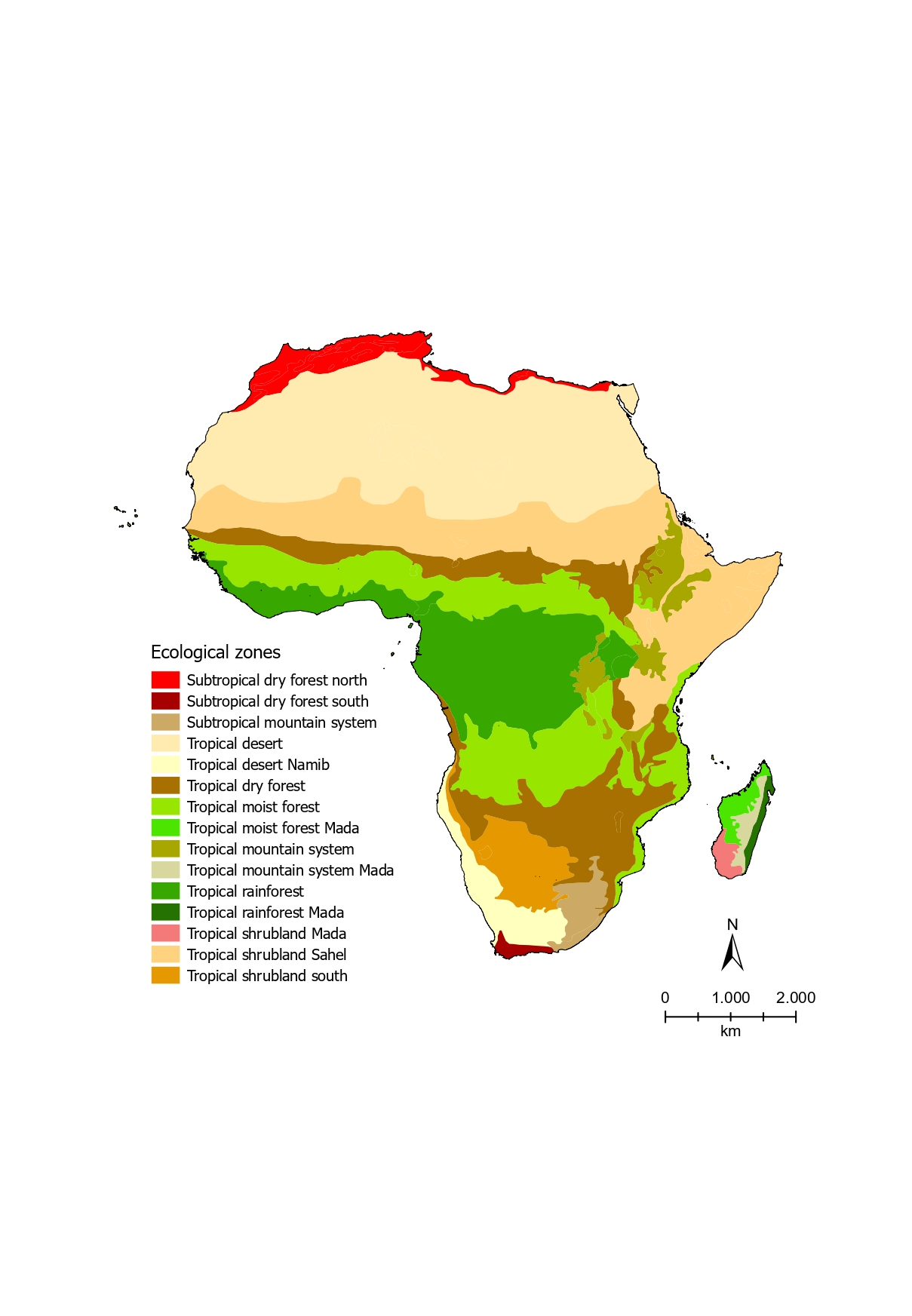


**APPENDIX S3**

Table S3.9. Species-specific average and standard deviation AUC, TSS, and Boyce index calculated over 10 replicates for models calibrated with (“_BIO”) and without the trophic resource variable (“_noBIO”).

| Species | AUC_BIO | AUC_noBIO | TSS_BIO | TSS_noBIO | Boyce_BIO | Boyce_noBIO |
| --- | --- | --- | --- | --- | --- | --- |
| *Asellia tridens* | 0.87±0.05 | 0.85±0.07 | 0.78±0.07 | 0.71±0.06 | 0.93±0.05 | 0.90±0.11 |
| *Cardioderma cor* | 0.93±0.03 | 0.89±0.04 | 0.79±0.08 | 0.71±0.06 | 0.91±0.06 | 0.87±0.10 |
| *Casinycteris argynnis* | 0.96±0.02 | 0.96±0.02 | 0.84±0.10 | 0.77±0.11 | 0.86±0.17 | 0.84±0.14 |
| *Chaerephon aloysiisabaudiae* | 0.94±0.02 | 0.96±0.02 | 0.87±0.10 | 0.82±0.12 | 0.88±0.16 | 0.84±0.13 |
| *Chaerephon ansorgei* | 0.89±0.03 | 0.84±0.04 | 0.84±0.11 | 0.80±0.11 | 0.89±0.14 | 0.87±0.13 |
| *Chaerephon atsinanana* | 0.99±0 | 0.99±0 | 0.86±0.12 | 0.83±0.13 | 0.88±0.14 | 0.86±0.12 |
| *Chaerephon bivittatus* | 0.9±0.03 | 0.91±0.03 | 0.85±0.12 | 0.82±0.12 | 0.89±0.13 | 0.87±0.12 |
| *Chaerephon chapini* | 0.77±0.09 | 0.83±0.07 | 0.83±0.13 | 0.82±0.12 | 0.89±0.13 | 0.87±0.11 |
| *Chaerephon leucogaster* | 0.94±0.03 | 0.94±0.03 | 0.84±0.12 | 0.83±0.11 | 0.90±0.12 | 0.87±0.11 |
| *Chaerephon major* | 0.88±0.04 | 0.85±0.05 | 0.83±0.12 | 0.82±0.11 | 0.89±0.12 | 0.87±0.11 |
| *Chaerephon nigeriae* | 0.86±0.04 | 0.82±0.05 | 0.82±0.12 | 0.81±0.12 | 0.88±0.12 | 0.87±0.11 |
| *Chaerephon pumilus* | 0.90±0.01 | 0.9±0.01 | 0.81±0.12 | 0.80±0.12 | 0.89±0.12 | 0.88±0.11 |
| *Chaerephon pusillus* | 0.97±0.02 | 0.94±0.04 | 0.82±0.12 | 0.81±0.12 | 0.90±0.11 | 0.88±0.11 |
| *Cistugo lesueuri* | 0.99±0 | 0.99±0 | 0.83±0.13 | 0.82±0.13 | 0.89±0.11 | 0.88±0.11 |
| *Cloeotis percivali* | 0.96±0.02 | 0.94±0.03 | 0.84±0.12 | 0.83±0.13 | 0.90±0.11 | 0.89±0.11 |
| *Coleura afra* | 0.90±0.03 | 0.89±0.03 | 0.83±0.13 | 0.82±0.13 | 0.89±0.11 | 0.88±0.11 |
| *Doryrhina cyclops* | 0.95±0.01 | 0.94±0.02 | 0.83±0.12 | 0.82±0.12 | 0.90±0.11 | 0.89±0.11 |
| *Eidolon dupreanum* | 0.96±0.02 | 0.98±0.01 | 0.84±0.12 | 0.83±0.12 | 0.90±0.11 | 0.89±0.11 |
| *Eidolon helvum* | 0.89±0.02 | 0.89±0.02 | 0.83±0.13 | 0.82±0.13 | 0.90±0.11 | 0.89±0.11 |
| *Epomophorus angolensis* | 0.96±0.02 | 0.95±0.02 | 0.83±0.13 | 0.83±0.13 | 0.90±0.11 | 0.89±0.12 |
| *Epomophorus crypturus* | 0.94±0.01 | 0.93±0.02 | 0.83±0.12 | 0.83±0.12 | 0.91±0.10 | 0.89±0.12 |
| *Epomophorus dobsonii* | 0.97±0.01 | 0.95±0.02 | 0.83±0.12 | 0.83±0.12 | 0.91±0.10 | 0.89±0.12 |
| *Epomophorus gambianus* | 0.93±0.01 | 0.93±0.01 | 0.83±0.12 | 0.83±0.12 | 0.91±0.10 | 0.89±0.12 |
| *Epomophorus labiatus* | 0.92±0.01 | 0.89±0.02 | 0.83±0.12 | 0.82±0.12 | 0.91±0.10 | 0.90±0.12 |
| *Epomophorus minor* | 0.91±0.02 | 0.88±0.04 | 0.83±0.12 | 0.82±0.12 | 0.91±0.10 | 0.90±0.12 |
| *Epomophorus pusillus* | 0.93±0.01 | 0.92±0.01 | 0.82±0.12 | 0.82±0.12 | 0.92±0.10 | 0.90±0.11 |
| *Epomophorus wahlbergi* | 0.92±0.01 | 0.91±0.01 | 0.82±0.11 | 0.81±0.12 | 0.92±0.10 | 0.91±0.11 |
| *Epomops buettikoferi* | 0.98±0.01 | 0.97±0.01 | 0.83±0.11 | 0.82±0.12 | 0.92±0.10 | 0.91±0.11 |
| *Epomops franqueti* | 0.95±0.01 | 0.93±0.01 | 0.83±0.11 | 0.82±0.12 | 0.92±0.10 | 0.91±0.11 |
| *Eptesicus hottentotus* | 0.93±0.02 | 0.93±0.03 | 0.83±0.11 | 0.82±0.12 | 0.92±0.10 | 0.91±0.11 |
| *Eptesicus isabellinus* | 0.98±0.01 | 0.98±0.01 | 0.83±0.11 | 0.82±0.12 | 0.92±0.10 | 0.91±0.11 |
| *Glauconycteris argentata* | 0.94±0.03 | 0.94±0.03 | 0.83±0.11 | 0.82±0.12 | 0.92±0.10 | 0.91±0.11 |
| *Glauconycteris beatrix* | 0.94±0.03 | 0.93±0.03 | 0.84±0.11 | 0.82±0.11 | 0.91±0.11 | 0.90±0.13 |
| *Glauconycteris poensis* | 0.94±0.03 | 0.93±0.04 | 0.84±0.11 | 0.83±0.11 | 0.91±0.11 | 0.90±0.13 |
| *Glauconycteris variegata* | 0.89±0.03 | 0.87±0.04 | 0.83±0.11 | 0.82±0.12 | 0.91±0.11 | 0.90±0.13 |
| *Hipposideros abae* | 0.93±0.02 | 0.92±0.03 | 0.84±0.11 | 0.82±0.11 | 0.91±0.11 | 0.90±0.13 |
| *Hipposideros beatus* | 0.95±0.02 | 0.95±0.01 | 0.84±0.11 | 0.83±0.11 | 0.91±0.11 | 0.90±0.13 |
| *Hipposideros caffer* | 0.88±0.01 | 0.87±0.01 | 0.83±0.11 | 0.82±0.12 | 0.92±0.11 | 0.91±0.12 |
| *Hipposideros fuliginosus* | 0.95±0.02 | 0.94±0.03 | 0.83±0.11 | 0.82±0.12 | 0.92±0.11 | 0.91±0.12 |
| *Hipposideros jonesi* | 0.95±0.02 | 0.90±0.04 | 0.84±0.11 | 0.82±0.11 | 0.91±0.11 | 0.90±0.14 |
| *Hipposideros ruber* | 0.90±0.01 | 0.87±0.01 | 0.83±0.11 | 0.82±0.12 | 0.92±0.11 | 0.90±0.14 |
| *Hipposideros tephrus* | 0.88±0.03 | 0.88±0.04 | 0.83±0.11 | 0.82±0.12 | 0.92±0.11 | 0.90±0.14 |
| *Hypsignathus monstrosus* | 0.95±0.01 | 0.95±0.01 | 0.83±0.11 | 0.82±0.11 | 0.92±0.11 | 0.90±0.14 |
| *Hypsugo anchieta* | 0.95±0.02 | 0.93±0.03 | 0.83±0.11 | 0.82±0.11 | 0.92±0.10 | 0.90±0.13 |
| *Hypsugo crassulus* | 0.93±0.02 | 0.93±0.02 | 0.83±0.11 | 0.82±0.11 | 0.91±0.11 | 0.90±0.13 |
| *Kerivoula argentata* | 0.95±0.02 | 0.93±0.03 | 0.83±0.11 | 0.82±0.11 | 0.91±0.11 | 0.90±0.13 |
| *Kerivoula lanosa* | 0.90±0.04 | 0.90±0.03 | 0.83±0.11 | 0.82±0.11 | 0.91±0.11 | 0.90±0.13 |
| *Laephotis botswanae* | 0.94±0.02 | 0.94±0.02 | 0.83±0.11 | 0.82±0.11 | 0.91±0.11 | 0.89±0.15 |
| *Laephotis wintoni* | 0.97±0.02 | 0.92±0.04 | 0.84±0.11 | 0.83±0.11 | 0.91±0.11 | 0.90±0.15 |
| *Lavia frons* | 0.89±0.02 | 0.89±0.02 | 0.83±0.11 | 0.82±0.11 | 0.92±0.11 | 0.90±0.15 |
| *Lissonycteris angolensis* | 0.94±0.01 | 0.92±0.01 | 0.83±0.11 | 0.82±0.11 | 0.92±0.11 | 0.90±0.15 |
| *Macronycteris commersonii* | 0.99±0 | 0.99±0 | 0.84±0.11 | 0.82±0.11 | 0.92±0.11 | 0.90±0.15 |
| *Macronycteris gigas* | 0.92±0.03 | 0.9±0.03 | 0.84±0.11 | 0.82±0.11 | 0.92±0.11 | 0.90±0.15 |
| *Macronycteris vittatus* | 0.89±0.02 | 0.87±0.02 | 0.83±0.11 | 0.82±0.11 | 0.92±0.11 | 0.90±0.15 |
| *Megaloglossus azagnyi* | 0.99±0 | 0.98±0.01 | 0.84±0.11 | 0.82±0.11 | 0.91±0.11 | 0.90±0.15 |
| *Megaloglossus woermanni* | 0.96±0.01 | 0.96±0.01 | 0.84±0.11 | 0.82±0.11 | 0.91±0.11 | 0.90±0.15 |
| *Mimetillus moloneyi* | 0.92±0.02 | 0.92±0.02 | 0.84±0.11 | 0.82±0.11 | 0.91±0.11 | 0.90±0.15 |
| *Miniopterus aelleni* | 0.98±0.01 | 0.98±0.01 | 0.84±0.11 | 0.83±0.11 | 0.91±0.12 | 0.90±0.15 |
| *Miniopterus africanus* | 0.94±0.03 | 0.94±0.03 | 0.84±0.11 | 0.83±0.11 | 0.91±0.12 | 0.90±0.14 |
| *Miniopterus arenarius* | 0.96±0.01 | 0.94±0.02 | 0.84±0.11 | 0.83±0.11 | 0.91±0.12 | 0.90±0.14 |
| *Miniopterus fraterculus* | 0.95±0.02 | 0.94±0.02 | 0.84±0.11 | 0.83±0.11 | 0.91±0.12 | 0.90±0.14 |
| *Miniopterus gleni* | 0.99±0 | 0.99±0 | 0.84±0.11 | 0.83±0.11 | 0.91±0.12 | 0.90±0.14 |
| *Miniopterus griveaudi* | 0.99±0 | 0.99±0 | 0.84±0.11 | 0.83±0.11 | 0.91±0.12 | 0.90±0.14 |
| *Miniopterus inflatus* | 0.89±0.03 | 0.91±0.03 | 0.84±0.11 | 0.83±0.11 | 0.91±0.12 | 0.90±0.14 |
| *Miniopterus mahafaliensis* | 0.99±0 | 0.99±0.01 | 0.85±0.11 | 0.83±0.11 | 0.91±0.12 | 0.90±0.14 |
| *Miniopterus majori* | 0.98±0.01 | 0.96±0.03 | 0.85±0.11 | 0.84±0.11 | 0.90±0.12 | 0.89±0.15 |
| *Miniopterus manavi* | 0.98±0.01 | 0.98±0.01 | 0.85±0.11 | 0.84±0.12 | 0.90±0.13 | 0.89±0.15 |
| *Miniopterus minor* | 0.92±0.04 | 0.92±0.04 | 0.85±0.11 | 0.84±0.11 | 0.90±0.13 | 0.89±0.15 |
| *Miniopterus mossambicus* | 0.97±0.01 | 0.97±0.01 | 0.85±0.11 | 0.84±0.11 | 0.90±0.13 | 0.89±0.15 |
| *Miniopterus natalensis* | 0.92±0.01 | 0.91±0.01 | 0.85±0.11 | 0.84±0.11 | 0.90±0.13 | 0.89±0.15 |
| *Miniopterus schreibersii* | 0.99±0 | 0.99±0 | 0.85±0.11 | 0.84±0.11 | 0.90±0.14 | 0.89±0.15 |
| *Mops brachypterus* | 0.93±0.04 | 0.92±0.04 | 0.85±0.11 | 0.84±0.11 | 0.90±0.14 | 0.89±0.15 |
| *Mops condylurus* | 0.90±0.01 | 0.91±0.01 | 0.85±0.11 | 0.84±0.11 | 0.90±0.14 | 0.89±0.15 |
| *Mops demonstrator* | 0.89±0.05 | 0.90±0.04 | 0.85±0.11 | 0.84±0.11 | 0.90±0.14 | 0.89±0.15 |
| *Mops leucostigma* | 0.99±0.01 | 0.99±0.01 | 0.85±0.11 | 0.84±0.11 | 0.90±0.14 | 0.89±0.15 |
| *Mops midas* | 0.90±0.04 | 0.88±0.04 | 0.85±0.11 | 0.84±0.12 | 0.90±0.14 | 0.89±0.15 |
| *Mops nanulus* | 0.96±0.02 | 0.95±0.02 | 0.85±0.11 | 0.84±0.12 | 0.90±0.14 | 0.89±0.15 |
| *Mops niveiventer* | 0.97±0.02 | 0.96±0.02 | 0.85±0.11 | 0.84±0.11 | 0.90±0.14 | 0.89±0.14 |
| *Mops spurrelli* | 0.98±0.01 | 0.97±0.01 | 0.85±0.11 | 0.84±0.11 | 0.90±0.14 | 0.89±0.14 |
| *Mops thersites* | 0.95±0.02 | 0.95±0.02 | 0.85±0.11 | 0.84±0.11 | 0.89±0.14 | 0.89±0.15 |
| *Mormopterus jugularis* | 0.99±0 | 0.98±0.01 | 0.86±0.11 | 0.84±0.11 | 0.89±0.14 | 0.89±0.15 |
| *Myonycteris leptodon* | 0.98±0 | 0.97±0.01 | 0.86±0.11 | 0.84±0.11 | 0.89±0.14 | 0.89±0.15 |
| *Myonycteris torquata* | 0.94±0.02 | 0.93±0.02 | 0.86±0.11 | 0.84±0.11 | 0.89±0.14 | 0.89±0.15 |
| *Myotis bocagii* | 0.92±0.01 | 0.91±0.02 | 0.85±0.11 | 0.84±0.11 | 0.90±0.14 | 0.89±0.14 |
| *Myotis goudotii* | 0.99±0 | 0.98±0 | 0.86±0.11 | 0.84±0.11 | 0.90±0.14 | 0.89±0.14 |
| *Myotis punicus* | 0.99±0 | 0.99±0 | 0.86±0.11 | 0.85±0.11 | 0.89±0.14 | 0.89±0.14 |
| *Myotis tricolor* | 0.96±0.01 | 0.96±0.01 | 0.86±0.11 | 0.85±0.11 | 0.90±0.13 | 0.89±0.14 |
| *Myotis welwitschii* | 0.94±0.02 | 0.93±0.02 | 0.86±0.11 | 0.85±0.11 | 0.90±0.13 | 0.89±0.14 |
| *Myzopoda aurita* | 0.99±0 | 0.99±0.01 | 0.86±0.11 | 0.85±0.11 | 0.89±0.14 | 0.89±0.15 |
| *Nanonycteris veldkampii* | 0.98±0.01 | 0.96±0.01 | 0.86±0.11 | 0.85±0.11 | 0.89±0.14 | 0.89±0.15 |
| *Neoromicia brunnea* | 0.94±0.04 | 0.95±0.03 | 0.86±0.11 | 0.85±0.11 | 0.89±0.14 | 0.89±0.15 |
| *Neoromicia capensis* | 0.91±0.01 | 0.91±0.01 | 0.86±0.11 | 0.85±0.11 | 0.89±0.14 | 0.89±0.15 |
| *Neoromicia grandidieri* | 0.95±0.03 | 0.93±0.05 | 0.86±0.11 | 0.85±0.11 | 0.89±0.14 | 0.89±0.15 |
| *Neoromicia guineensis* | 0.91±0.04 | 0.89±0.05 | 0.86±0.11 | 0.85±0.11 | 0.89±0.14 | 0.89±0.15 |
| *Neoromicia helios* | 0.92±0.04 | 0.90±0.04 | 0.86±0.11 | 0.85±0.11 | 0.89±0.14 | 0.89±0.15 |
| *Neoromicia matroka* | 0.99±0 | 0.99±0.01 | 0.86±0.11 | 0.85±0.11 | 0.89±0.14 | 0.89±0.15 |
| *Neoromicia nanus* | 0.87±0.01 | 0.86±0.01 | 0.86±0.11 | 0.85±0.11 | 0.89±0.14 | 0.89±0.15 |
| *Neoromicia rendalli* | 0.90±0.03 | 0.91±0.03 | 0.86±0.11 | 0.85±0.11 | 0.89±0.14 | 0.89±0.15 |
| *Neoromicia somalica* | 0.89±0.02 | 0.85±0.03 | 0.86±0.11 | 0.85±0.11 | 0.90±0.14 | 0.89±0.15 |
| *Neoromicia stanleyi* | 0.97±0.01 | 0.96±0.02 | 0.86±0.11 | 0.85±0.11 | 0.90±0.13 | 0.89±0.15 |
| *Neoromicia tenuipinnis* | 0.94±0.01 | 0.94±0.01 | 0.86±0.11 | 0.85±0.11 | 0.90±0.13 | 0.89±0.15 |
| *Neoromicia zuluensis* | 0.90±0.02 | 0.89±0.02 | 0.86±0.11 | 0.84±0.11 | 0.90±0.13 | 0.89±0.15 |
| *Nycteris arge* | 0.95±0.01 | 0.94±0.01 | 0.86±0.11 | 0.84±0.11 | 0.90±0.13 | 0.90±0.15 |
| *Nycteris aurita* | 0.95±0.03 | 0.95±0.03 | 0.86±0.11 | 0.84±0.11 | 0.90±0.13 | 0.89±0.15 |
| *Nycteris gambiensis* | 0.97±0.01 | 0.95±0.02 | 0.86±0.11 | 0.85±0.11 | 0.90±0.13 | 0.89±0.14 |
| *Nycteris grandis* | 0.95±0.01 | 0.92±0.02 | 0.86±0.11 | 0.84±0.11 | 0.90±0.13 | 0.90±0.14 |
| *Nycteris hispida* | 0.88±0.01 | 0.88±0.01 | 0.85±0.11 | 0.84±0.11 | 0.90±0.13 | 0.90±0.14 |
| *Nycteris intermedia* | 0.93±0.03 | 0.89±0.05 | 0.85±0.11 | 0.84±0.11 | 0.90±0.13 | 0.90±0.14 |
| *Nycteris macrotis* | 0.87±0.02 | 0.87±0.02 | 0.85±0.11 | 0.84±0.11 | 0.90±0.13 | 0.90±0.14 |
| *Nycteris nana* | 0.94±0.02 | 0.94±0.02 | 0.85±0.11 | 0.84±0.11 | 0.90±0.13 | 0.90±0.14 |
| *Nycteris thebaica* | 0.85±0.01 | 0.85±0.01 | 0.85±0.11 | 0.84±0.12 | 0.90±0.13 | 0.90±0.14 |
| *Nycteris woodi* | 0.97±0.02 | 0.96±0.02 | 0.85±0.11 | 0.84±0.12 | 0.90±0.13 | 0.90±0.14 |
| *Nycticeinops schlieffenii* | 0.88±0.02 | 0.86±0.02 | 0.85±0.11 | 0.84±0.12 | 0.90±0.13 | 0.90±0.14 |
| *Otomops madagascariensis* | 0.99±0 | 0.99±0 | 0.85±0.11 | 0.84±0.12 | 0.90±0.13 | 0.90±0.14 |
| *Otomops martiensseni* | 0.95±0.03 | 0.90±0.05 | 0.85±0.11 | 0.84±0.12 | 0.90±0.13 | 0.90±0.14 |
| *Otonycteris hemprichii* | 0.98±0.01 | 0.98±0.01 | 0.85±0.11 | 0.84±0.12 | 0.90±0.13 | 0.90±0.14 |
| *Paremballonura atrata* | 0.99±0 | 0.99±0.01 | 0.85±0.11 | 0.84±0.12 | 0.90±0.14 | 0.90±0.15 |
| *Paremballonura tiavato* | 0.99±0 | 0.99±0 | 0.85±0.11 | 0.84±0.12 | 0.90±0.14 | 0.89±0.15 |
| *Pipistrellus hesperidus* | 0.94±0.02 | 0.93±0.01 | 0.85±0.11 | 0.84±0.12 | 0.90±0.14 | 0.90±0.15 |
| *Pipistrellus kuhlii* | 0.96±0.02 | 0.94±0.03 | 0.85±0.11 | 0.84±0.12 | 0.90±0.14 | 0.90±0.15 |
| *Pipistrellus nanulus* | 0.93±0.02 | 0.91±0.02 | 0.85±0.11 | 0.84±0.12 | 0.90±0.14 | 0.90±0.15 |
| *Pipistrellus rusticus* | 0.89±0.03 | 0.89±0.03 | 0.85±0.11 | 0.84±0.12 | 0.90±0.14 | 0.90±0.15 |
| *Platymops setiger* | 0.97±0.02 | 0.96±0.02 | 0.85±0.11 | 0.84±0.12 | 0.90±0.14 | 0.90±0.15 |
| *Plecotus christii* | 0.98±0.01 | 0.98±0.01 | 0.85±0.11 | 0.84±0.12 | 0.90±0.14 | 0.90±0.15 |
| *Plecotus teneriffae* | 0.99±0.01 | 0.97±0.02 | 0.85±0.11 | 0.84±0.12 | 0.90±0.14 | 0.90±0.15 |
| *Pteropus rufus* | 0.98±0.01 | 0.99±0.01 | 0.86±0.11 | 0.84±0.12 | 0.89±0.14 | 0.90±0.15 |
| *Pteropus seychellensis* | 0.98±0 | 0.99±0 | 0.86±0.11 | 0.84±0.12 | 0.90±0.14 | 0.89±0.15 |
| *Rhinolophus alcyone* | 0.94±0.02 | 0.94±0.02 | 0.86±0.11 | 0.85±0.12 | 0.90±0.14 | 0.89±0.15 |
| *Rhinolophus blasii* | 0.95±0.02 | 0.95±0.02 | 0.86±0.11 | 0.85±0.12 | 0.90±0.14 | 0.89±0.15 |
| *Rhinolophus capensis* | 0.99±0.01 | 0.98±0.01 | 0.86±0.11 | 0.85±0.12 | 0.89±0.14 | 0.89±0.15 |
| *Rhinolophus clivosus* | 0.93±0.01 | 0.92±0.01 | 0.86±0.11 | 0.85±0.12 | 0.89±0.14 | 0.89±0.15 |
| *Rhinolophus damarensis* | 0.97±0.02 | 0.95±0.03 | 0.86±0.11 | 0.85±0.12 | 0.89±0.14 | 0.89±0.15 |
| *Rhinolophus darlingi* | 0.97±0.01 | 0.96±0.01 | 0.86±0.11 | 0.85±0.12 | 0.89±0.14 | 0.89±0.15 |
| *Rhinolophus deckenii* | 0.97±0.01 | 0.95±0.02 | 0.86±0.11 | 0.85±0.12 | 0.89±0.14 | 0.89±0.15 |
| *Rhinolophus denti* | 0.90±0.06 | 0.91±0.04 | 0.86±0.11 | 0.85±0.12 | 0.89±0.14 | 0.89±0.15 |
| *Rhinolophus eloquens* | 0.94±0.03 | 0.95±0.02 | 0.86±0.11 | 0.85±0.12 | 0.89±0.14 | 0.89±0.15 |
| *Rhinolophus ferrumequinum* | 0.99±0.01 | 0.97±0.02 | 0.86±0.11 | 0.85±0.12 | 0.89±0.14 | 0.89±0.15 |
| *Rhinolophus fumigatus* | 0.85±0.02 | 0.84±0.02 | 0.86±0.11 | 0.85±0.12 | 0.89±0.14 | 0.90±0.15 |
| *Rhinolophus hildebrandtii* | 0.91±0.02 | 0.91±0.02 | 0.86±0.11 | 0.85±0.12 | 0.89±0.14 | 0.90±0.14 |
| *Rhinolophus hipposideros* | 0.97±0.02 | 0.98±0.02 | 0.86±0.11 | 0.85±0.12 | 0.89±0.14 | 0.90±0.14 |
| *Rhinolophus landeri* | 0.88±0.03 | 0.84±0.04 | 0.86±0.11 | 0.85±0.12 | 0.89±0.14 | 0.90±0.14 |
| *Rhinolophus lobatus* | 0.91±0.02 | 0.89±0.02 | 0.86±0.11 | 0.84±0.12 | 0.89±0.14 | 0.90±0.14 |
| *Rhinolophus mehelyi* | 0.99±0.01 | 0.98±0.01 | 0.86±0.11 | 0.85±0.12 | 0.89±0.14 | 0.89±0.14 |
| *Rhinolophus rhodesiae* | 0.96±0.02 | 0.92±0.02 | 0.86±0.11 | 0.85±0.12 | 0.89±0.14 | 0.89±0.14 |
| *Rhinolophus simulator* | 0.94±0.01 | 0.94±0.01 | 0.86±0.11 | 0.85±0.12 | 0.89±0.14 | 0.90±0.14 |
| *Rhinolophus smithersi* | 0.97±0.01 | 0.96±0.01 | 0.86±0.11 | 0.85±0.12 | 0.89±0.14 | 0.90±0.14 |
| *Rhinolophus swinnyi* | 0.99±0 | 0.99±0 | 0.86±0.11 | 0.85±0.12 | 0.89±0.14 | 0.90±0.14 |
| *Rhinopoma cystops* | 0.92±0.03 | 0.9±0.05 | 0.86±0.11 | 0.85±0.12 | 0.89±0.14 | 0.90±0.14 |
| *Rhinopoma microphyllum* | 0.95±0.04 | 0.96±0.02 | 0.86±0.11 | 0.85±0.12 | 0.89±0.14 | 0.90±0.14 |
| *Rousettus aegyptiacus* | 0.91±0.01 | 0.91±0.01 | 0.86±0.11 | 0.85±0.12 | 0.89±0.14 | 0.90±0.14 |
| *Rousettus madagascariensis* | 0.99±0 | 0.99±0 | 0.86±0.11 | 0.85±0.12 | 0.89±0.14 | 0.90±0.14 |
| *Rousettus obliviosus* | 0.99±0 | 0.99±0 | 0.86±0.11 | 0.85±0.12 | 0.89±0.14 | 0.90±0.14 |
| *Saccolaimus peli* | 0.96±0.01 | 0.94±0.03 | 0.86±0.11 | 0.85±0.12 | 0.89±0.14 | 0.90±0.14 |
| *Sauromys petrophilus* | 0.97±0.01 | 0.96±0.01 | 0.86±0.11 | 0.85±0.12 | 0.89±0.14 | 0.90±0.14 |
| *Scotoecus albofuscus* | 0.93±0.03 | 0.93±0.04 | 0.86±0.11 | 0.85±0.12 | 0.89±0.14 | 0.90±0.14 |
| *Scotoecus hirundo* | 0.89±0.02 | 0.88±0.03 | 0.86±0.11 | 0.85±0.12 | 0.89±0.14 | 0.90±0.14 |
| *Scotonycteris bergmansi* | 0.96±0.01 | 0.96±0.02 | 0.86±0.11 | 0.85±0.12 | 0.89±0.14 | 0.90±0.14 |
| *Scotonycteris occidentalis* | 0.98±0.01 | 0.97±0.01 | 0.86±0.11 | 0.85±0.12 | 0.89±0.14 | 0.90±0.14 |
| *Scotonycteris zenkeri* | 0.99±0.01 | 0.97±0.02 | 0.86±0.11 | 0.85±0.12 | 0.89±0.14 | 0.89±0.14 |
| *Scotophilus dinganii* | 0.90±0.01 | 0.89±0.01 | 0.86±0.11 | 0.85±0.12 | 0.89±0.14 | 0.9±0.14 |
| *Scotophilus leucogaster* | 0.88±0.03 | 0.90±0.02 | 0.86±0.11 | 0.85±0.12 | 0.89±0.14 | 0.9±0.14 |
| *Scotophilus nigrita* | 0.90±0.04 | 0.88±0.05 | 0.86±0.11 | 0.85±0.12 | 0.89±0.14 | 0.9±0.14 |
| *Scotophilus nigritellus* | 0.97±0.01 | 0.95±0.02 | 0.86±0.11 | 0.85±0.12 | 0.89±0.14 | 0.9±0.14 |
| *Scotophilus nux* | 0.95±0.02 | 0.96±0.01 | 0.86±0.11 | 0.85±0.12 | 0.89±0.14 | 0.9±0.14 |
| *Scotophilus robustus* | 0.99±0.01 | 0.97±0.01 | 0.86±0.11 | 0.85±0.12 | 0.89±0.14 | 0.9±0.14 |
| *Scotophilus viridis* | 0.93±0.02 | 0.93±0.02 | 0.86±0.11 | 0.85±0.12 | 0.89±0.14 | 0.9±0.14 |
| *Stenonycteris lanosus* | 0.98±0.01 | 0.98±0.01 | 0.86±0.11 | 0.85±0.12 | 0.89±0.14 | 0.9±0.14 |
| *Tadarida aegyptiaca* | 0.91±0.01 | 0.91±0.01 | 0.86±0.11 | 0.85±0.12 | 0.89±0.14 | 0.9±0.14 |
| *Tadarida fulminans* | 0.93±0.03 | 0.93±0.03 | 0.86±0.11 | 0.85±0.12 | 0.89±0.14 | 0.9±0.14 |
| *Tadarida lobata* | 0.92±0.04 | 0.91±0.05 | 0.86±0.11 | 0.85±0.12 | 0.89±0.14 | 0.9±0.14 |
| *Tadarida ventralis* | 0.88±0.06 | 0.85±0.07 | 0.86±0.11 | 0.85±0.12 | 0.89±0.14 | 0.9±0.14 |
| *Taphozous mauritianus* | 0.89±0.02 | 0.89±0.02 | 0.86±0.11 | 0.85±0.12 | 0.89±0.14 | 0.9±0.14 |
| *Taphozous nudiventris* | 0.91±0.05 | 0.88±0.06 | 0.86±0.11 | 0.85±0.12 | 0.89±0.14 | 0.9±0.14 |
| *Taphozous perforatus* | 0.85±0.04 | 0.84±0.04 | 0.86±0.11 | 0.85±0.12 | 0.89±0.14 | 0.9±0.14 |
| *Triaenops afer* | 0.91±0.03 | 0.89±0.04 | 0.86±0.11 | 0.84±0.12 | 0.89±0.14 | 0.9±0.14 |
| *Triaenops menamena* | 0.99±0 | 0.99±0.01 | 0.86±0.11 | 0.85±0.12 | 0.89±0.14 | 0.9±0.14 |
| *Vansonia rueppellii* | 0.90±0.03 | 0.88±0.03 | 0.86±0.11 | 0.84±0.12 | 0.89±0.14 | 0.9±0.14 |

| Species | AUC_TR | AUC_noTR | TSS_TR | TSS_noTR | Boyce_TR | Boyce_noTR |
| --- | --- | --- | --- | --- | --- | --- |
| *Asellia tridens* | 0.869±0.052 | 0.847±0.067 | 0.783±0.069 | 0.711±0.06 | 0.929±0.046 | 0.896±0.111 |
| *Cardioderma cor* | 0.929±0.03 | 0.887±0.038 | 0.79±0.078 | 0.706±0.06 | 0.912±0.059 | 0.873±0.104 |
| *Casinycteris argynnis* | 0.965±0.016 | 0.962±0.015 | 0.842±0.1 | 0.773±0.109 | 0.862±0.171 | 0.837±0.143 |
| *Chaerephon aloysiisabaudiae* | 0.942±0.023 | 0.961±0.017 | 0.867±0.098 | 0.816±0.121 | 0.882±0.155 | 0.844±0.133 |
| *Chaerephon ansorgei* | 0.886±0.033 | 0.844±0.041 | 0.837±0.113 | 0.802±0.113 | 0.888±0.143 | 0.865±0.128 |
| *Chaerephon atsinanana* | 0.997±0.001 | 0.996±0.003 | 0.864±0.119 | 0.835±0.126 | 0.883±0.142 | 0.861±0.121 |
| *Chaerephon bivittatus* | 0.897±0.035 | 0.911±0.032 | 0.851±0.116 | 0.824±0.121 | 0.891±0.134 | 0.867±0.116 |
| *Chaerephon chapini* | 0.773±0.095 | 0.826±0.068 | 0.832±0.126 | 0.822±0.119 | 0.894±0.126 | 0.874±0.113 |
| *Chaerephon leucogaster* | 0.935±0.03 | 0.939±0.031 | 0.836±0.12 | 0.829±0.115 | 0.896±0.12 | 0.875±0.108 |
| *Chaerephon major* | 0.884±0.04 | 0.851±0.052 | 0.834±0.115 | 0.821±0.115 | 0.889±0.12 | 0.872±0.111 |
| *Chaerephon nigeriae* | 0.862±0.037 | 0.822±0.045 | 0.819±0.123 | 0.806±0.119 | 0.885±0.119 | 0.87±0.113 |
| *Chaerephon pumilus* | 0.903±0.01 | 0.898±0.01 | 0.809±0.121 | 0.797±0.118 | 0.894±0.118 | 0.881±0.114 |
| *Chaerephon pusillus* | 0.973±0.024 | 0.938±0.044 | 0.819±0.123 | 0.809±0.123 | 0.897±0.114 | 0.883±0.111 |
| *Cistugo lesueuri* | 0.991±0.004 | 0.993±0.004 | 0.831±0.127 | 0.822±0.127 | 0.895±0.113 | 0.883±0.108 |
| *Cloeotis percivali* | 0.959±0.018 | 0.941±0.028 | 0.838±0.125 | 0.829±0.126 | 0.896±0.11 | 0.887±0.107 |
| *Coleura afra* | 0.903±0.029 | 0.892±0.032 | 0.83±0.125 | 0.822±0.126 | 0.894±0.108 | 0.884±0.108 |
| *Doryrhina cyclops* | 0.95±0.014 | 0.941±0.015 | 0.829±0.122 | 0.824±0.123 | 0.899±0.106 | 0.89±0.108 |
| *Eidolon dupreanum* | 0.962±0.016 | 0.979±0.01 | 0.837±0.123 | 0.832±0.124 | 0.898±0.106 | 0.887±0.111 |
| *Eidolon helvum* | 0.887±0.016 | 0.887±0.016 | 0.828±0.127 | 0.824±0.126 | 0.903±0.106 | 0.893±0.11 |
| *Epomophorus angolensis* | 0.965±0.017 | 0.95±0.019 | 0.833±0.126 | 0.83±0.127 | 0.903±0.106 | 0.887±0.119 |
| *Epomophorus crypturus* | 0.936±0.014 | 0.931±0.016 | 0.83±0.124 | 0.827±0.125 | 0.906±0.104 | 0.892±0.118 |
| *Epomophorus dobsonii* | 0.969±0.013 | 0.951±0.017 | 0.833±0.122 | 0.831±0.123 | 0.907±0.103 | 0.889±0.119 |
| *Epomophorus gambianus* | 0.926±0.012 | 0.925±0.012 | 0.832±0.12 | 0.828±0.122 | 0.911±0.102 | 0.894±0.118 |
| *Epomophorus labiatus* | 0.919±0.012 | 0.893±0.015 | 0.828±0.119 | 0.821±0.124 | 0.914±0.101 | 0.898±0.117 |
| *Epomophorus minor* | 0.909±0.025 | 0.882±0.037 | 0.827±0.117 | 0.818±0.122 | 0.914±0.101 | 0.899±0.116 |
| *Epomophorus pusillus* | 0.928±0.009 | 0.92±0.011 | 0.825±0.115 | 0.816±0.12 | 0.917±0.101 | 0.903±0.115 |
| *Epomophorus wahlbergi* | 0.921±0.01 | 0.909±0.012 | 0.823±0.114 | 0.813±0.12 | 0.92±0.1 | 0.906±0.114 |
| *Epomops buettikoferi* | 0.975±0.011 | 0.97±0.011 | 0.828±0.114 | 0.816±0.119 | 0.92±0.098 | 0.907±0.112 |
| *Epomops franqueti* | 0.945±0.006 | 0.933±0.009 | 0.828±0.112 | 0.816±0.117 | 0.923±0.098 | 0.91±0.111 |
| *Eptesicus hottentotus* | 0.934±0.024 | 0.928±0.028 | 0.828±0.111 | 0.817±0.116 | 0.923±0.097 | 0.911±0.111 |
| *Eptesicus isabellinus* | 0.983±0.005 | 0.978±0.01 | 0.833±0.113 | 0.822±0.117 | 0.92±0.104 | 0.912±0.111 |
| *Glauconycteris argentata* | 0.937±0.027 | 0.935±0.029 | 0.834±0.111 | 0.823±0.115 | 0.918±0.104 | 0.913±0.109 |
| *Glauconycteris beatrix* | 0.939±0.027 | 0.933±0.026 | 0.836±0.11 | 0.825±0.115 | 0.913±0.112 | 0.905±0.128 |
| *Glauconycteris poensis* | 0.945±0.026 | 0.932±0.043 | 0.838±0.11 | 0.827±0.114 | 0.913±0.111 | 0.905±0.127 |
| *Glauconycteris variegata* | 0.886±0.032 | 0.867±0.037 | 0.835±0.11 | 0.823±0.115 | 0.913±0.11 | 0.905±0.127 |
| *Hipposideros abae* | 0.931±0.017 | 0.917±0.028 | 0.837±0.109 | 0.825±0.114 | 0.914±0.109 | 0.904±0.127 |
| *Hipposideros beatus* | 0.954±0.017 | 0.955±0.015 | 0.838±0.108 | 0.825±0.113 | 0.914±0.108 | 0.904±0.126 |
| *Hipposideros caffer* | 0.878±0.012 | 0.868±0.013 | 0.832±0.111 | 0.82±0.116 | 0.916±0.107 | 0.906±0.125 |
| *Hipposideros fuliginosus* | 0.946±0.023 | 0.937±0.026 | 0.834±0.111 | 0.822±0.115 | 0.916±0.107 | 0.907±0.124 |
| *Hipposideros jonesi* | 0.953±0.018 | 0.895±0.04 | 0.836±0.11 | 0.824±0.115 | 0.914±0.107 | 0.9±0.139 |
| *Hipposideros ruber* | 0.903±0.012 | 0.869±0.015 | 0.832±0.111 | 0.82±0.116 | 0.916±0.107 | 0.903±0.138 |
| *Hipposideros tephrus* | 0.881±0.033 | 0.883±0.035 | 0.831±0.11 | 0.819±0.115 | 0.916±0.106 | 0.903±0.137 |
| *Hypsignathus monstrosus* | 0.95±0.012 | 0.951±0.01 | 0.831±0.109 | 0.819±0.114 | 0.917±0.105 | 0.904±0.136 |
| *Hypsugo anchieta* | 0.948±0.022 | 0.929±0.028 | 0.831±0.108 | 0.819±0.113 | 0.918±0.104 | 0.904±0.135 |
| *Hypsugo crassulus* | 0.933±0.021 | 0.928±0.024 | 0.833±0.107 | 0.822±0.113 | 0.914±0.111 | 0.904±0.135 |
| *Kerivoula argentata* | 0.95±0.024 | 0.927±0.031 | 0.834±0.107 | 0.822±0.112 | 0.915±0.11 | 0.903±0.134 |
| *Kerivoula lanosa* | 0.896±0.039 | 0.904±0.034 | 0.833±0.106 | 0.821±0.112 | 0.915±0.11 | 0.903±0.133 |
| *Laephotis botswanae* | 0.943±0.016 | 0.939±0.018 | 0.835±0.105 | 0.823±0.112 | 0.913±0.114 | 0.895±0.153 |
| *Laephotis wintoni* | 0.968±0.019 | 0.922±0.039 | 0.838±0.106 | 0.825±0.112 | 0.914±0.113 | 0.895±0.152 |
| *Lavia frons* | 0.895±0.019 | 0.893±0.02 | 0.835±0.107 | 0.822±0.113 | 0.915±0.112 | 0.897±0.151 |
| *Lissonycteris angolensis* | 0.939±0.01 | 0.918±0.013 | 0.833±0.107 | 0.821±0.112 | 0.917±0.111 | 0.899±0.15 |
| *Macronycteris commersonii* | 0.989±0.004 | 0.985±0.004 | 0.836±0.107 | 0.823±0.113 | 0.917±0.111 | 0.9±0.149 |
| *Macronycteris gigas* | 0.917±0.027 | 0.901±0.032 | 0.836±0.106 | 0.822±0.112 | 0.915±0.111 | 0.897±0.149 |
| *Macronycteris vittatus* | 0.889±0.021 | 0.874±0.024 | 0.833±0.108 | 0.82±0.113 | 0.916±0.111 | 0.898±0.148 |
| *Megaloglossus azagnyi* | 0.986±0.004 | 0.982±0.005 | 0.835±0.108 | 0.822±0.113 | 0.914±0.113 | 0.897±0.148 |
| *Megaloglossus woermanni* | 0.957±0.01 | 0.957±0.009 | 0.836±0.107 | 0.823±0.112 | 0.914±0.113 | 0.898±0.147 |
| *Mimetillus moloneyi* | 0.922±0.025 | 0.923±0.023 | 0.835±0.107 | 0.823±0.112 | 0.914±0.112 | 0.899±0.146 |
| *Miniopterus aelleni* | 0.976±0.012 | 0.983±0.009 | 0.838±0.107 | 0.826±0.113 | 0.911±0.116 | 0.899±0.145 |
| *Miniopterus africanus* | 0.938±0.028 | 0.94±0.026 | 0.84±0.107 | 0.827±0.113 | 0.91±0.115 | 0.899±0.144 |
| *Miniopterus arenarius* | 0.963±0.01 | 0.942±0.024 | 0.84±0.107 | 0.828±0.112 | 0.909±0.116 | 0.899±0.143 |
| *Miniopterus fraterculus* | 0.946±0.019 | 0.942±0.019 | 0.84±0.106 | 0.827±0.111 | 0.908±0.116 | 0.899±0.143 |
| *Miniopterus gleni* | 0.994±0.002 | 0.991±0.003 | 0.842±0.107 | 0.83±0.112 | 0.906±0.118 | 0.899±0.142 |
| *Miniopterus griveaudi* | 0.994±0.003 | 0.994±0.002 | 0.845±0.108 | 0.832±0.113 | 0.906±0.117 | 0.898±0.143 |
| *Miniopterus inflatus* | 0.889±0.031 | 0.908±0.026 | 0.844±0.107 | 0.83±0.113 | 0.907±0.117 | 0.898±0.142 |
| *Miniopterus mahafaliensis* | 0.995±0.003 | 0.989±0.006 | 0.846±0.108 | 0.833±0.114 | 0.906±0.117 | 0.896±0.144 |
| *Miniopterus majori* | 0.983±0.007 | 0.958±0.025 | 0.848±0.109 | 0.835±0.115 | 0.904±0.118 | 0.894±0.147 |
| *Miniopterus manavi* | 0.982±0.007 | 0.978±0.012 | 0.85±0.109 | 0.837±0.115 | 0.901±0.126 | 0.894±0.147 |
| *Miniopterus minor* | 0.925±0.037 | 0.919±0.043 | 0.851±0.109 | 0.838±0.114 | 0.901±0.126 | 0.894±0.147 |
| *Miniopterus mossambicus* | 0.966±0.014 | 0.966±0.014 | 0.852±0.109 | 0.839±0.114 | 0.9±0.126 | 0.892±0.149 |
| *Miniopterus natalensis* | 0.92±0.01 | 0.906±0.011 | 0.85±0.109 | 0.838±0.114 | 0.901±0.125 | 0.893±0.149 |
| *Miniopterus schreibersii* | 0.994±0.002 | 0.991±0.003 | 0.852±0.11 | 0.84±0.115 | 0.896±0.138 | 0.893±0.148 |
| *Mops brachypterus* | 0.928±0.038 | 0.922±0.037 | 0.852±0.109 | 0.84±0.114 | 0.896±0.139 | 0.892±0.148 |
| *Mops condylurus* | 0.904±0.013 | 0.907±0.014 | 0.85±0.11 | 0.838±0.115 | 0.897±0.138 | 0.894±0.147 |
| *Mops demonstrator* | 0.886±0.045 | 0.897±0.041 | 0.851±0.109 | 0.84±0.114 | 0.896±0.139 | 0.893±0.147 |
| *Mops leucostigma* | 0.989±0.007 | 0.99±0.006 | 0.853±0.11 | 0.842±0.115 | 0.896±0.138 | 0.893±0.146 |
| *Mops midas* | 0.904±0.035 | 0.875±0.037 | 0.851±0.111 | 0.84±0.116 | 0.896±0.138 | 0.893±0.146 |
| *Mops nanulus* | 0.956±0.017 | 0.949±0.022 | 0.851±0.11 | 0.84±0.115 | 0.896±0.137 | 0.892±0.146 |
| *Mops niveiventer* | 0.967±0.017 | 0.962±0.018 | 0.852±0.11 | 0.841±0.115 | 0.896±0.137 | 0.892±0.145 |
| *Mops spurrelli* | 0.976±0.011 | 0.967±0.015 | 0.854±0.11 | 0.842±0.115 | 0.896±0.136 | 0.893±0.144 |
| *Mops thersites* | 0.946±0.022 | 0.949±0.017 | 0.854±0.109 | 0.842±0.114 | 0.895±0.136 | 0.891±0.147 |
| *Mormopterus jugularis* | 0.994±0.002 | 0.981±0.007 | 0.855±0.109 | 0.844±0.114 | 0.894±0.136 | 0.892±0.146 |
| *Myonycteris leptodon* | 0.984±0.005 | 0.975±0.009 | 0.857±0.109 | 0.845±0.114 | 0.894±0.137 | 0.892±0.146 |
| *Myonycteris torquata* | 0.94±0.015 | 0.93±0.019 | 0.856±0.109 | 0.845±0.114 | 0.895±0.136 | 0.893±0.145 |
| *Myotis bocagii* | 0.919±0.014 | 0.909±0.017 | 0.855±0.109 | 0.843±0.114 | 0.896±0.136 | 0.894±0.145 |
| *Myotis goudotii* | 0.989±0.002 | 0.984±0.004 | 0.856±0.109 | 0.845±0.114 | 0.896±0.135 | 0.893±0.144 |
| *Myotis punicus* | 0.992±0.002 | 0.99±0.003 | 0.858±0.109 | 0.846±0.114 | 0.895±0.135 | 0.893±0.144 |
| *Myotis tricolor* | 0.957±0.011 | 0.956±0.01 | 0.858±0.109 | 0.846±0.113 | 0.896±0.135 | 0.894±0.143 |
| *Myotis welwitschii* | 0.944±0.02 | 0.933±0.024 | 0.858±0.108 | 0.846±0.113 | 0.895±0.135 | 0.894±0.143 |
| *Myzopoda aurita* | 0.995±0.003 | 0.991±0.006 | 0.86±0.109 | 0.848±0.113 | 0.892±0.139 | 0.89±0.151 |
| *Nanonycteris veldkampii* | 0.979±0.007 | 0.96±0.013 | 0.86±0.108 | 0.848±0.113 | 0.893±0.139 | 0.89±0.15 |
| *Neoromicia brunnea* | 0.938±0.038 | 0.948±0.028 | 0.861±0.108 | 0.849±0.113 | 0.893±0.138 | 0.891±0.15 |
| *Neoromicia capensis* | 0.909±0.008 | 0.905±0.009 | 0.859±0.108 | 0.848±0.113 | 0.894±0.138 | 0.892±0.149 |
| *Neoromicia grandidieri* | 0.954±0.03 | 0.925±0.048 | 0.86±0.108 | 0.849±0.113 | 0.894±0.138 | 0.891±0.15 |
| *Neoromicia guineensis* | 0.914±0.038 | 0.886±0.048 | 0.86±0.108 | 0.848±0.113 | 0.894±0.137 | 0.892±0.149 |
| *Neoromicia helios* | 0.916±0.041 | 0.905±0.038 | 0.86±0.108 | 0.848±0.112 | 0.893±0.137 | 0.892±0.149 |
| *Neoromicia matroka* | 0.991±0.004 | 0.988±0.006 | 0.861±0.108 | 0.85±0.113 | 0.893±0.136 | 0.891±0.149 |
| *Neoromicia nanus* | 0.87±0.011 | 0.865±0.011 | 0.859±0.11 | 0.847±0.114 | 0.894±0.136 | 0.892±0.148 |
| *Neoromicia rendalli* | 0.896±0.026 | 0.91±0.026 | 0.858±0.11 | 0.847±0.114 | 0.894±0.136 | 0.893±0.148 |
| *Neoromicia somalica* | 0.889±0.021 | 0.851±0.028 | 0.857±0.109 | 0.845±0.114 | 0.895±0.135 | 0.894±0.147 |
| *Neoromicia stanleyi* | 0.972±0.014 | 0.957±0.018 | 0.858±0.109 | 0.846±0.114 | 0.895±0.135 | 0.893±0.147 |
| *Neoromicia tenuipinnis* | 0.945±0.014 | 0.945±0.014 | 0.858±0.109 | 0.846±0.114 | 0.896±0.134 | 0.894±0.146 |
| *Neoromicia zuluensis* | 0.897±0.024 | 0.894±0.022 | 0.856±0.109 | 0.845±0.114 | 0.896±0.134 | 0.895±0.146 |
| *Nycteris arge* | 0.946±0.014 | 0.945±0.014 | 0.856±0.109 | 0.845±0.113 | 0.897±0.133 | 0.895±0.145 |
| *Nycteris aurita* | 0.946±0.027 | 0.945±0.028 | 0.856±0.108 | 0.845±0.113 | 0.897±0.133 | 0.895±0.145 |
| *Nycteris gambiensis* | 0.968±0.012 | 0.947±0.023 | 0.857±0.108 | 0.845±0.112 | 0.898±0.132 | 0.895±0.145 |
| *Nycteris grandis* | 0.946±0.013 | 0.924±0.016 | 0.856±0.107 | 0.844±0.112 | 0.898±0.132 | 0.896±0.144 |
| *Nycteris hispida* | 0.885±0.012 | 0.882±0.013 | 0.854±0.109 | 0.842±0.113 | 0.899±0.132 | 0.897±0.144 |
| *Nycteris intermedia* | 0.935±0.027 | 0.894±0.054 | 0.854±0.109 | 0.843±0.114 | 0.899±0.131 | 0.897±0.144 |
| *Nycteris macrotis* | 0.868±0.018 | 0.866±0.018 | 0.853±0.11 | 0.841±0.115 | 0.9±0.131 | 0.898±0.143 |
| *Nycteris nana* | 0.943±0.02 | 0.935±0.023 | 0.853±0.11 | 0.841±0.114 | 0.899±0.131 | 0.898±0.143 |
| *Nycteris thebaica* | 0.854±0.012 | 0.845±0.013 | 0.851±0.112 | 0.839±0.116 | 0.9±0.131 | 0.899±0.143 |
| *Nycteris woodi* | 0.969±0.019 | 0.962±0.021 | 0.851±0.112 | 0.839±0.116 | 0.901±0.13 | 0.898±0.142 |
| *Nycticeinops schlieffenii* | 0.88±0.021 | 0.863±0.022 | 0.85±0.113 | 0.838±0.117 | 0.901±0.13 | 0.899±0.142 |
| *Otomops madagascariensis* | 0.997±0.001 | 0.994±0.002 | 0.851±0.113 | 0.839±0.117 | 0.9±0.131 | 0.898±0.142 |
| *Otomops martiensseni* | 0.946±0.032 | 0.897±0.05 | 0.851±0.113 | 0.838±0.117 | 0.9±0.131 | 0.899±0.142 |
| *Otonycteris hemprichii* | 0.976±0.015 | 0.977±0.014 | 0.852±0.113 | 0.84±0.117 | 0.899±0.132 | 0.898±0.142 |
| *Paremballonura atrata* | 0.996±0.003 | 0.992±0.005 | 0.853±0.113 | 0.841±0.117 | 0.896±0.139 | 0.895±0.151 |
| *Paremballonura tiavato* | 0.996±0.003 | 0.997±0.001 | 0.854±0.113 | 0.842±0.118 | 0.895±0.14 | 0.895±0.15 |
| *Pipistrellus hesperidus* | 0.935±0.015 | 0.933±0.015 | 0.854±0.113 | 0.841±0.118 | 0.896±0.14 | 0.895±0.15 |
| *Pipistrellus kuhlii* | 0.96±0.016 | 0.942±0.025 | 0.854±0.113 | 0.842±0.117 | 0.895±0.14 | 0.895±0.149 |
| *Pipistrellus nanulus* | 0.932±0.022 | 0.914±0.025 | 0.853±0.112 | 0.841±0.117 | 0.896±0.139 | 0.896±0.149 |
| *Pipistrellus rusticus* | 0.887±0.032 | 0.889±0.034 | 0.852±0.113 | 0.84±0.118 | 0.896±0.139 | 0.896±0.148 |
| *Platymops setiger* | 0.966±0.017 | 0.961±0.015 | 0.852±0.113 | 0.841±0.117 | 0.896±0.138 | 0.896±0.148 |
| *Plecotus christii* | 0.976±0.012 | 0.981±0.013 | 0.853±0.113 | 0.842±0.118 | 0.896±0.138 | 0.896±0.147 |
| *Plecotus teneriffae* | 0.989±0.006 | 0.972±0.018 | 0.855±0.113 | 0.843±0.118 | 0.895±0.138 | 0.896±0.147 |
| *Pteropus rufus* | 0.975±0.012 | 0.986±0.006 | 0.855±0.113 | 0.844±0.118 | 0.895±0.139 | 0.895±0.147 |
| *Pteropus seychellensis* | 0.981±0.004 | 0.997±0.002 | 0.856±0.113 | 0.845±0.118 | 0.895±0.138 | 0.895±0.148 |
| *Rhinolophus alcyone* | 0.942±0.024 | 0.941±0.022 | 0.856±0.113 | 0.845±0.118 | 0.895±0.138 | 0.894±0.147 |
| *Rhinolophus blasii* | 0.946±0.016 | 0.947±0.017 | 0.856±0.113 | 0.845±0.117 | 0.895±0.138 | 0.894±0.147 |
| *Rhinolophus capensis* | 0.986±0.011 | 0.985±0.009 | 0.857±0.113 | 0.846±0.118 | 0.894±0.139 | 0.894±0.147 |
| *Rhinolophus clivosus* | 0.927±0.012 | 0.921±0.011 | 0.857±0.112 | 0.846±0.117 | 0.895±0.139 | 0.895±0.146 |
| *Rhinolophus damarensis* | 0.968±0.019 | 0.95±0.029 | 0.857±0.112 | 0.846±0.117 | 0.894±0.14 | 0.894±0.147 |
| *Rhinolophus darlingi* | 0.973±0.008 | 0.96±0.01 | 0.857±0.112 | 0.846±0.117 | 0.895±0.139 | 0.895±0.146 |
| *Rhinolophus deckenii* | 0.972±0.015 | 0.951±0.023 | 0.858±0.112 | 0.847±0.116 | 0.894±0.14 | 0.894±0.146 |
| *Rhinolophus denti* | 0.899±0.061 | 0.914±0.04 | 0.858±0.111 | 0.847±0.116 | 0.894±0.14 | 0.894±0.146 |
| *Rhinolophus eloquens* | 0.944±0.026 | 0.949±0.022 | 0.858±0.111 | 0.847±0.116 | 0.894±0.14 | 0.894±0.146 |
| *Rhinolophus ferrumequinum* | 0.988±0.006 | 0.974±0.017 | 0.859±0.111 | 0.848±0.116 | 0.894±0.139 | 0.894±0.145 |
| *Rhinolophus fumigatus* | 0.851±0.02 | 0.845±0.022 | 0.858±0.112 | 0.846±0.117 | 0.894±0.139 | 0.895±0.145 |
| *Rhinolophus hildebrandtii* | 0.914±0.023 | 0.912±0.025 | 0.857±0.112 | 0.846±0.117 | 0.895±0.139 | 0.895±0.145 |
| *Rhinolophus hipposideros* | 0.967±0.022 | 0.977±0.017 | 0.858±0.112 | 0.847±0.117 | 0.894±0.139 | 0.895±0.144 |
| *Rhinolophus landeri* | 0.878±0.035 | 0.838±0.042 | 0.857±0.113 | 0.846±0.118 | 0.895±0.138 | 0.895±0.144 |
| *Rhinolophus lobatus* | 0.909±0.021 | 0.89±0.023 | 0.856±0.113 | 0.845±0.118 | 0.895±0.138 | 0.896±0.144 |
| *Rhinolophus mehelyi* | 0.988±0.006 | 0.982±0.011 | 0.857±0.113 | 0.846±0.118 | 0.894±0.139 | 0.895±0.144 |
| *Rhinolophus rhodesiae* | 0.955±0.016 | 0.92±0.025 | 0.857±0.112 | 0.845±0.118 | 0.894±0.139 | 0.895±0.144 |
| *Rhinolophus simulator* | 0.945±0.012 | 0.944±0.012 | 0.857±0.112 | 0.845±0.117 | 0.894±0.139 | 0.895±0.144 |
| *Rhinolophus smithersi* | 0.97±0.006 | 0.964±0.007 | 0.857±0.112 | 0.845±0.117 | 0.895±0.138 | 0.895±0.144 |
| *Rhinolophus swinnyi* | 0.995±0.002 | 0.994±0.003 | 0.858±0.112 | 0.846±0.117 | 0.893±0.143 | 0.895±0.143 |
| *Rhinopoma cystops* | 0.922±0.032 | 0.898±0.046 | 0.858±0.112 | 0.845±0.117 | 0.893±0.143 | 0.895±0.143 |
| *Rhinopoma microphyllum* | 0.946±0.036 | 0.96±0.024 | 0.858±0.112 | 0.846±0.117 | 0.893±0.143 | 0.895±0.143 |
| *Rousettus aegyptiacus* | 0.909±0.012 | 0.906±0.013 | 0.857±0.112 | 0.845±0.117 | 0.894±0.143 | 0.896±0.142 |
| *Rousettus madagascariensis* | 0.994±0.003 | 0.994±0.003 | 0.858±0.112 | 0.846±0.117 | 0.893±0.143 | 0.896±0.142 |
| *Rousettus obliviosus* | 0.999±0 | 0.999±0 | 0.859±0.112 | 0.847±0.118 | 0.893±0.143 | 0.896±0.142 |
| *Saccolaimus peli* | 0.959±0.015 | 0.94±0.028 | 0.86±0.112 | 0.848±0.118 | 0.893±0.143 | 0.896±0.141 |
| *Sauromys petrophilus* | 0.97±0.008 | 0.96±0.011 | 0.86±0.112 | 0.848±0.117 | 0.893±0.143 | 0.896±0.141 |
| *Scotoecus albofuscus* | 0.931±0.032 | 0.927±0.045 | 0.86±0.112 | 0.848±0.117 | 0.894±0.143 | 0.896±0.141 |
| *Scotoecus hirundo* | 0.893±0.024 | 0.878±0.026 | 0.86±0.112 | 0.847±0.117 | 0.894±0.142 | 0.897±0.14 |
| *Scotonycteris bergmansi* | 0.963±0.014 | 0.961±0.016 | 0.86±0.111 | 0.848±0.117 | 0.893±0.143 | 0.896±0.143 |
| *Scotonycteris occidentalis* | 0.976±0.009 | 0.966±0.011 | 0.861±0.111 | 0.848±0.117 | 0.893±0.143 | 0.895±0.143 |
| *Scotonycteris zenkeri* | 0.985±0.008 | 0.973±0.015 | 0.862±0.111 | 0.849±0.117 | 0.894±0.142 | 0.895±0.143 |
| *Scotophilus dinganii* | 0.904±0.011 | 0.893±0.012 | 0.861±0.112 | 0.848±0.117 | 0.894±0.142 | 0.896±0.143 |
| *Scotophilus leucogaster* | 0.877±0.026 | 0.899±0.023 | 0.86±0.113 | 0.847±0.118 | 0.895±0.142 | 0.896±0.142 |
| *Scotophilus nigrita* | 0.896±0.045 | 0.88±0.052 | 0.859±0.113 | 0.846±0.118 | 0.895±0.141 | 0.896±0.142 |
| *Scotophilus nigritellus* | 0.968±0.01 | 0.946±0.024 | 0.86±0.112 | 0.847±0.117 | 0.894±0.142 | 0.896±0.142 |
| *Scotophilus nux* | 0.954±0.018 | 0.957±0.014 | 0.86±0.112 | 0.847±0.117 | 0.893±0.142 | 0.896±0.142 |
| *Scotophilus robustus* | 0.987±0.005 | 0.971±0.013 | 0.861±0.112 | 0.848±0.117 | 0.893±0.142 | 0.896±0.142 |
| *Scotophilus viridis* | 0.926±0.024 | 0.933±0.024 | 0.86±0.112 | 0.847±0.117 | 0.893±0.142 | 0.896±0.141 |
| *Stenonycteris lanosus* | 0.981±0.009 | 0.976±0.012 | 0.861±0.112 | 0.848±0.117 | 0.893±0.142 | 0.896±0.141 |
| *Tadarida aegyptiaca* | 0.909±0.015 | 0.905±0.015 | 0.86±0.112 | 0.847±0.117 | 0.893±0.141 | 0.897±0.141 |
| *Tadarida fulminans* | 0.933±0.028 | 0.934±0.027 | 0.86±0.112 | 0.847±0.117 | 0.893±0.141 | 0.896±0.141 |
| *Tadarida lobata* | 0.918±0.043 | 0.909±0.051 | 0.86±0.112 | 0.848±0.117 | 0.894±0.141 | 0.897±0.14 |
| *Tadarida ventralis* | 0.875±0.056 | 0.85±0.068 | 0.86±0.111 | 0.847±0.116 | 0.893±0.141 | 0.896±0.141 |
| *Taphozous mauritianus* | 0.889±0.017 | 0.891±0.018 | 0.859±0.112 | 0.847±0.117 | 0.894±0.141 | 0.897±0.141 |
| *Taphozous nudiventris* | 0.91±0.051 | 0.876±0.061 | 0.859±0.112 | 0.846±0.117 | 0.894±0.141 | 0.897±0.141 |
| *Taphozous perforatus* | 0.852±0.039 | 0.842±0.044 | 0.858±0.113 | 0.845±0.117 | 0.894±0.141 | 0.897±0.14 |
| *Triaenops afer* | 0.913±0.03 | 0.893±0.037 | 0.857±0.113 | 0.845±0.117 | 0.893±0.142 | 0.896±0.141 |
| *Triaenops menamena* | 0.99±0.005 | 0.986±0.006 | 0.858±0.113 | 0.845±0.117 | 0.893±0.142 | 0.896±0.14 |
| *Vansonia rueppellii* | 0.9±0.027 | 0.88±0.032 | 0.857±0.113 | 0.845±0.117 | 0.893±0.141 | 0.896±0.14 |

Figure S3.10. AUC, TSS, and Boyce indicators for models calibrated with (“BIO-SDMs”, red) and without the trophic resource variable (“noBIO-SDMs”, blue) for frugivore (left) and insectivore (right) bats.

**
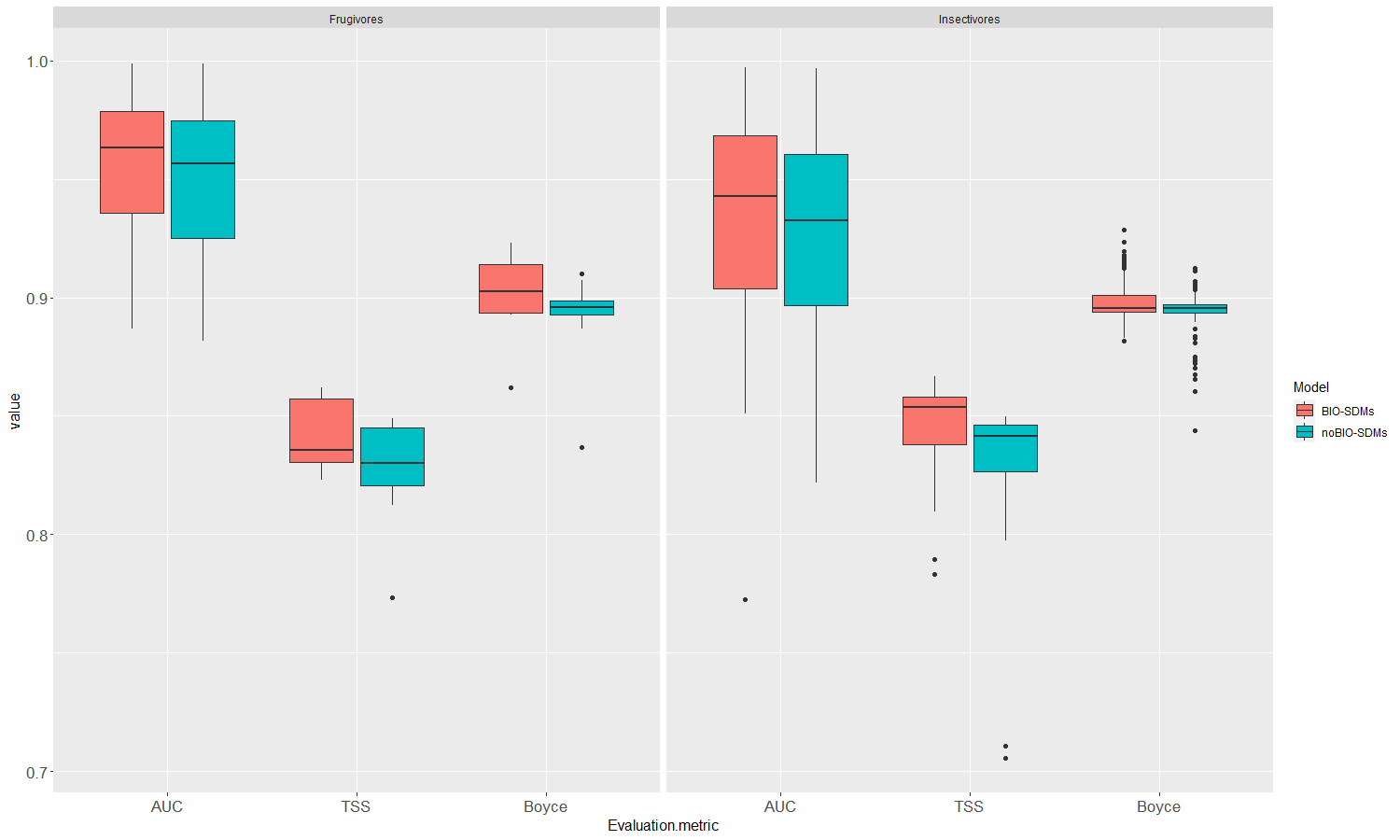
**

Table S3.11. Average and standard deviation permutation importance calculated over 62 species out of 177 for which the trophic resource variable is at the first rank of importance.

| Variable | Average  Permutation Importance | Standard Deviation Permutation Importance |
| --- | --- | --- |
| Trophic resource | 47.98 | 15.66 |
| Distance to Permanent Water | 10.26 | 9.34 |
| Precipitation of Driest Month (bio14) | 10.18 | 8.00 |
| Precipitation Seasonality (bio15) | 6.98 | 6.67 |
| Mean Temperature of Driest Quarter (bio9) | 6.78 | 6.91 |
| Human Population Density | 5.16 | 4.65 |
| Precipitation of Coldest Quarter (bio19) | 4.61 | 4.45 |
| Mean Temperature of Wettest Quarter (bio8) | 4.41 | 5.70 |
| Terrain Ruggedness Index (TRI) | 2.26 | 2.29 |
| Distance to Temporary Water | 1.38 | 1.94 |

Table S3.12. Average and standard deviation permutation importance calculated over 34 species out of 177 for which the trophic resource variable is at the second rank of importance.

| Variable | Average  Permutation Importance | Standard Deviation Permutation Importance |
| --- | --- | --- |
| Trophic resource | 23.07 | 7.03 |
| Precipitation of Driest Month (bio14) | 17.07 | 18.91 |
| Distance to Permanent Water | 16.87 | 15.57 |
| Mean Temperature of Driest Quarter (bio9) | 13.48 | 14.37 |
| Human Population Density | 6.67 | 6.70 |
| Precipitation Seasonality (bio15) | 6.16 | 8.77 |
| Precipitation of Coldest Quarter (bio19) | 5.84 | 4.61 |
| Mean Temperature of Wettest Quarter (bio8) | 4.00 | 4.69 |
| Terrain Ruggedness Index (TRI) | 3.67 | 4.97 |
| Distance to Temporary Water | 3.16 | 4.69 |

Table S3.13. Average and standard deviation permutation importance calculated over 24 species out of 177 for which the trophic resource variable is at the third rank of importance.

| Variable | Average  Permutation Importance | Standard Deviation Permutation Importance |
| --- | --- | --- |
| Mean Temperature of Driest Quarter (bio9) | 16.83 | 16.83 |
| Distance to Permanent Water | 16.61 | 13.74 |
| Trophic resource | 13.77 | 4.89 |
| Precipitation of Driest Month (bio14) | 13.31 | 15.41 |
| Precipitation Seasonality (bio15) | 13.01 | 21.46 |
| Precipitation of Coldest Quarter (bio19) | 10.30 | 11.19 |
| Mean Temperature of Wettest Quarter (bio8) | 6.35 | 11.57 |
| Human Population Density | 5.16 | 6.16 |
| Terrain Ruggedness Index (TRI) | 2.67 | 2.60 |
| Distance to Temporary Water | 2.00 | 2.37 |

Table S3.14. Percentage of species, average and standard deviation permutation importance when variables are at the first rank of importance over all 177 species in BIO-SDMs.

| Variable | Percentage of species | Average  Permutation Importance | Standard Deviation Permutation Importance |
| --- | --- | --- | --- |
| Trophic resource | 35.02 | 47.98 | 15.66 |
| Distance to Permanent Water | 19.77 | 35.67 | 10.25 |
| Precipitation of Driest Month (bio14) | 18.64 | 44.27 | 13.63 |
| Mean Temperature of Driest Quarter (bio9) | 10.17 | 39.65 | 9.57 |
| Precipitation Seasonality (bio15) | 5.65 | 45.18 | 17.80 |
| Precipitation of Coldest Quarter (bio19) | 2.82 | 37.88 | 5.21 |
| Human Population Density | 3.95 | 30.43 | 6.57 |
| Mean Temperature of Wettest Quarter (bio8) | 2.82 | 30.88 | 16.08 |
| Distance to Temporary Water | 0.56 | 22.31 | 0 |
| Terrain Ruggedness Index (TRI) | 0.56 | 27.81 | 0 |

Table S3.15. Percentage of species, average and standard deviation permutation importance when variables are at the first rank of importance over all 177 species in noBIO-SDMs.

| Variable | Percentage of species | Average  Permutation Importance | Standard Deviation Permutation Importance |
| --- | --- | --- | --- |
| Distance to Permanent Water | 39.55 | 40.67 | 11.66 |
| Precipitation of Driest Month (bio14) | 33.90 | 48.86 | 15.67 |
| Mean Temperature of Driest Quarter (bio9) | 8.47 | 43.59 | 11.02 |
| Precipitation Seasonality (bio15) | 6.78 | 44.53 | 18.48 |
| Precipitation of Coldest Quarter (bio19) | 3.95 | 38.74 | 8.15 |
| Human Population Density | 2.82 | 29.30 | 2.93 |
| Mean Temperature of Wettest Quarter (bio8) | 2.26 | 38.94 | 20.28 |
| Distance to Temporary Water | 1.69 | 38.63 | 5.96 |
| Terrain Ruggedness Index (TRI) | 0.56 | 28.79 | 0 |

Figure S3.16. Response curves (average over all species/replicates) for models calibrated including the biotic variable (BIO-SDMs) for frugivores (left) and insectivores (right) bats. Shaded areas represent 1 standard deviation. a) and b) Mean Temperature of Wettest Quarter; c) and d) Precipitation Seasonality; e) and f) Precipitation of Coldest Quarter; g) and h) Distance to temporary water; i) and j) Terrain Ruggedness Index; k) and l) Human Population Density.


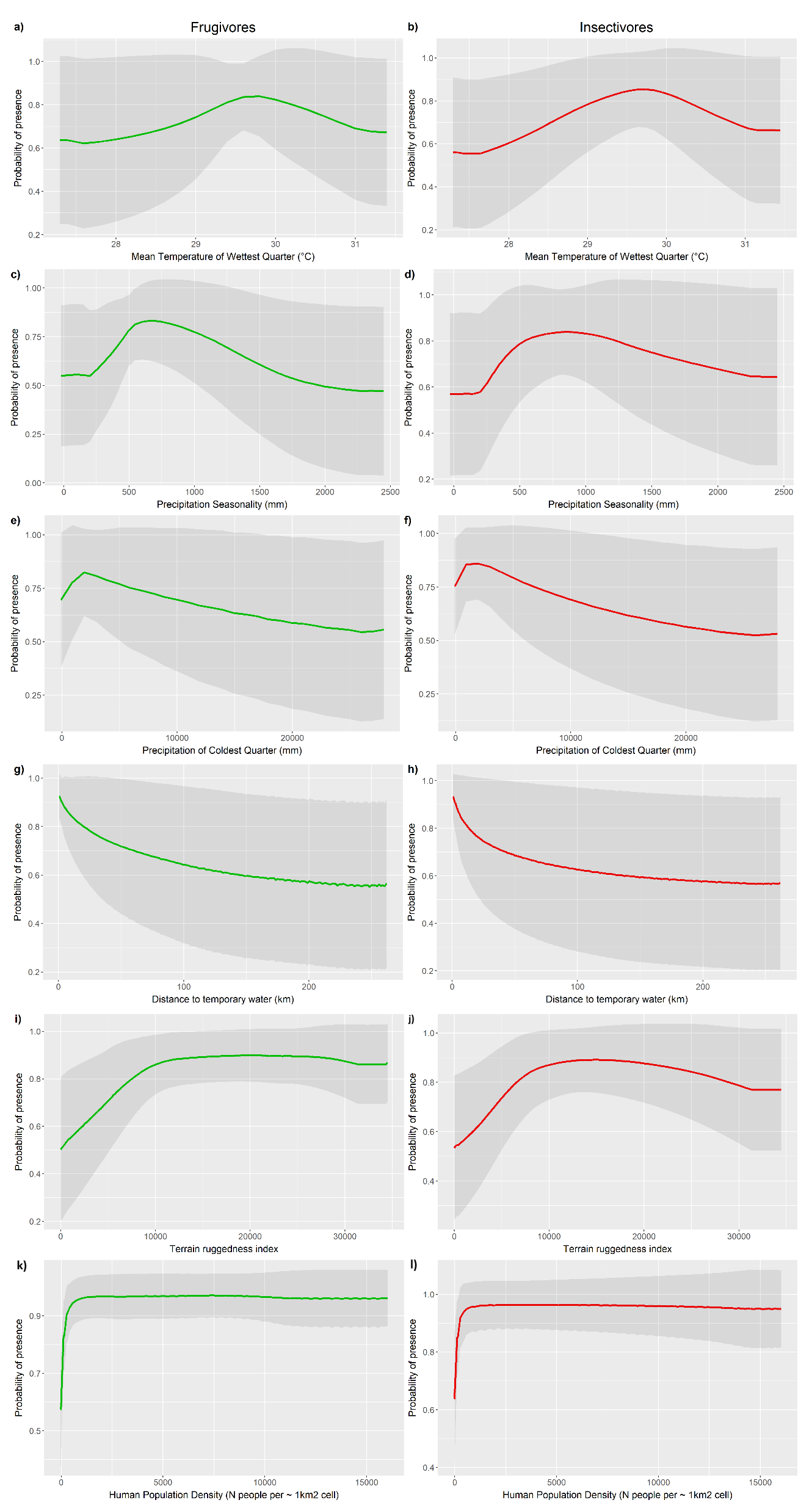


Figure S3.17. Response curves (average over all species/replicates) for models calibrated including only abiotic variables (noBIO-SDMs) for frugivores (left) and insectivores (right) bats. Shaded areas represent 1 standard deviation. a) and b) Mean Temperature of Wettest Quarter; c) and d) Mean Temperature of Driest Quarter; e) and f) Precipitation of Driest Month; g) and h) Precipitation Seasonality; Distance to temporary water; i) and j) Precipitation of Coldest Quarter; k) and l) Distance to permanent water; m) and n) Distance to temporary water; o) and p) Terrain Ruggedness Index; q) and r) Human Population Density.


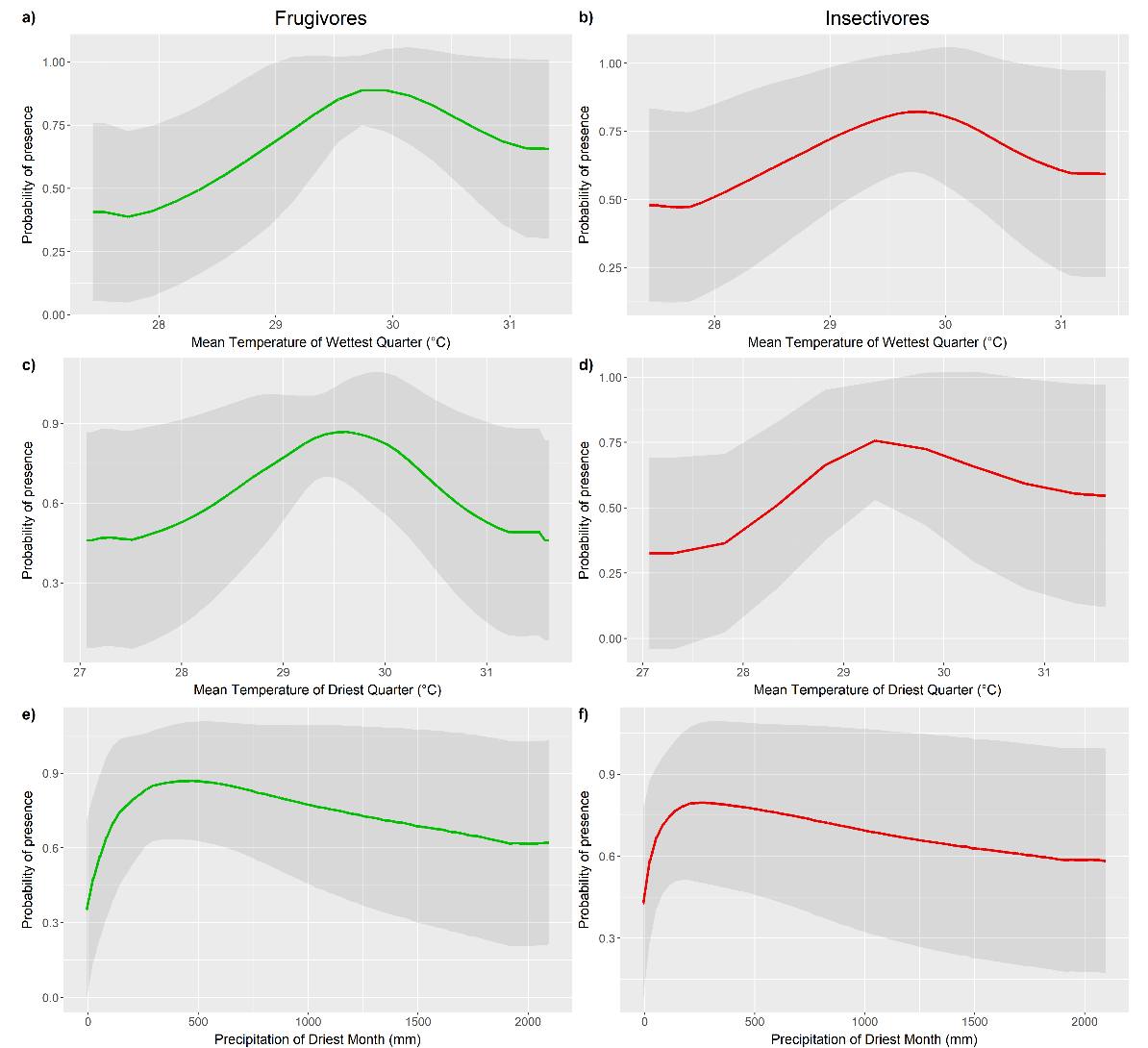


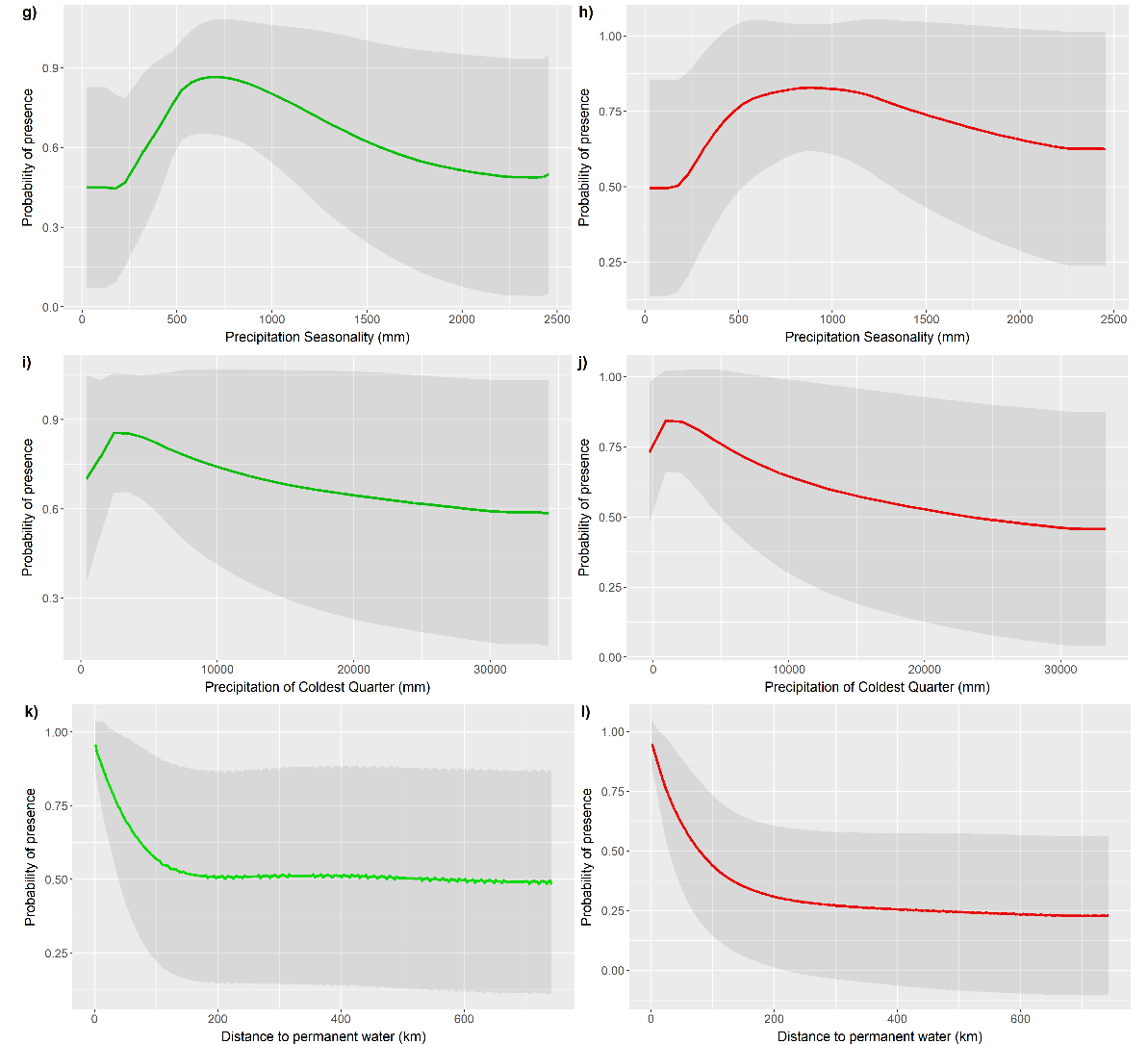

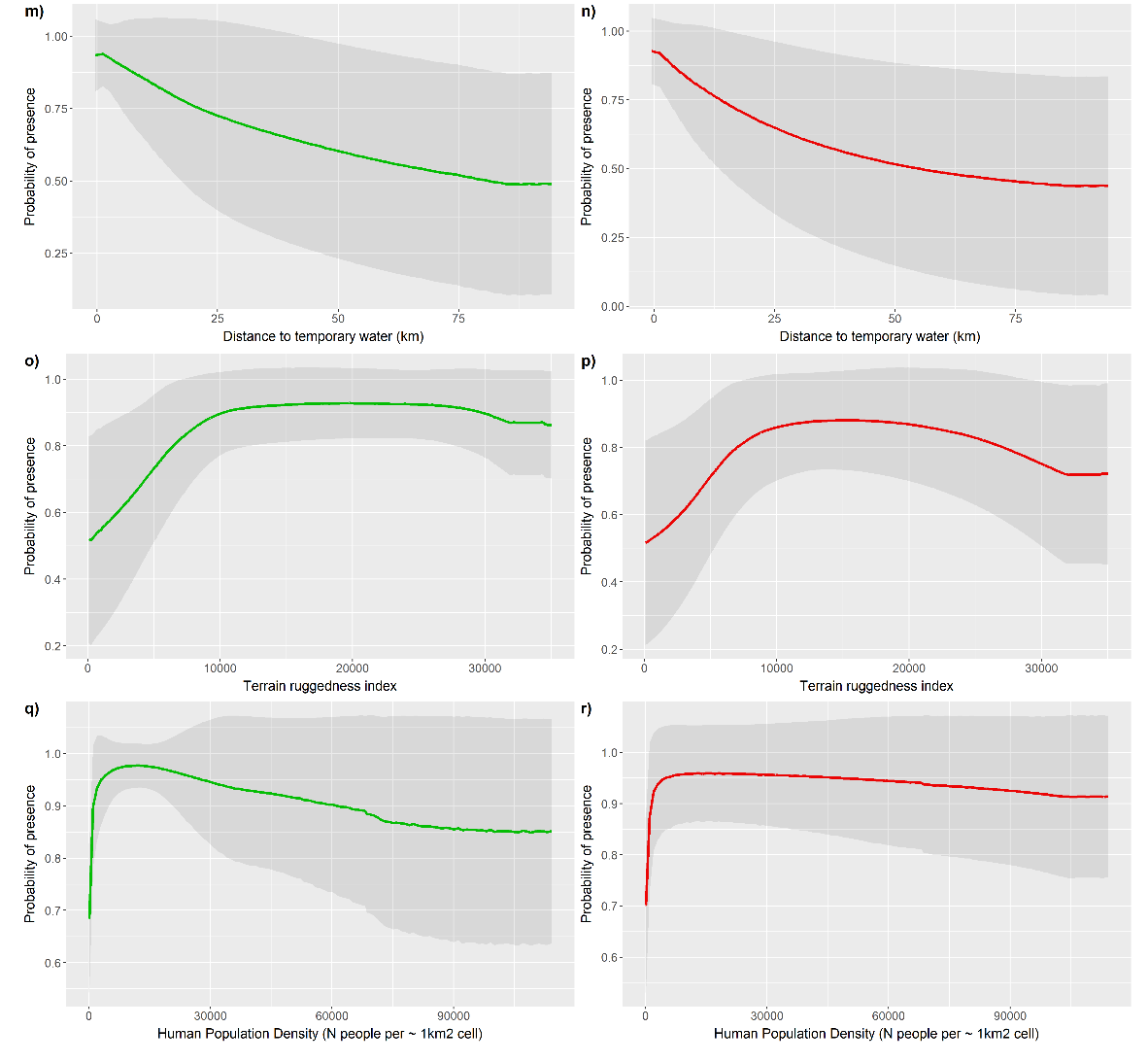


Table S3.18. Average and standard deviation permutation importance of generalist species when the trophic resource is ranked as the first (43% of species) most important variable.

| Variable | Average  Permutation Importance | Standard Deviation Permutation Importance |
| --- | --- | --- |
| Trophic resource | 47.26 | 15.46 |
| Precipitation of Driest Month (bio14) | 9.37 | 8.20 |
| Distance to Permanent Water | 8.45 | 7.87 |
| Precipitation Seasonality (bio15) | 8.13 | 7.26 |
| Mean Temperature of Driest Quarter (bio9) | 8.06 | 6.86 |
| Precipitation of Coldest Quarter (bio19) | 5.38 | 4.60 |
| Human Population Density | 5.32 | 4.62 |
| Mean Temperature of Wettest Quarter (bio8) | 4.03 | 5.33 |
| Terrain Ruggedness Index (TRI) | 2.26 | 1.90 |
| Distance to Temporary Water | 1.74 | 2.40 |

Table S3.19. Average and standard deviation permutation importance calculated over 70 generalist species.

| Variable | Average  Permutation Importance | Standard Deviation Permutation Importance |
| --- | --- | --- |
| Trophic resource | 28.59 | 20.42 |
| Distance to Permanent Water | 16.55 | 14.53 |
| Mean Temperature of Driest Quarter (bio9) | 12.26 | 13.48 |
| Precipitation of Driest Month (bio14) | 12.06 | 13.30 |
| Precipitation Seasonality (bio15) | 8.18 | 11.35 |
| Precipitation of Coldest Quarter (bio19) | 6.70 | 6.08 |
| Human Population Density | 5.82 | 5.34 |
| Mean Temperature of Wettest Quarter (bio8) | 5.34 | 8.36 |
| Distance to Temporary Water | 2.30 | 3.64 |
| Terrain Ruggedness Index (TRI) | 2.19 | 2.13 |

Table S3.20. Species-specific percentage of change in potential distribution between BIO-SDMs and noBIO-SDMs.

| Species | Percentage of change |
| --- | --- |
| *Asellia tridens* | 10.15 |
| *Cardioderma cor* | 8.42 |
| *Casinycteris argynnis* | 12.67 |
| *Chaerephon aloysiisabaudiae* | 7.75 |
| *Chaerephon ansorgei* | 9.60 |
| *Chaerephon atsinanana* | 7.78 |
| *Chaerephon bivittatus* | 4.51 |
| *Chaerephon chapini* | 5.23 |
| *Chaerephon leucogaster* | 8.81 |
| *Chaerephon major* | 13.98 |
| *Chaerephon nigeriae* | 7.19 |
| *Chaerephon pumilus* | 3.93 |
| *Chaerephon pusillus* | 0.31 |
| *Cistugo lesueuri* | 9.87 |
| *Cloeotis percivali* | 9.81 |
| *Coleura afra* | 10.42 |
| *Doryrhina cyclops* | 5.50 |
| *Eidolon dupreanum* | 10.66 |
| *Eidolon helvum* | 5.03 |
| *Epomophorus angolensis* | 6.30 |
| *Epomophorus crypturus* | 13.01 |
| *Epomophorus dobsonii* | 4.78 |
| *Epomophorus gambianus* | 9.94 |
| *Epomophorus labiatus* | 8.87 |
| *Epomophorus minor* | 8.30 |
| *Epomophorus pusillus* | 6.18 |
| *Epomophorus wahlbergi* | 3.86 |
| *Epomops buettikoferi* | 7.02 |
| *Epomops franqueti* | 7.12 |
| *Eptesicus hottentotus* | 6.30 |
| *Eptesicus isabellinus* | 3.62 |
| *Glauconycteris argentata* | 2.75 |
| *Glauconycteris beatrix* | 5.86 |
| *Glauconycteris poensis* | 5.83 |
| *Glauconycteris variegata* | 13.10 |
| *Hipposideros abae* | 12.97 |
| *Hipposideros beatus* | 3.14 |
| *Hipposideros caffer* | 6.10 |
| *Hipposideros fuliginosus* | 12.44 |
| *Hipposideros jonesi* | 18.07 |
| *Hipposideros ruber* | 5.96 |
| *Hipposideros tephrus* | 6.07 |
| *Hypsignathus monstrosus* | 8.54 |
| *Hypsugo anchieta* | 5.71 |
| *Hypsugo crassulus* | 5.38 |
| *Kerivoula argentata* | 7.77 |
| *Kerivoula lanosa* | 12.12 |
| *Laephotis botswanae* | 9.85 |
| *Laephotis wintoni* | 3.86 |
| *Lavia frons* | 5.58 |
| *Lissonycteris angolensis* | 9.58 |
| *Macronycteris commersonii* | 19.27 |
| *Macronycteris gigas* | 7.13 |
| *Macronycteris vittatus* | 8.58 |
| *Megaloglossus azagnyi* | 5.84 |
| *Megaloglossus woermanni* | 3.49 |
| *Mimetillus moloneyi* | 7.70 |
| *Miniopterus aelleni* | 25.46 |
| *Miniopterus africanus* | 7.88 |
| *Miniopterus arenarius* | 6.91 |
| *Miniopterus fraterculus* | 2.41 |
| *Miniopterus gleni* | 19.92 |
| *Miniopterus griveaudi* | 7.17 |
| *Miniopterus inflatus* | 4.68 |
| *Miniopterus mahafaliensis* | 19.67 |
| *Miniopterus majori* | 20.45 |
| *Miniopterus manavi* | 32.73 |
| *Miniopterus minor* | 6.17 |
| *Miniopterus mossambicus* | 7.35 |
| *Miniopterus natalensis* | 6.49 |
| *Miniopterus schreibersii* | 1.01 |
| *Mops brachypterus* | 4.51 |
| *Mops condylurus* | 4.81 |
| *Mops demonstrator* | 8.90 |
| *Mops leucostigma* | 19.10 |
| *Mops midas* | 4.43 |
| *Mops nanulus* | 8.55 |
| *Mops niveiventer* | 15.59 |
| *Mops spurrelli* | 7.81 |
| *Mops thersites* | 6.26 |
| *Mormopterus jugularis* | 19.23 |
| *Myonycteris leptodon* | 14.34 |
| *Myonycteris torquata* | 9.62 |
| *Myotis bocagii* | 7.42 |
| *Myotis goudotii* | 14.28 |
| *Myotis punicus* | 1.56 |
| *Myotis tricolor* | 3.65 |
| *Myotis welwitschii* | 8.23 |
| *Myzopoda aurita* | 3.27 |
| *Nanonycteris veldkampii* | 10.51 |
| *Neoromicia brunnea* | 6.11 |
| *Neoromicia capensis* | 4.21 |
| *Neoromicia grandidieri* | 4.68 |
| *Neoromicia guineensis* | 12.50 |
| *Neoromicia helios* | 7.28 |
| *Neoromicia matroka* | 20.95 |
| *Neoromicia nanus* | 6.29 |
| *Neoromicia rendalli* | 10.82 |
| *Neoromicia somalica* | 9.97 |
| *Neoromicia stanleyi* | 6.55 |
| *Neoromicia tenuipinnis* | 3.69 |
| *Neoromicia zuluensis* | 5.62 |
| *Nycteris arge* | 2.39 |
| *Nycteris aurita* | 5.48 |
| *Nycteris gambiensis* | 5.69 |
| *Nycteris grandis* | 10.51 |
| *Nycteris hispida* | 5.20 |
| *Nycteris intermedia* | 7.21 |
| *Nycteris macrotis* | 7.83 |
| *Nycteris nana* | 5.24 |
| *Nycteris thebaica* | 5.38 |
| *Nycteris woodi* | 4.34 |
| *Nycticeinops schlieffenii* | 5.96 |
| *Otomops madagascariensis* | 20.51 |
| *Otomops martiensseni* | 4.34 |
| *Otonycteris hemprichii* | 4.25 |
| *Paremballonura atrata* | 6.35 |
| *Paremballonura tiavato* | 6.82 |
| *Pipistrellus hesperidus* | 2.98 |
| *Pipistrellus kuhlii* | 6.60 |
| *Pipistrellus nanulus* | 7.08 |
| *Pipistrellus rusticus* | 5.14 |
| *Platymops setiger* | 5.58 |
| *Plecotus christii* | 3.46 |
| *Plecotus teneriffae* | 2.27 |
| *Pteropus rufus* | 8.30 |
| *Pteropus seychellensis* | 13.25 |
| *Rhinolophus alcyone* | 5.31 |
| *Rhinolophus blasii* | 2.65 |
| *Rhinolophus capensis* | 3.77 |
| *Rhinolophus clivosus* | 2.25 |
| *Rhinolophus damarensis* | 5.75 |
| *Rhinolophus darlingi* | 3.02 |
| *Rhinolophus deckenii* | 8.22 |
| *Rhinolophus denti* | 3.10 |
| *Rhinolophus eloquens* | 2.72 |
| *Rhinolophus ferrumequinum* | 1.79 |
| *Rhinolophus fumigatus* | 12.59 |
| *Rhinolophus hildebrandtii* | 3.27 |
| *Rhinolophus hipposideros* | 1.19 |
| *Rhinolophus landeri* | 19.18 |
| *Rhinolophus lobatus* | 5.19 |
| *Rhinolophus mehelyi* | 1.51 |
| *Rhinolophus rhodesiae* | 15.25 |
| *Rhinolophus simulator* | 5.50 |
| *Rhinolophus smithersi* | 6.62 |
| *Rhinolophus swinnyi* | 2.16 |
| *Rhinopoma cystops* | 5.07 |
| *Rhinopoma microphyllum* | 1.13 |
| *Rousettus aegyptiacus* | 3.14 |
| *Rousettus madagascariensis* | 11.64 |
| *Rousettus obliviosus* | 7.01 |
| *Saccolaimus peli* | 5.50 |
| *Sauromys petrophilus* | 11.79 |
| *Scotoecus albofuscus* | 17.59 |
| *Scotoecus hirundo* | 7.62 |
| *Scotonycteris bergmansi* | 12.17 |
| *Scotonycteris occidentalis* | 10.29 |
| *Scotonycteris zenkeri* | 4.49 |
| *Scotophilus dinganii* | 3.67 |
| *Scotophilus leucogaster* | 5.31 |
| *Scotophilus nigrita* | 8.93 |
| *Scotophilus nigritellus* | 7.86 |
| *Scotophilus nux* | 5.87 |
| *Scotophilus robustus* | 21.68 |
| *Scotophilus viridis* | 9.01 |
| *Stenonycteris lanosus* | 4.38 |
| *Tadarida aegyptiaca* | 2.84 |
| *Tadarida fulminans* | 4.06 |
| *Tadarida lobata* | 7.64 |
| *Tadarida ventralis* | 5.33 |
| *Taphozous mauritianus* | 4.83 |
| *Taphozous nudiventris* | 2.11 |
| *Taphozous perforatus* | 4.46 |
| *Triaenops afer* | 4.57 |
| *Triaenops menamena* | 19.28 |
| *Vansonia rueppellii* | 3.61 |

Table S3.21. Results of linear regression with a phylogenetic correction. Signif. codes: 0.0001 ‘***’ 0.001 ‘**’ 0.01 ‘*’ 0.05 ‘.’ 0.1 ‘n.s.’ 1.

| Variable | Estimate±SD | P |
| --- | --- | --- |
| Number of occurrences | -0.009±0.002 | *** |
| Number of diet items | 0.7±0.2 | *** |
| Body mass | 0.005±0.009 | n.s. |
| Colony size | -0.00005±0.00002 | * |
